# Modeling environment through a general exposome factor in two independent adolescent cohorts

**DOI:** 10.1093/exposome/osac010

**Published:** 2022-12-14

**Authors:** Tyler M Moore, Elina Visoki, Stirling T Argabright, Grace E Didomenico, Ingrid Sotelo, Jeremy D Wortzel, Areebah Naeem, Ruben C Gur, Raquel E Gur, Varun Warrier, Sinan Guloksuz, Ran Barzilay

**Affiliations:** Department of Psychiatry, Perelman School of Medicine, University of Pennsylvania, Philadelphia, PA, USA; Lifespan Brain Institute of the Children’s Hospital of Philadelphia (CHOP) and Penn Medicine, Philadelphia, PA, USA; Lifespan Brain Institute of the Children’s Hospital of Philadelphia (CHOP) and Penn Medicine, Philadelphia, PA, USA; Lifespan Brain Institute of the Children’s Hospital of Philadelphia (CHOP) and Penn Medicine, Philadelphia, PA, USA; Lifespan Brain Institute of the Children’s Hospital of Philadelphia (CHOP) and Penn Medicine, Philadelphia, PA, USA; Lifespan Brain Institute of the Children’s Hospital of Philadelphia (CHOP) and Penn Medicine, Philadelphia, PA, USA; Lifespan Brain Institute of the Children’s Hospital of Philadelphia (CHOP) and Penn Medicine, Philadelphia, PA, USA; Lifespan Brain Institute of the Children’s Hospital of Philadelphia (CHOP) and Penn Medicine, Philadelphia, PA, USA; Department of Psychiatry, Perelman School of Medicine, University of Pennsylvania, Philadelphia, PA, USA; Lifespan Brain Institute of the Children’s Hospital of Philadelphia (CHOP) and Penn Medicine, Philadelphia, PA, USA; Department of Psychiatry, Perelman School of Medicine, University of Pennsylvania, Philadelphia, PA, USA; Lifespan Brain Institute of the Children’s Hospital of Philadelphia (CHOP) and Penn Medicine, Philadelphia, PA, USA; Autism Research Centre, Department of Psychiatry, University of Cambridge, Cambridge, UK; Department of Psychiatry, Yale University School of Medicine, New Haven, CT, USA; Department of Psychiatry and Neuropsychology, School for Mental Health and Neuroscience, Maastricht University Medical Centre, Maastricht, The Netherlands; Department of Psychiatry, Perelman School of Medicine, University of Pennsylvania, Philadelphia, PA, USA; Lifespan Brain Institute of the Children’s Hospital of Philadelphia (CHOP) and Penn Medicine, Philadelphia, PA, USA; Department of Child and Adolescent Psychiatry and Behavioral Science, Children’s Hospital of Philadelphia (CHOP), Philadelphia, PA, USA

**Keywords:** mental health, child adolescent psychiatry, stress, allostatic load, obesity

## Abstract

Exposures to perinatal, familial, social, and physical environmental stimuli can have substantial effects on human development. We aimed to generate a single measure that capture’s the complex network structure of the environment (ie, exposome) using multi-level data (participant’s report, parent report, and geocoded measures) of environmental exposures (primarily from the psychosocial environment) in two independent adolescent cohorts: The Adolescent Brain Cognitive Development Study (ABCD Study, *N* = 11 235; mean age, 10.9 years; 47.7% females) and an age- and sex-matched sample from the Philadelphia Neurodevelopmental Cohort (PNC, *N* = 4993). We conducted a series of data-driven iterative factor analyses and bifactor modeling in the ABCD Study, reducing dimensionality from 348 variables tapping to environment to six orthogonal exposome subfactors and a general (adverse) exposome factor. The general exposome factor was associated with overall psychopathology (*B* = 0.28, 95% CI, 0.26-0.3) and key health-related outcomes: obesity (odds ratio [OR] , 1.4; 95% CI, 1.3-1.5) and advanced pubertal development (OR, 1.3; 95% CI, 1.2-1.5). A similar approach in PNC reduced dimensionality of environment from 29 variables to 4 exposome subfactors and a general exposome factor. PNC analyses yielded consistent associations of the general exposome factor with psychopathology (*B* = 0.15; 95% CI, 0.13-0.17), obesity (OR, 1.4; 95% CI, 1.3-1.6), and advanced pubertal development (OR, 1.3; 95% CI, 1-1.6). In both cohorts, inclusion of exposome factors greatly increased variance explained in overall psychopathology compared with models relying solely on demographics and parental education (from <4% to >38% in ABCD; from <4% to >18.5% in PNC). Findings suggest that a general exposome factor capturing multi-level environmental exposures can be derived and can consistently explain variance in youth’s mental and general health.

## Introduction

Environment (E) is a key driver of variability in human development,^1^ with extensive literature linking environmental exposures to general^2^ and mental health.^3^ Childhood environment is especially important for development, with evidence that exposures occurring during sensitive periods of development are critical for later life health^3^ and humans.^4^ Therefore, there is a clear need to characterize environment in a systematic and comprehensive manner early in the lifespan to advance our understanding of its role in human development.

There are multiple notable challenges in studying environmental influence on health and disease. First, exposures are often co-occurring and collinear,^5^ and it is difficult to disentangle specific effects because they are intertwined in a complex, dynamic network.^6^ Thus, it is difficult to dissect specificity in relationships between single exposures (eg, trauma) and developmental outcomes. Second, exposures are not isolated and are likely to interact both among themselves (environment-by-environment interaction) and with genetics (gene-by-environment interaction) to drive developmental outcomes, as proposed in various developmental models.^7-9^ Finally, it is exceptionally difficult to clearly label exposures as genetic or environmental influences, as one’s environment is reflected in genetic association studies and genetic influences help shape one’s environment.^10-12^ Hence, considering variables either purely biological or purely environmental is inaccurate.

To address the challenge of collinearity, the exposome paradigm provides a framework that may advance the study of environment.^13^ The “exposome” (see Wild 2005^14^) represents the totality of environmental exposures that an individual experiences from conception throughout the lifespan.^15^ While early studies of the association between the exposome and health focused on physical exposures (eg, chemical carcinogens) and cancer risk,^16^ the concept has been extended to include environmental exposures in a broader context (eg, socioeconomic and lifestyle factors^17^). Recently, the exposome framework has been applied in psychiatry,^18^ with evidence of exposome effects in youth psychosis^19^ and suicidal ideation.^20^

While associations between specific environmental exposures and development have long been studied, there is a need for an integrative approach able to leverage comprehensive environmental data to systematically capture the exposome, examine its relationship with health measures, and facilitate its integration into human development studies.^21^ Specifically, there is a gap in large-scale studies of the association between the exposome and child and adolescent development. The availability of rich data spanning multiple levels of environment in youth cohorts provides an opportunity to address this gap.

Here, we apply an exposome framework analysis that leverages environmental data on psychosocial exposures (ie, *psychosocial exposome*) reported by youth and their parents and on geocoded address measures in two youth datasets. First, using data from the Adolescent Brain and Cognitive Development (ABCD) Study,^22^ which included youth and parent report of children’s exposures and geocoded census-level data,^23^ we conducted a series of factor analyses to reduce the dimensionality of the data and generate exposome factor scores. Then, we generalized our exposome conceptual framework using an independent age- and sex-matched sample from the Philadelphia Neurodevelopmental Cohort^24^ (PNC). Though less environment-focused than the ABCD Study, the PNC included multiple environmental measures based on youth and parent report and census-level data.^25^

**Figure 1.**
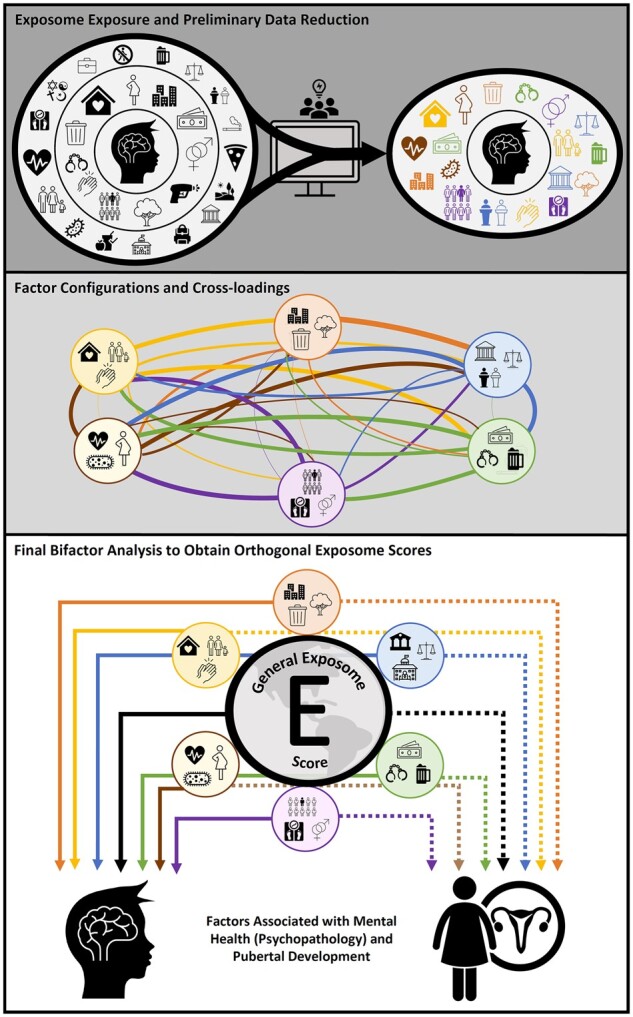
Visual presentation of study design. First, 348 environmental variables from the ABCD Study were chosen for representing the multiple dimensions of the exposome. These variables were reduced using an iterative process of exploratory factor analyses (EFAs) that identified correlated factors allowing reduction to 96 variables from multiple dimensions of environment including family, household, school, extracurricular, neighborhood and state-level and prenatal and history of antenatal exposures (top). Thereafter, these 96 combined items underwent an EFA that culminated in a final model, which finalized factor configurations and cross-loadings (middle), revealing six factors relating to the exposome (*household adversity* factor, *neighborhood environment* factor, *day-to-day experiences* factor, *state environment* factor, *family values* factor, and *pregnancy/birth complications* factor). Subsequently, these factors were subjected to confirmatory bifactor analysis, which allowed the generation of a general exposome factor (*Exp-factor*) informed by all items, in addition to six orthogonal exposome subfactors (bottom). Finally, we investigated how these exposome factors are associated with mental health, BMI, and pubertal development.

In alignment with the exposome paradigm, we aimed to (i) comprehensively and systematically characterize the psychosocial exposome (ie, the combined effect of exposures at multiple levels of analysis) of young US adolescents using two youth cohorts; (ii) calculate a general exposome factor score that represents shared multi-level environmental burden and can be used in downstream analyses; and (iii) test the exposome’s associations with indicators of mental health (ie, psychopathology) and general health (we focused on obesity, a key risk factor for later lifespan morbidity,^26^ and pubertal development, considering studies linking earlier puberty with poorer health outcomes^27^). Ultimately, we aimed to produce an overall environment factor score that would comprehensively capture the exposome and could be leveraged to broadly model environmental health risk in youth. Figure 1 depicts the overall study design.

## Methods

### Participants

The ABCD sample includes 11 878 children aged 9-10 years at baseline, recruited through school systems.^28^ For the purposes of this study, 1-year follow-up data were used (*N* = 11 235). Participants were enrolled at 21 sites, with the catchment area encompassing over 20% of the entire US population in this age group. All participants gave assent. Parents/caregivers signed informed consent. The ABCD protocol was approved by the University of California, San Diego Institutional Review Board (IRB), and was exempted from a full review by the University of Pennsylvania IRB. See Table S1 for full demographic data.

The PNC is a collaboration between the Children’s Hospital of Philadelphia (CHOP) and the Brain Behavior Laboratory at the University of Pennsylvania. Participants from the greater Philadelphia area were ascertained through the CHOP pediatric health care network. The PNC included children aged 8-21 years (*N* = 9498). For participants aged 8-10, clinical evaluation was done using a parent report. For participants 11 and older, clinical evaluation was based on an interview with the youth. For the current study, to keep with the developmental stage of the ABCD sample, we included only PNC participants under age 14 years old (*N* = 4933, see Table S2 for demographic data in comparison to ABCD Study). Participants’ written assent and parental consent were obtained. University of Pennsylvania and CHOP IRBs approved all procedures.

### Statistical analysis

The analytic plan and hypotheses were preregistered on Open Science Framework in October 2020, before the full release of ABCD Study 1-year follow-up data. Analyses were conducted from January to October 2021, following ABCD data release 3.0, which was the first full release of the 1-year follow-up data and included youth-reported life events and discrimination. We used R (package psych^29^) and Mplus 8.4^30^ for factor analyses and SPSS statistical package version 26.0 for all other statistical methods. Statistical significance was set at *P* < .05.

### Handling of missing data

Models testing associations of the exposome with psychopathology, obesity, and pubertal development used listwise deletion of missing data. All other analyses used pairwise deletion.

### ABCD study analyses

#### Measures

We included a comprehensive set of 348 environmental variables in analyses. In line with our goal to comprehensively assess the exposome, we applied a permissive definition of environment, utilizing variables from multiple levels of analysis including family-, household-, school-, extracurricular-, neighborhood-, and state-level, as well as prenatal exposures. Notably, we included measures based on both youth- and parent-report, as well as geocoded address; while certain youth-reported measures invariably capture aspects of subjective experience (eg, school enjoyment), we chose to include them to best capture environment multidimensionally. Because we wanted to investigate the utility of applying an exposome framework, we excluded two pivotal measures commonly used to estimate environment, including in previous ABCD Study research: household income^31^ and parental education.^32^ This choice allowed us (1) to test the “added value” of the exposome factor scores to explain variance in health outcomes over and above commonly used proxies of environment known to associate with developmental outcomes and (2) to validate the exposome factor scores using “classic” indicators of socioeconomic environment. Additionally, we did not include genetic data as we focused on environmental exposures in this project, nor did we include imaging or neurocognitive data. Imaging procedures and the comprehensive ABCD neurocognitive assessments were not conducted in the ABCD Study time point used in the current exposome analysis (ie, the 1-year follow-up assessment). Table S3 provides the full range of exposure measures used in the present study.

For models testing associations of exposome factor scores with psychopathology (*P-factor*), we used mental health variables comprising youth self- or caregiver-reported attitudes, experiences, and problems (93 variables, see Table S4 for the full list). For models testing associations of exposome factor scores with obesity and pubertal development, we used body mass index (BMI) and pubertal development data (measure pds_y_ss_female_category_2 and pds_y_ss_male_cat_2).

#### Dimensionality reduction of environment and generation of exposome factor scores in ABCD Study

Due to the large number of ABCD variables of multiple formats (continuous, ordinal, and nominal), different lengths (scales used in the ABCD Study ranged from 2 to 59 items in length), and multiple sources (youth-report, parent-report, census-level composites, etc.), the process of arriving at an optimal ABCD Study exposome model was complex. Figure S1 presents a visual schematic of the steps taken to reduce dimensionality of variables. We started with 348 variables tapping to environment of ABCD Study participants. We often chose to use summary scales to represent overarching culture and environment (eg, Mexican American Cultural Values Scale and family conflict) and indicators of health (eg, family psychiatric history and dietary habits). We included these in the following analysis and, using multiple exploratory factor analyses (EFAs), iteratively reduced the number of variables. See Supplementary Methods for a detailed description of the process. In total, nine iterations were run (Tables S5-S13) to arrive at a set of 96 variables with minimal redundancy.

Next, we estimated an EFA solution using the “clean” 96-variable dataset using iterated target rotation (ITR).^33^^,^^34^ With the six-factor EFA solution obtained from the ITR process, we went on to define a quasi-confirmatory bifactor analysis from which ABCD Study exposome factor scores could be obtained (“quasi-” because there is no cross-validation being performed here; the “confirmatory” model is actually being used to estimate a model for score creation rather than truly confirm a theoretical or empirically derived model^35^). The bifactor model confirmatory factor analysis (CFA) was estimated in Mplus using the wlsmv estimator, accounting for clustering by family. A bifactor model uses a factor configuration whereby each variable loads not only on its specific factor (eg, a measure of family poverty might load on a “household adversity” factor), but also on a general exposome factor comprising (with estimated loadings on) all variables. Note that this analysis reduced the included items from 96 to 65 according to significance of within-factor association (items with an absolute value of association less than 0.30 were removed) and generated a general exposome factor in addition to six (orthogonal) exposome subfactors. Fit of the model was judged based on comparative fit index (CFI), root mean-square error of approximation (RMSEA), and standardized root mean-square residual (SRMR). Additionally, see Table S14 for bifactor indices,^36^ such as explained common variance (ECV), omega-hierarchical, and factor determinacy.

Further details on the derivation of the exposome factor scores can be found in Supplemental Methods.

#### Association of exposome factor scores with demographic characteristics

Exposome factor scores were compared across demographic variables (male vs female, high vs low parent education and household income, race, and ethnicity) using *t*-tests (Bonferroni corrected for seven comparisons), with Cohen’s *d* to estimate effect size.

#### Generation of P-factor in ABCD Study

We modeled psychopathology dimensionally using the *P-factor*, a reliable measure of psychopathology in youth samples^37^ that represents life course vulnerability to psychiatric disorders^38^ and is predictive of long-term psychiatric and functional outcomes.^39^ While the exposome factor analyses required some special modeling due to the mixture of variable formats (continuous and ordinal) and expected complex structure, all psychopathology variables could be analyzed entirely within an item-factor analysis framework^40^ whereby all correlations are polychoric rather than being a mix of types. This psychopathology factor analysis (using oblimin rotation) revealed that the psychopathology items clustered exactly by instrument (ie, questionnaire/scale), with only two cross-loadings >0.30. The “clean” solution supports our use of a simple structure rotation. All items thusly grouped by instrument form a six-factor solution (see Table S15 for a full description).

The results of the configuration above were taken as the basis of the confirmatory model used to calculate the *P-factor* score using a bifactor model CFA estimated in Mplus using the wlsmv estimator, accounting for clustering by family. Table S16 details results from confirmatory bifactor model analysis, displaying specific factor loadings as well as loadings to a general psychopathology factor. Overall, fit of the model was acceptable (CFI, 0.93; RMSEA, 0.023; SRMR, 0.085), and these results are presented visually in Figure S2. This general *P-factor* score was used for subsequent correlational analyses with the exposome factor scores.

#### Associations of exposome factor scores with psychopathology in ABCD Study

We tested the association of exposome factor scores (*Exp-factor* and six orthogonal subfactors) with the *P-factor* (dependent variable in the main analysis) using a linear regression model with the seven exposome factors as independent variables and age, sex, parent education, household income, race (White, Black, and other), and Hispanic ethnicity as covariates. The model was also run without the exposome factors to estimate the change of adjusted *R*^2^ upon addition of exposome factor scores to the model.

#### Association of exposome factor scores with obesity and pubertal development in ABCD Study

We tested the association of exposome factor scores (*Exp-factor* and six orthogonal subfactors) with obesity or pubertal development (two separate models) using binary logistic regression models with obesity (binary variable, BMI ≥ 95th percentile) or advanced pubertal status (binary variable, late/post-pubertal status [4/5 on a 5-point Likert scale of pubertal development] vs pre-/early/mid-pubertal status [1-3 on the Likert scale]) as the dependent variables, and the seven exposome factors as independent variables, co-varying for age, sex, parental education, household income, race (White, Black, and other), and Hispanic ethnicity. The pubertal development model also co-varied for BMI.

#### Sensitivity analyses

We conducted sensitivity analyses in which we used other mental health measures as dependent variables instead of the *P-factor*. We ran linear regression models with parent-reported child psychopathology (total child behavior checklist [CBCL] *t*-score) and binary logistic regression models with binary diagnoses of depression or attention deficit hyperactivity disorder (ADHD) based on the Kiddie Schedule for Affective Disorders and Schizophrenia (K-SADS) interview, chosen as more clinically interpretable outcomes representative of both internalizing and externalizing symptomatology. All models included exposome factors as the independent variables and the same covariates as in main analyses (age, sex, race, ethnicity, household income, and parent education).

We also conducted sensitivity analyses for models exploring general health outcomes, using linear regression models with continuous BMI percentile score or continuous pubertal development scale score as dependent variables. In both models, exposome factors were the independent variables and covariates were identical to main analyses (age, sex, race, ethnicity, household income, and parent education, with BMI as an additional covariate in the pubertal development model).

Lastly, to account for clustering within site and family, we ran mixed-effects regression models for both mental health (*P-factor* and CBCL scores) and general health measures (BMI and pubertal development scale), with random intercepts for site and family using the lmer() function in the lmerTest package.

### PNC analyses

#### Measures

We included all relevant environmental exposures in the PNC (*n* = 29). As with the ABCD Study, we used a permissive definition of environment and considered family history of psychiatric disorders an environmental exposure. The exposures included family history of psychiatric disorders (based on the abbreviated version of the Family Interview for Genetic Studies^41^), an indicator parental separation or divorce, traumatic experiences (assessed with a screener for eight traumatic experiences [yes/no items] that fulfill criterion A in post-traumatic-stress-disorder diagnosis), census neighborhood (block-group-level) measures derived from participants’ geocoded address,^25^ and two items related to early life: birth complication and history of lead exposure (both binary yes/no items).

For psychopathology measures, we used lifetime history of psychopathology symptoms evaluated by trained and supervised bachelor’s- and master’s-level assessors who underwent rigorous standardized training and certification using a structured screening interview,^24^ based on the K-SADS.^42^ For models testing associations of exposome factor scores with general health, we used obesity and pubertal development, as in the ABCD Study analyses.

#### Generation of exposome factor scores in PNC

To model the exposome in PNC, we assembled all environmental variables that were collected as part of the PNC assessment (*n* = 29). Generation of the PNC exposome factor scores was done using a confirmatory bifactor model, generating a PNC-derived general exposome factor and four exposome subfactors. Fit of the model was judged based on the same indices as described above for the ABCD Study portion (CFI, RMSEA, and SRMR). Additionally, see Table S14 for bifactor indices, such as ECV, omega-hierarchical, and factor determinacy.

#### Generation of P-factor in PNC

Generation of *P-factor* scores in the PNC was conducted using item-wise (ie, symptom-level) psychopathology responses (*n* = 110) from the clinical interview across all assessed psychopathology domains as previously described,^43^ similar to the methods described above for the generation of *P-factor* score in ABCD Study.

#### Association of exposome factor scores with psychopathology, obesity, and pubertal development in PNC

After the generation of the exposome factors, we followed the same approach as in the ABCD Study and tested the association of the exposome factor scores with the *P-factor* (linear regression) and with obesity (BMI ≥ 95th percentile) or advanced pubertal status (binary logistic regression). In the pubertal development model, we limited the PNC sample to ages 10-12 (*n* = 1496) to minimize the large age effect sizes present when in the 8-13 age range of the full PNC generalization sample. Models co-varied for age, sex, race (White, Black, and other), Hispanic ethnicity, and parental education. The pubertal development model also co-varied for BMI.

#### “Harmonized” models across ABCD Study and PNC

In attempt to maximize similarity between the two datasets, we ran similar regression models (linear for continuous measures and binary logistic for binary measures) with exposome factors as independent variables co-varying for measures that were available in both the ABCD Study and PNC: age, sex, race, ethnicity, and parental education. Of note, in both studies, data were already collected at the time of the current analyses, such that analyses could not be truly harmonized; rather, we tried to use similar measures as much as possible.

## Results

### Dimensionality reduction of environment in ABCD Study

We began dimensionality reduction by including 348 variables in analysis and, using 9 EFAs, iteratively reduced these to 96 with minimal redundancy. Table 1 shows the results of the final EFA of the minimally redundant 96 environmental variables, using ITR designed to detect complex structure (cross-loadings), which revealed six factors (*household adversity*, *neighborhood environment*, *day-to-day experiences*, *state environment*, *family values*, and *pregnancy/birth complications*; see Table 1 for a full description of the six-factor solution).

**Table 1.** EFAs of the optimized collection of exposome items in the ABCD Study using ITR

Item	House-hold	Neighbor-hood	Day-to-day	State	Family values	Pregnancy/birth complications
Prenatal exposure to tobacco or marijuana	**0.72**					
Parental lifestyle issues (eg, trouble with holding job, police, and alcohol use)	**0.69**					
Physical conflict among adults at the home	**0.64**					
Prenatal exposure to hard drugs (eg, cocaine and heroin)	**0.56**					
Severe maternal mental health issues (eg, breakdowns, delusions, and hospitalizations)	**0.46**					
Planned pregnancy	**−0.45**	**−**0.23				
Severe family poverty (eg, inability to afford necessities)	**0.45**	0.31				
Parent-reported sexual abuse	**0.44**					
Caregiver psychopathology (eg, mood, personality, and attention disorders)	**0.41**					
Parental separation	**0.40**					
Enforced family rules for smoking cigarettes	**−0.40**					
Family legal trouble (eg, arrests and jailtime)	**0.38**		**−**0.30			
Inability to afford necessary medical/dental visit	**0.38**					
Prenatal exposure to alcohol	**0.36**	**−**0.21			**−**0.20	
Parent-reported childhood trauma (eg, accident, disaster, and extreme violence)	**0.35**					
Sudden death of a loved one	**0.34**					
Severe paternal mental health issues (eg, breakdowns, delusions, and hospitalizations)	**0.33**					
Prenatal exposure to caffeine	**−**0.30					
Ease of access to marijuana	0.30					
Mean parental age at birth	**−**0.26					
Parent-reported family conflict	0.20					
Blood pressure complications at birth (eg, Rh incompatibility and necessary blood transfusion)	0.17					
Traumatic brain injury	0.16					
Significant family lifestyle change (eg, move and birth of new baby)	0.15					
Severe fever during first year of life	0.14					
Bed wetting	0.14					
Census-derived neighborhood poverty (eg, unemployment rate and families/individuals below poverty level)		**0.68**				
Census-derived neighborhood population density		**0.68**				
Parental ability to speak English	0.29	**−0.66**				
Census-derived neighborhood immigration and crowding		**0.60**				
Census-derived neighborhood lead exposure risk		**0.51**			**−**0.24	
Census-derived neighborhood walkability index		**0.51**				
Parent-reported neighborhood safety		**−0.47**				
Census-derived neighborhood air pollution (NO2, PM25)		**0.46**				
Crime reports-derived crime prevalence (eg, drug possession or sale and violent crime)		0.29				
Blood oxygen complications during pregnancy (eg, severe anemia)		0.26				
Parent-reported importance of independence and self-reliance		0.25				
Parent-reported interest in ethnic background and culture		0.22				
Census-derived neighborhood proximity to major roads		**−**0.21				
Participation in extracurricular activities (eg, sports, crafts, and hobbies)		**−**0.21				
Parent-reported connection to ethnic background and culture		0.19				
Nutrition		0.16				
Weeks post-conception at discovery of pregnancy		0.10				
Youth-reported positive school involvement			**0.59**			
Youth-reported acceptance and love by primary caregiver			**0.57**			
Youth-reported school enjoyment			**0.57**			
Youth-reported racial/ethnic discrimination (past year)			**−0.57**			
Youth-reported school grades and achievement			**0.55**			
Youth-reported parental monitoring and communication			**0.54**			
Youth-reported unfair treatment on racial/ethnic grounds (lifetime)			**−0.50**			
Youth-reported positive feedback at school			**0.49**			
Youth-reported acceptance and love by secondary caregiver			**0.49**			
Youth-reported family conflict			**−0.49**			
Youth-reported lesbian, gay, and bisexual discrimination (past year)			**−0.46**			
Youth-reported discrimination based on weight (past year)			**−0.45**			
Youth-reported discrimination based on being foreign (past year)		0.29	**−0.38**			
Youth-reported family discordance (eg, loss of job, mental health issues, and conflict/violence)			**−0.34**			
Youth-reported neighborhood safety		**−**0.25	**0.33**			
Youth-reported exposure to serious injury, illness, and death (self or other)			**−0.31**			
Youth-reported exposure to mature entertainment (eg, M-rated video games and R-rated movies)			**−**0.26			
Youth-reported hours of screen time per day			**−**0.23			
Youth-reported ratio of good-to-bad life events (self-rated)			0.19			
State-level indicators bias against sexual orientation		**−**0.31		**0.89**		
State-level indicators of sexism				**0.80**		
State-level marijuana laws				**0.77**		
State-level indicators of bias against immigrants		**−**0.35		**0.75**		
State-level indicators of racism				**0.70**		
State-level legality of medical marijuana				**0.67**		
Census-derived neighborhood wealth (eg, median mortgage, rent, and income)	**−**0.26			**−0.45**		
Parental bi- or multi-lingualism				**−**0.27		
Months breastfed				**−**0.21		
Ease of access to hard drugs				**−**0.17		
Family rules for using marijuana					**0.80**	
Family rules for drinking alcohol					**0.76**	
Family rules for smoking cigarettes					**0.74**	
Parent-reported importance of religion		0.28		0.27	**0.49**	
Parent-reported importance of coherence to the family unit		0.35			**0.46**	
Parent-reported importance of family support		0.27			**0.45**	
Parent-reported importance of obligation to family		0.31			**0.41**	
Family religiosity (eg, attendance to religious services)				0.23	**0.36**	
Ease of access to alcohol or tobacco					**−**0.26	
Enforced family rules for drinking alcohol					0.20	
Youth-reported ostracization from American society (lifetime)					0.18	
Premature birth						**0.82**
Twin brother or sister						**0.78**
Blood oxygen complications at birth (eg, jaundice and supplemental oxygen)						**0.60**
Time after birth in an incubator						**0.52**
Birth by caesarian section						**0.50**
Placental complications during pregnancy (eg, previa, abruptio, and persistent proteinuria)						**0.46**
Blood pressure complications during pregnancy (eg, pregnancy-related high blood pressure and diabetes)						**0.44**
Amount of prenatal care						**0.43**
Circulation complications at birth (eg, blue and slow heartbeat at birth)						**0.31**
Prenatal exposure to prescription medications						0.27
Developmental delay (motor/verbal)						0.24
Prenatal exposure to prenatal vitamins						0.21
Severe illness/infection during first year of life						0.13
	Inter-factor correlations					
	F1	F2	F3	F4	F5	F6
	1	0.16	**−**0.32	0.15	**−**0.01	0.01
	0.16	1	**−**0.2	0.16	0.09	**−**0.15
	**−**0.32	**−**0.2	1	**−**0.15	**−**0.06	**−**0.01
	0.15	0.16	**−**0.15	1	0.17	0.03
	**−**0.01	0.09	**−**0.06	0.17	1	0.02
	0.01	**−**0.15	**−**0.01	0.03	0.02	1

Results of EFAs of the final set of exposome items, using ITR designed to detect complex structure (cross-loadings). *Factor 1* comprises variables most related to household adversity, based primarily on parent-report, with the strongest indicators being the mother’s use of tobacco or marijuana during pregnancy, parental alcohol-related problems affecting ability to hold a job or stay out of jail, and frequent adult conflict in the house. *Factor 2* comprises variables most related to neighborhood environment, based primarily on geocoded address, with the strongest indicators being census-derived measures of neighborhood poverty and population density. *Factor 3* comprises variables most related to youth-reported day-to-day experiences, both positive (eg, feeling “involved at” and enjoying school, acceptance by caregivers) and negative (eg, experiences of discrimination and family conflict). *Factor 4* comprises variables most related to state environment (ie, environmental factors from the state-level), with the strongest indicators being negative attitudes toward persons with non-hetero sexual orientation, traditional views about the roles of women, and less permissive marijuana laws. Note that a “ruralness” aspect of *Factor 4* is evident in the low neighborhood wealth and property values (seventh indicator from top). *Factor 5* comprises variables most related to family values, with the strongest indicators being the strictness of rules related to alcohol, tobacco, and marijuana, as well as various indicators that tap importance of religion and family cohesiveness. *Factor 6* includes variables most related to pregnancy and birth complications, with the strongest indicator being premature birth. Of note, prenatal exposure to substances did not load on *Factor 6*, but rather on *Factor 1* which taps household adversity. This configuration was used because it indicates that maternal substance use is more revealing of household adversity than of pregnancy or birth complications. Inclusion of maternal substance use in *Factor 6* would, paradoxically, increase the ambiguity of that factor. Loadings of items with absolute value equal or greater than 0.3 are marked in bold. Inter-factor correlations are shown at the bottom of the table. Abbreviations: EFA= exploratory factor analysis. ITR= iterative target rotation.

### Generation of exposome factor scores in ABCD Study

**Figure 2.**
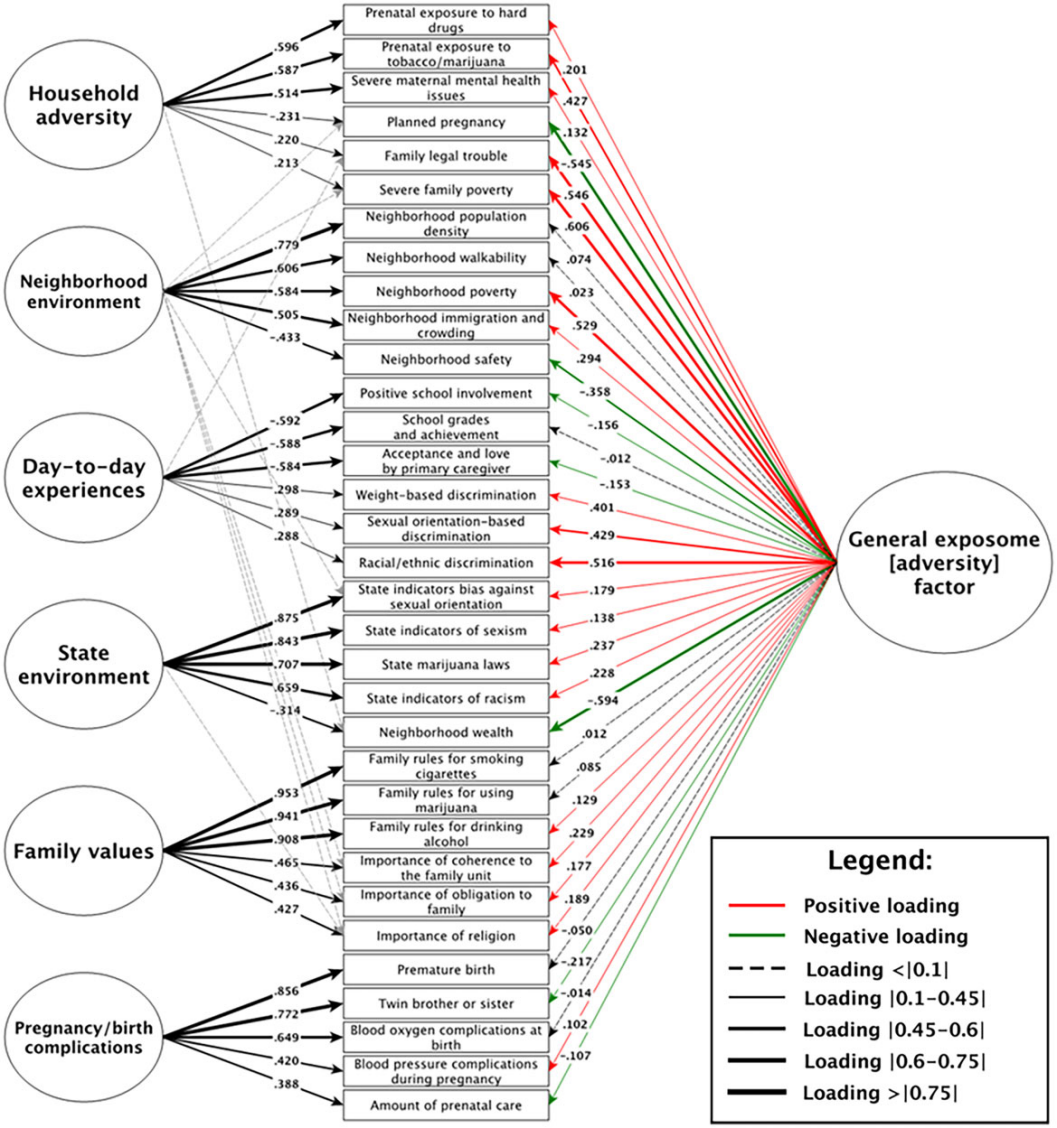
Exposome bifactor model of the ABCD Study. Only the top three items loading within-factor and on the *Exp-factor* are included; that is, a specific factor’s indicators were included in the diagram if they were among the top three strongest-loading items on that specific factor *or* on the general factor (so maximum possible = six indicators per factor in the diagram). Arrow thickness relates to the strength of the loading (higher the loading, thicker the arrow). Arrow color relates to the sign of the loading—a red arrow corresponds to positive loading (associated with a higher *Exp-factor* score; risk factor) and a green arrow corresponds to negative loading (associated with a lower *Exp-factor* score; protective factor). Subfactors are presented from top to bottom in order from F1 to F6. See **Table S17** for the full list of items and their loadings, and for the breakdown of variables that make up each factor in the bifactor model.

To estimate a general exposome factor (*Exp-factor*) score and orthogonal exposome subfactor scores that allow delineation of discrete environmental effects on development, we applied a bifactor modeling approach.^44^Figure 2 shows the results of the quasi-confirmatory bifactor analysis with the loadings of the strongest items and their direction (see full list of item loadings in Table S17).

Fit of the model was acceptable,^45^ with a RMSEA of 0.033 and SRMR of 0.060; confidence intervals around the RMSEA were imperceptibly narrow at this sample size. Note that the CFI of 0.85 was below the acceptable range, conflicting with other fit indices, which is a known phenomenon in large models^46^ and likely does not indicate poor fit.^47^ Here, it was possible to achieve a CFI >0.90 post hoc by allowing some residuals to correlate, but we opted to leave the model “pure” rather than use modification indices^48^ merely to increase one fit index.

Thus, the *Exp-factor* captures the broad, multidimensional environmental phenotyping of the ABCD Study assessment. Notably, extreme household poverty, parental legal trouble, unplanned pregnancy, physical conflict among adults in the household, neighborhood poverty, and experiences of discrimination were among the strongest loading items of the *Exp-factor*. Also, of note, in the EFA model, experiences of discrimination loaded strongly on the *day-to-day experiences* subfactor, but in the bifactor model, variance explained in the discrimination items “shifted” from *day-to-day experiences* subfactor to the *Exp-factor*. Thus, in the final model, most discrimination is accounted for by the *Exp-factor* score. The *day-to-day experiences* subfactor is left without discrimination and is heavily influenced by attitudes toward school, a center-point of life in this age range.

### The exposome across sociodemographic groups in ABCD Study

**Figure 3.**
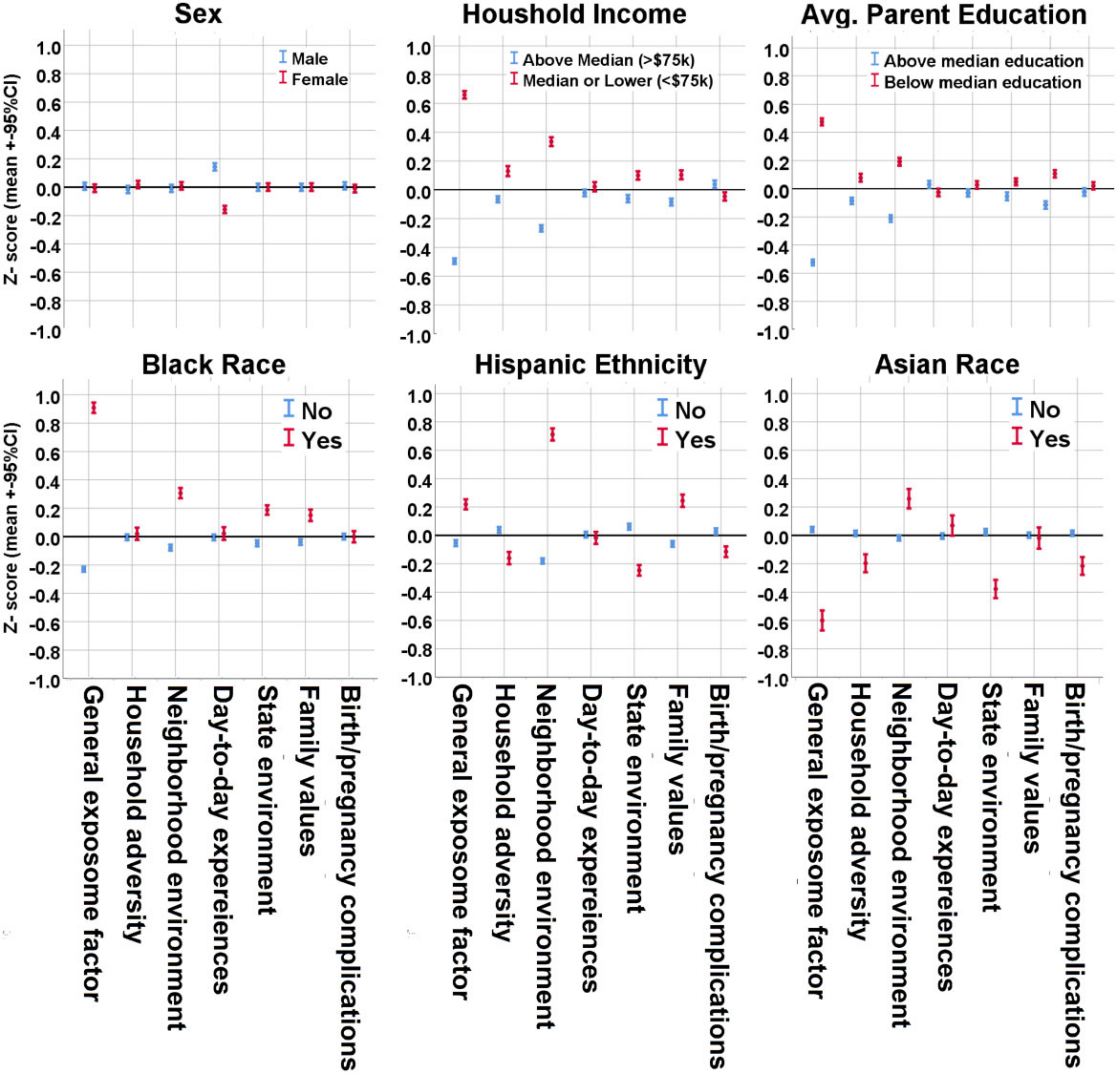
Exposome factor scores across demographic comparisons in the ABCD Study. Exposome factor scores for the six orthogonal subfactors and one general factor are compared across demographic groups. Displayed are differences between male and female participants, high and low household income, and high and low parent education (top), and Black race, Hispanic ethnicity, and Asian race (bottom). Demographic differences serve as an initial validation for use of generated exposome factor scores.

We tested the associations of the *Exp-factor* and exposome subfactor scores with key sample demographics. Figure 3 shows comparisons of the exposome factors across sex, household income, parental education, race, and ethnicity. Sex differences did not emerge in the *Exp-factor* or in five of the six subfactors; the only difference was that males had greater *day-to-day experiences* scores (Cohen’s *d *=* *0.30, *P* < .001), driven by the fact that males report disliking school more often than females do. Comparison of high-to-low parental education and household income revealed expected differences, whereby lower parent education and household income were associated with greater *Exp-factor* score with very large effect sizes (*d *=* *1.16 and *d *=* *1.40, respectively; *P*’s < .001), and greater *neighborhood environment* (poverty) scores with medium effect size (*d *=* *0.41 and *d *=* *0.63, respectively; *P*’s < .001). Comparison of high/low parental education and household income for other exposome subfactors including *household adversity*, *family values*, and *state environment* revealed differences in the small effect size range (*d*’s ranging from 0.10 to 0.22, *P*’s < .001). Notably, comparing high/low parental education and household income revealed either very small (*d*’s < 0.09) or non-significant differences in the *day-to-day experiences* subfactor and the *pregnancy/birth complications* subfactor.

Comparison of the *Exp-factor* across race and ethnicity revealed substantial differences. Black participants (*n* = 2269) had greater *Exp-factor* scores than non-Black participants (*n* = 8966) in the very large effect size range (*d *=* *1.28, *P* < .001); Hispanic participants (*n* = 2226) also showed greater *Exp-factor* scores than non-Hispanic participants (*n* = 8872), but with a smaller effect size (*d *=* *0.29, *P* < .001). Notably, Asian participants (*n* = 723) had lower *Exp-factor* scores than non-Asian participants (*n* = 10 512), with a medium-to-large effect size (*d *=* *0.66, *P* < .001). Comparisons of exposome subfactors across race and ethnicity showed that the only difference with a large effect size was observed in Hispanic participants, who had a greater *neighborhood environment* subfactor score (representing greater population density and, to a lesser extent, poverty) (*d *=* *0.92, *P* < .001). Similarly, Black and Asian participants showed greater *neighborhood environment* subfactor scores, but with smaller effect sizes (*d *=* *0.41 and *d *=* *0.28, respectively; *P*’s < .001). Comparison of the *state environment* subfactor revealed differences across race and ethnicity at the small-to-moderate effect size range (*d*’s ranging from 0.25 to 0.43). Differences in *family values* subfactor scores were observed among Black and Hispanic, but not Asian participants, who were the only group that showed differences in the *birth/pregnancy complications* subfactor, having lower scores. Notably, no differences were observed in the *day-to-day experiences* subfactor (largely determined by attitudes toward school) when comparing across race and ethnicity.

### Association of exposome factor scores with psychopathology in ABCD Study

We next sought to use exposome factor scores to explain variance in participant mental health. First, we calculated a single general factor score that represents the overall liability to psychopathology (*P-factor*),^38^ which was consistently shown to accurately represent psychopathology in youth samples.^37^ Then, we used the exposome factors as independent variables to test their contribution to explaining variance in *P-factor* score (dependent variable). We found that while age, sex, race, ethnicity, household income, and parent education explained <4% of the variance in *P-factor* score, the addition of the exposome factors increased the variance explained ∼10-fold to 38.2% (Table 2). Among the exposome factors, the *day-to-day experiences* subfactor showed the greatest association with *P-factor* score (standardized beta [*B*] = 0.516, *P* < .001), followed by the *Exp-factor* (*B* = 0.276, *P* < .001). Other exposome subfactors were also significantly associated with *P-factor* score, but with relatively modest effect sizes (all *B*’s < 0.09, all *P*’s < .025). The single subfactor not associated with *P-factor* score was *pregnancy/birth complications* (*P* = .075).

**Table 2.** Association of exposome factor scores to psychopathology *P-factor* score in the ABCD Study

	Model 1 (Demographics)			Model 2 (Demographics + Exposome)		
	Beta	SE	*P*	Beta	SE	*P*
Age (months)	**−**0.009	0.010	.363	0.007	0.008	.383
Female sex	**−**0.098	0.009	<.001	**−**0.017	0.008	.031
White race	0.020	0.014	.143	**−**0.012	0.011	.277
Black race	0.075	0.013	<.001	**−**0.014	0.011	.224
Asian race	**−**0.012	0.008	.232	0.001	0.007	.870
Hispanic ethnicity	0.007	0.010	.491	0.033	0.009	<.001
Parent education (years)	**−**0.057	0.013	<.001	**−**0.002	0.011	.852
Household income (ordinal)	**−**0.097	0.013	<.001	0.035	0.012	.003
General exposome adversity				0.285	0.011	<.001
Household adversity				0.083	0.008	<.001
Neighborhood environment				**−**0.021	0.009	.024
Day-to-day experiences				0.518	0.008	<.001
State environment				0.027	0.008	.001
Family values				**−**0.019	0.008	.018
Pregnancy/birth complications				0.014	0.008	.075
Adjusted *R*^2^	0.039			0.382		

Effect sizes (standardized betas) derived from a linear regression model testing association of demographics and exposome factors with general psychopathology (*P-factor*).

### Association of exposome factor scores with obesity and pubertal development in ABCD Study

**Figure 4.**
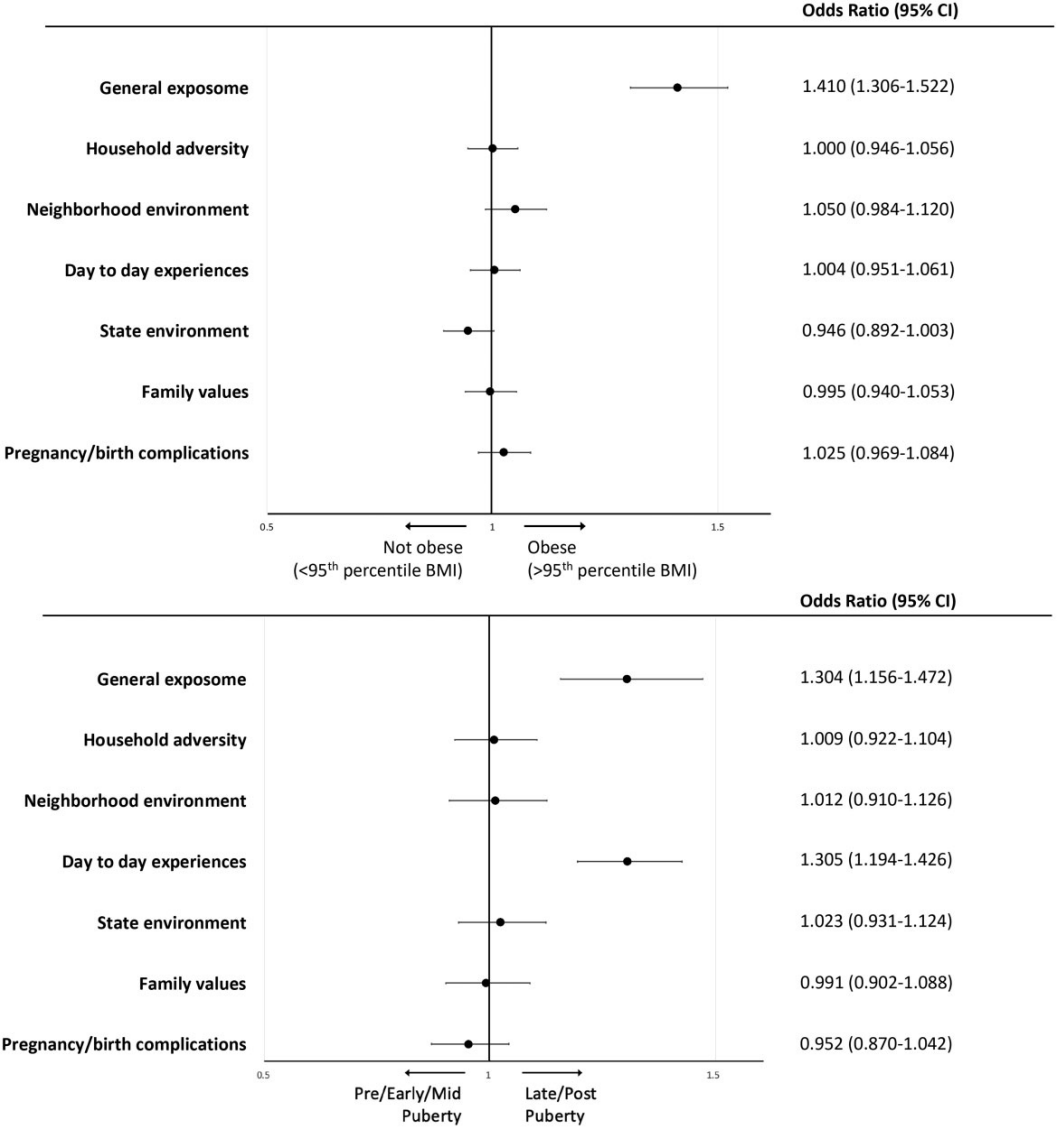
Association of exposome factor scores with obesity and pubertal development in the ABCD Study. Association of the exposome factor scores with obesity (binary variable, BMI ≥ 95th percentile, top) and late or post-pubertal stage (binary variable, contrasted against pre-, early, and mid-pubertal stage, bottom). ORs were extracted from a binary logistic regression model with exposome factor scores as independent variables, co-varying for age, sex, race (White, Black, and other), ethnicity (Hispanic), parent education, and household income. Puberty model also co-varies for BMI.

Lastly, we tested whether exposome factor scores were associated with general adolescent health indicators important to health later in the lifespan: obesity^26^ and pubertal development,^27^ both of which are influenced by the environment.^49^^,^^50^ Overall, 1871 (16.7%) in the cohort were obese based on US Centers for Disease Control (CDC) definition (BMI ≥ 95th percentile^51^). Notably, obesity prevalence in ABCD participants was consistent with nationally representative data reporting a prevalence of 15.5% obesity among US high-school youth based on CDC data.^52^ In total, 727 youths (6.5% of sample, *n* = 104 males [1.7% of males], *n* = 623 females [11.5% of females]) were late/post-pubertal (4/5 on a 5-point Likert scale). The *Exp-factor* was significantly associated with obesity (odds ratio [OR], 1.41; 95% CI, 1.31-1.52, *P* < .001) and with late/post-pubertal status (OR, 1.30; 95% CI, 1.16-1.47; *P* < .001; Figure 4 and Tables S18 and S19). No exposome subfactors were associated with obesity. The *day-to-day experiences* subfactor was the only one significantly associated with late/post-pubertal status (OR, 1.31; 95% CI, 1.19-1.43; *P* < .001).

### Sensitivity analyses

We conducted multiple sensitivity analyses in the ABCD Study to assess robustness of our main findings. We first aimed to test whether the association between exposome factors and mental health depended on the measure used to model psychopathology. We tested the associations of exposome factors with parent-reported child psychopathology (CBCL *t*-score). Like main analyses, addition of the exposome factors increased the explained variance by ∼7-fold to 17.8%, compared with 2.5% in the model relying on demographics, household income, and parent education alone (Table S20). In addition, we tested the associations of exposome factors with diagnoses of depression and ADHD. Like models using dimensional psychopathology, exposome factors were associated with both diagnoses (Table S21).

We also tested the associations of exposome factors with continuous measures of weight (BMI percentiles) and pubertal development (5-point Likert scale), rather than with binary measures. Results were similar in direction and statistical significance to main analyses (Tables S22 and S23).

Finally, we conducted sensitivity analyses that accounted for potential site and family relatedness effects in the ABCD Study. Because we wanted to evaluate environment based on factors that are included in the comprehensive ABCD assessment (and not based on site), we did not account for site in our main analyses. In sensitivity analyses, we ran mixed models testing the associations between exposome factors and mental health (*P-factor* and CBCL) and general health measures (BMI and pubertal development) accounting for site and family clustering. Results revealed similar findings to main analyses (Tables S24-S27), except for the anticipated loss of statistical significance of the *state environment* subfactor effects (which depend on site since the ABCD Study included 21 sites from different states across the United States).

### Generalization of the exposome framework in PNC

#### Generation of exposome factor scores in PNC

To test the generalizability of the exposome framework outside of the ABCD Study, we employed a confirmatory analytic approach in an independent US youth dataset—the PNC, which was sampled between 2009 and 2011,^24^ more than 5 years before the onset of the ABCD Study. We age-matched the PNC generalization sample by limiting the age of PNC participants to under 14 years, resulting in a total *N* = 4993 participants with a mean age of 10.9 years, like the ABCD sample. Besides similar age and gender distribution, the PNC sample displayed notable differences compared with the ABCD sample, including a greater proportion of Black participants (31.6% in PNC vs 20.2% in ABCD Study) and a smaller proportion of Hispanic participants (7.3% in PNC vs 20.1% in ABCD Study). Notably, the PNC was a single-site study (compared the 21-site ABCD Study).

Like in the ABCD Study, we performed a bifactor CFA of all PNC exposures to obtain acceptable model fit. Indeed, fit of the model was acceptable,^45^ with a RMSEA of 0.036 ± 0.001, SRMR of 0.068, and CFI of 0.94. This confirmed one portion of the exploratory ABCD Study analysis (also a bifactor model), as well as allowed us to generate orthogonal scores from the PNC model, including a general exposome (*Exp-factor*) score (as done in the ABCD sample). Notably, the generation of a PNC-derived *Exp-factor* allowed us to test associations with mental and general health outcomes in an attempt to replicate findings from the ABCD Study, despite the PNC having much “leaner” characterization of environment compared with the ABCD Study (*n* = 29 variables in PNC compared with *n* = 348 variables in ABCD Study, with no data on school and family dynamics in the PNC).

As seen in Figure S3, factor analysis of all 29 environmental variables defined four factors (*household adversity*, *neighborhood environment*, *trauma exposure*, and *early life*). Table S28 provides a full description of the four-factor solution and details the environmental exposures in the PNC and their loadings on the exposome factors obtained from the CFA.

#### Association of exposome factor scores with psychopathology, obesity, and pubertal development in PNC

**Figure 5.**
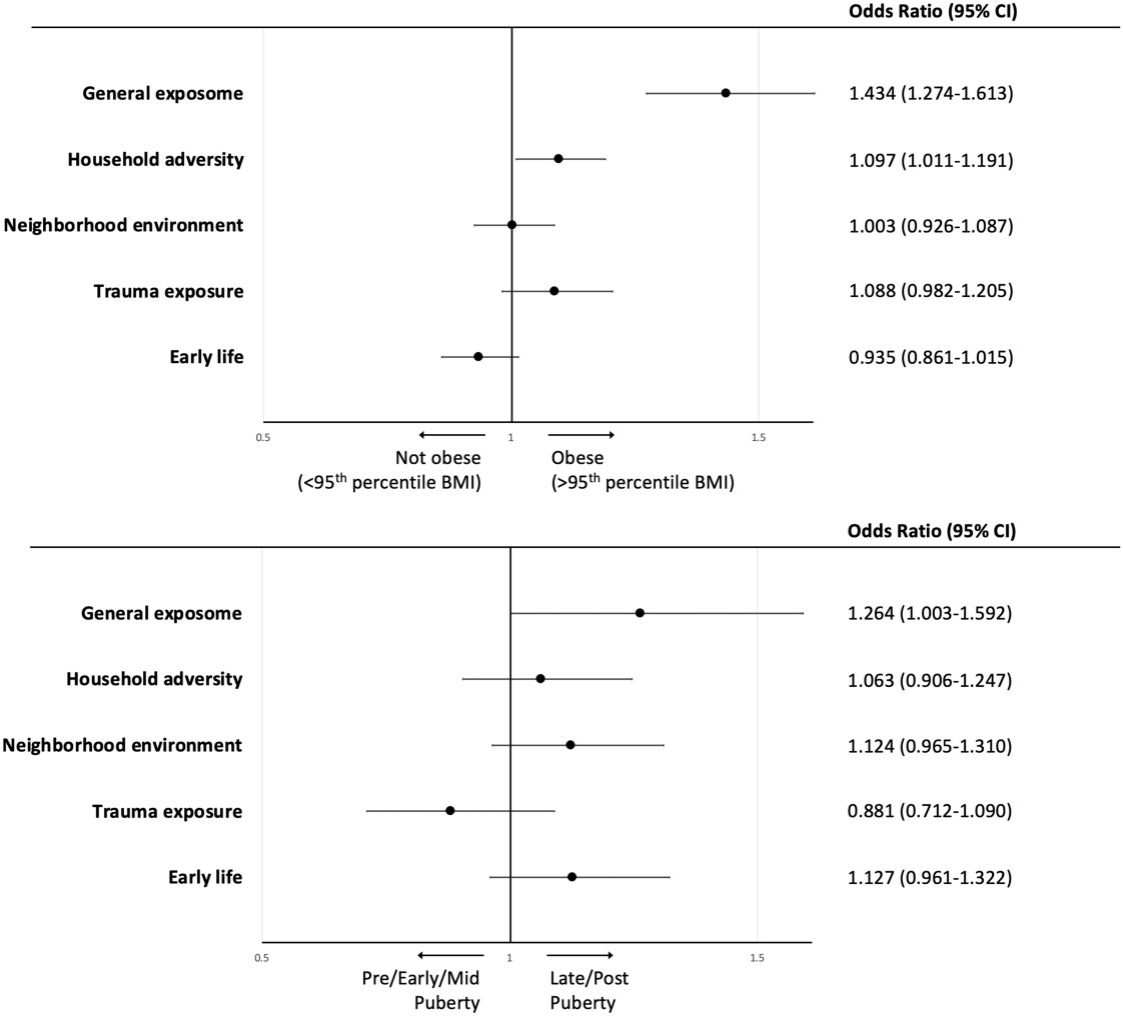
Association of exposome factor scores with obesity and pubertal development in the PNC. Association of the PNC exposome factor scores with obesity (binary variable, BMI ≥ 95th percentile, top) and late or post-pubertal stage (binary variable, contrasted against pre-, early, and mid-pubertal stage, bottom). ORs were extracted from a binary logistic regression model with exposome factor scores as independent variables, co-varying for age, sex, race (White, Black, and other), ethnicity (Hispanic), and parent education. Puberty model also co-varies for BMI. For models testing associations with pubertal measures, the PNC sample was limited to age range 10-12 to minimize age effects on models. Sample included *N* = 1496, of whom 271 were at late/post-pubertal status.

Consistent with the findings from ABCD Study exposome analyses, we found that the addition of the exposome factors substantially increased the variance explained (adjusted *R*^2^) in *P-factor* score, from <4% (when relying on demographics alone) to 18.4%, with the *Exp-factor* similarly associated with *P-factor* score, though with a smaller effect size than in the ABCD Study (*B* = 0.15, 95% CI, 0.26-0.3, *P* < .001 in PNC vs *B* = 0.285 in ABCD Study; see Table 3 for full model statistics). Similar to analyses in the ABCD Study, the *Exp-factor* was significantly associated with obesity (OR, 1.43; 95% CI, 1.27-1.61, *P* < .001 in PNC vs OR, 1.41 in ABCD Study) and advanced pubertal development (4/5 on a 5-point Likert scale; OR, 1.26; 95% CI, 1-1.59, *P* = .047 in PNC vs OR, 1.3 in ABCD Study; Figure 5 and Tables S29 and S30).

**Table 3.** Association of exposome factor scores with psychopathology (*P-factor* score) in PNC

	Model 1 (Demographics)			Model 2 (Demographics + Exposome)		
	Beta	SE	*P*	Beta	SE	*P*
Age (months)	0.029	0.02	.05	**−**0.024	0.02	.085
Female sex	**−**0.038	0.019	.01	**−**0.025	0.013	.064
White race	**−**0.120	0.026	<.001	**−**0.067	0.024	.006
Black race	0.009	0.028	.736	**−**0.012	0.025	.640
Hispanic ethnicity	**−**0.021	0.017	.217	**−**0.009	0.016	.575
Parent education (years)	**−**0.108	0.015	<.001	**−**0.046	0.015	.003
General exposome adversity				0.150	0.020	<.001
Household adversity				0.139	0.014	<.001
Neighborhood environment				0.025	0.014	.072
Trauma exposure				0.314	0.018	<.001
Early life				0.091	0.014	<.001
Adjusted *R*^2^	0.039			0.184		

Effect sizes (standardized betas) derived from a linear regression model testing association of demographics and exposome factors with general psychopathology (*P-factor*).

#### “Harmonized” models across ABCD Study and PNC

To maximize “harmonization” across the ABCD Study and PNC datasets, we tested the associations of exposome factors with *P-factor* score, BMI, depression, ADHD, obesity, and pubertal development including identical covariates available in both the ABCD Study and PNC (age, sex, race, ethnicity, and parental education). These analyses showed consistency across both youth cohorts (Table 4).

**Table 4.** Association of the general exposome factor score with health measures in ABCD Study and PNC using identical covariates in both cohorts (age, sex, race, ethnicity, and parental education)

Cohort	Dependent variable	Standardized beta	95% CI	*P*
ABCD	*P-factor*	0.28	0.26-3	<.001
PNC	*P-factor*	0.15	0.11-0.19	<.001
ABCD	BMI	0.16	0.14-0.18	<.001
PNC	BMI	0.15	0.1-0.19	<.001

**Cohort**	**Dependent variable**	**OR**	**95% CI**	***P***

ABCD	Depression	1.61	1.44-1.80	<.001
PNC	Depression	1.28	1.06-1.46	.012
ABCD	ADHD	1.35	1.27-1.43	<.001
PNC	ADHD	1.17	1.05-1.3	.005
ABCD	Obesity	1.43	1.34-1.53	<.001
PNC	Obesity	1.43	1.27-1.61	<.001
ABCD	Late/post-pubertal	1.37	1.23-1.52	<.001
PNC^a^	Late/post-pubertal	1.26	1-1.59	.047

Effect sizes derived from a linear regression model (standardized betas for continuous and OR for binary measures) testing associations of the general exposome factor score with various health and mental health variables.

a For the models in the PNC testing associations with pubertal development status we included participants ages 10-12 (*n* = 1496).

## Discussion

Here, we provide a comprehensive investigation of the exposome with a focus on psychosocial environment in early adolescence in the United States in two separate large youth samples. We show that a data-driven approach allows calculation of exposome factors that capture the shared variance among multi-level environmental exposures, and that these exposome factors explain substantial variance in early adolescent general and mental health. Our approach using bifactor modeling of the exposome revealed a general exposome adversity factor score that was obtained independently in two separate cohorts, even though one cohort provided substantially more detailed environmental data than the other (*n* = 348 exposures in ABCD Study and *n* = 29 exposures in PNC). Our work adds to previous analyses that focus on individual correlated exposome subfactors in ABCD Study,^19^ suggesting that general exposome score can be generated and is useful to capture environment’s role in explaining variance in health outcomes among youth. While the current study analyzed cross-sectional data and cannot be used to infer causality, we suggest that our work provides a roadmap for dissection of environmental effects on developmental outcomes that accounts for the exposome’s complexity.

This research is important for several reasons. *First*, it demonstrates how inevitably collinear environmental exposures can be modeled when they are captured at multiple levels. For example, the *household adversity* subfactor in the ABCD Study had strong loadings on youth-report of parental trouble with the law, parental self-reported psychopathology, developmental history (capturing prenatal exposure to cannabis), and parent-report of poverty and whether pregnancy was planned. Therefore, when trying to dissect associations of specific exposures with developmental outcomes based on a priori knowledge and hypotheses, one should account for the collinearity that is likely to confound any relationship that a specific exposure may have with an index outcome of choice. *Second*, our results suggest that data-driven approaches to characterizing the exposome may be important to reveal latent factors that cannot be identified with a priori knowledge. A key example is the prenatal exposure items in the ABCD Study, from which items split between the *household adversity* subfactor (prenatal exposure to substances, planned pregnancy) and the *pregnancy/birth complications* subfactor. Notably, growing efforts try to link pre-/post-natal exposures in the ABCD Study to developmental outcomes (prenatal cannabis exposure,^53^ breastfeeding,^54^ and other prenatal adversities^55^). Hence, it will become increasingly important to rigorously account for exposome complexity to allow generalizability and replicability of findings and identify causal mechanisms that are not confounded by collinear exposures. *Third*, in the context of understanding variance in psychopathology, our findings provide compelling evidence for the critical need to include environmental exposures when modeling psychopathology outcomes. We observed ∼5- to 10-fold increase in *R*^2^ explaining dimensional psychopathology upon addition of exposome factors in two independent cohorts, over and above the commonly used estimators of socioeconomic environment (parental education and household income). Of note, while we could not test for causality in this work, we suggest that the inclusion of exposome factors in predictive models of psychopathology (where causality is not the focus) may improve their performance considerably. *Fourth*, our finding on exposome contribution to variance in obesity and pubertal development in two independent samples provides a proof-of-concept for the utility of studying exposome effects on health trajectories in youth as they mature. *Fifth*, our ability to generalize the exposome framework and show that a general exposome factor score can be calculated in an independent youth sample that is different in both its demographic characteristic and its much leaner environmental phenotyping may suggest that our findings have implications for modeling environmental effects in other developmental cohorts in the United States and globally.

We suggest that this study be considered a roadmap when modeling environment in future investigations of developmental trajectories in longitudinal cohort studies. Notably, the current study does not investigate the exposome’s associations with cognitive and imaging measures, which could be done in future works utilizing multimodal and longitudinal datasets. Additionally, the orthogonal exposome subfactors can be used to explore interactions within the exposome (environment-by-environment interaction), which have been identified in association with baseline ABCD Study cognitive and imaging outcomes.^56^ Similarly, the general exposome factor can be used as a covariate to adjust for nuisance environmental variance in studies with smaller samples or when trying to dissect the link between a specific exposure and an outcome. Moreover, we suggest that integration of genetic data with the general exposome factor can facilitate better modeling when studying gene-by-environment interaction mechanisms in developmental cohorts, allowing researchers to reliably measure environment (with all its complexities) as a dimensional construct in conjunction with polygenic risk scores as dimensional genetic burden,^57^ as recently shown in an adult cohort.^58^ Lastly, our findings in the ABCD Study reveal large quantitative differences in latent environmental factors that illuminate disparities among demographic groups in America, which likely relate to disparities in later-life health outcomes.^59^ We suggest that the exposome factors be used to identify and focus on high-risk subgroups in large population cohorts that are more difficult to identify using a priori knowledge. Studies of such subpopulations are critical in the effort to tease apart mechanisms of resilience, which are themselves influenced by multiple dimensions of environment^60^ (ie, intrapersonal, family, and neighborhood), and therefore require investigation in a wide environmental context.

Among the six exposome subfactors identified in ABCD Study, we found the day-to-day experiences subfactor was the one most strongly associated with psychopathology and the only subfactor associated with advanced pubertal status. We suggest that these findings highlight the critical toll that day-to-day stressors take on mental health, and the potential impact of these stressors on youth’s allostatic load.^61^ It is possible that the association of day-to-day adverse psychosocial exposome with advanced pubertal status is an indication of increased allostatic load manifested by accelerated aging (ie, advanced pubertal development). Indeed, most items that loaded into this exposome subfactor included experiences related to day-to-day close psychosocial environment at school (like sense of involvement, receiving school feedback), parents (acceptance by parent, family conflict), and peer stress (mostly experiences of discrimination). Relatedly, in a hypothesis-driven study, we recently described the association between discrimination stress and pubertal development in ABCD Study, including association of such experiences with higher pubertal hormone levels in girls.^62^

A few methodological considerations we took are worth discussion. First, when selecting environmental variables to include in analysis, we generally tried to take an inclusive approach informed by literature on environmental effects on development.^2^^,^^63^ We included some variables that have substantial genetic components (eg, parent psychopathology) and others that may be confounded by psychopathology or subjective experience (eg, school enjoyment). We chose not to include substance use variables, which we considered to reflect “psychopathology indicators” rather than environmental exposures in the young age range of this study. Second, while we refer to our modeling of environment as “exposome,” we acknowledge that most of the exposures we included in this exposomic analysis were self- or parent-report using survey data tapping into psychosocial environment. As we relied on secondary analyses of data, we could only include exposures that were collected in the ABCD Study and PNC, and these studies collected limited data on physical exposures on the neighborhood level, and no data on physical exposures at the individual level. Third, we chose to use a bifactor model to fit the exposome data. This was largely in anticipation of a general exposome factor that would “absorb” any correlations among the latent factors. This model also produces orthogonal scores useful in downstream analyses to interpret specific effects. Further description of the rationale behind these decisions is detailed in full in “Methods” (variable selection) and Supplemental Methods.

### Limitations

Our findings should be viewed considering several limitations. First, we acknowledge that although we attempted to include all possible environmental factors in the two datasets, we nevertheless had to follow a reasoned decision-making process to determine what exactly to include in our analyses. For example, in the ABCD Study, we used composite scores as opposed to raw scores in some instances; and in the PNC, we chose to include specific geocoded census-derived variables based on our previous works. These decisions could have influenced results. Nevertheless, the current analysis provides, to our knowledge, the most comprehensive evaluation of environment in developmental cohorts and includes youth-report of key adversities that have not been included in previous studies. Second, many of the exposures included in the current analysis are based on self-report. This may inflate some of the effects we observed in psychopathology that is also based on self-report. This potential inflation in effects can explain why day-to-day self-reported experiences showed greatest associations with psychopathology. For example, a depressed youth will be more likely to report negative environmental exposures. Still, we showed substantial associations of exposome with psychopathology when using parent-report measure of psychopathology, and we show that exposome substantially explains pubertal development and obesity, which are not confounded by self-report. Third, we used cross-sectional data to test associations of the exposome factors with psychopathology, obesity, and pubertal development. Longitudinal studies are warranted to evaluate temporal relationships between the exposome and health trajectories and identify causal mechanisms. Fourth, our study does not address the complexity of genetic contributions to environmental exposures (including gene–environment correlations). This line of research is critical to address specificity of exposome effects on development and merits thorough future investigation outside the scope of the current work. Fifth, the CFI < 0.90 for the ABCD exposome model warrants interpretation of results with some caution, although the specific interpretation of inconsistent fit indices (here, acceptable SRMR and RMSEA, with CFI < 0.90) is still not well established. Importantly, inconsistency between RMSEA and CFI does not necessarily indicate that the model is misspecified that the data have flaws.^47^ Sixth, while the PNC was similar to the ABCD Study sample in terms of mean age and gender distribution, it was significantly different in its racial/ethnic composition and had significantly fewer environmental exposures for replication. Relatedly, each dataset had its inherent limitations. PNC data were collected at one site, making it impossible to address state-level environment. In contrast, a sample as complex as the ABCD Study includes much potential for measurement invariance violations—by race, by sex, by site, and other demographic groupings for example. It is important for future research to investigate consistency of measurement models across groups and sites, but it is beyond the scope of the current work. Finally, we did not take a “best practice” approach to the factor analyses (ie, split the sample, estimate an EFA model in one portion, and test the EFA model in a CFA in the other portion). However, we did not intend to test a theoretical structural model, not even the one “found” by the EFA. Instead, the purpose was to derive scores from the model that most reasonably fit the entire ABCD Study and PNC datasets. We anticipate that cross-validation of the scores will occur as they are used in downstream analyses, especially of longitudinal data that are and will be available for both cohorts.

## Conclusion

We leveraged two large, diverse datasets of US adolescents with deep phenotyping of environmental exposures to produce a roadmap for studying the exposome in youth. We propose that the exposome paradigm allows research to move beyond “looking under the lamp post” to a rounded dimensional investigation of environmental burden during development. We hope that future studies will build on the exposome framework in longitudinal cohorts to better understand developmental trajectories of youth through its integration in multi-omic research of brain, behavior, and health.

## Supplementary Material

osac010_Supplementary_Data

## Data Availability

Data used in the preparation of this article were obtained from the Adolescent Brain Cognitive Development Study (https://abcdstudy.org), held in the National Institute of Mental Health Data Archive. Data preprocessing and analysis are detailed at https://github.com/barzilab1/abcd_exposome.
