# Supplementary material for: Modeling environment through a general exposome factor in two independent adolescent cohorts
Source: Exposome. 2022 Dec 14;2(1):osac010. doi: 10.1093/exposome/osac010 (PMC9798749; doi:10.1093/exposome/osac010)
Supplement: osac010_Supplementary_Data [file osac010_supplementary_data.docx]

**Supplemental Material**

**Modeling Environment Through a General Exposome Factor in Two Independent Adolescent Cohorts**

Tyler M. Moore ^a,b^, Elina Visoki ^b^, Stirling T. Argabright ^b^, Grace E. DiDomenico ^b^,

Ingrid Sotelo ^b^, Jeremy D. Wortzel ^b^, Areebah Naeem ^b^, Ruben C. Gur ^a,b^, Raquel E. Gur ^a,b^, Varun Warrier ^c^, Sinan Guloksuz ^d,e^, Ran Barzilay ^a,b,f,*^

^a^ Department of Psychiatry, Perelman School of Medicine, University of Pennsylvania, Philadelphia, USA

^b^ Lifespan Brain Institute of the Children’s Hospital of Philadelphia (CHOP) and Penn Medicine, Philadelphia, USA

^c^ Autism Research Centre, Department of Psychiatry, University of Cambridge, Cambridgeshire, UK

^d^ Department of Psychiatry, Yale University School of Medicine, New Haven CT, USA

^e^ Department of Psychiatry and Neuropsychology, School for Mental Health and Neuroscience, Maastricht University Medical Centre, Maastricht, The Netherlands

^f^ Department of Child and Adolescent Psychiatry and Behavioral Science, CHOP, Philadelphia, USA

*Correspondence concerning this article should be addressed to Ran Barzilay, Perelman School of Medicine, University of Pennsylvania, 3400 Spruce St. – 10^th^ Floor Gates Pavilion, Philadelphia, PA 19104. E-mail: [ran.barzilay@pennmedicine.upenn.edu](mailto:ran.barzilay@pennmedicine.upenn.edu)

**Table of Contents**

**Supplemental Tables**

**Supplemental Table 1.** Demographic characteristics of ABCD Study sample

**Supplemental Table 2.** Comparison of ABCD Study and PNC datasets

**Supplemental Table 3.** Full set of exposome measures included in ABCD Study analysis

**Supplemental Table 4.** Full set of psychopathology measures included in ABCD Study analysis

**Supplemental Table 5.** Parental mental health and drug use in ABCD Study

**Supplemental Table 6.** Maternal drug use during pregnancy in ABCD Study

**Supplemental Table 7.** Geocoded data-derived neighborhood-level characteristics in ABCD Study

**Supplemental Table 8.** Parent-reported household-/family-level poverty and religiosity in ABCD Study

**Supplemental Table 9.** Youth-reported school-level characteristics in ABCD Study

**Supplemental Table 10.** Pregnancy complications in ABCD Study

**Supplemental Table 11.** Birth complications in ABCD Study

**Supplemental Table 12.** Parent-reported traumatic events experienced by child in ABCD Study

**Supplemental Table 13.** Youth-reported life events in ABCD Study

**Supplemental Table 14.** Bifactor indices for the exposome models (ABCD Study and PNC)

**Supplemental Table 15.** Exploratory factor analysis of optimized collection of ABCD psychopathology variables using iterated target rotation

**Supplemental Table 16.** Final confirmatory bifactor analysis of optimized collection of ABCD psychopathology variables

**Supplemental Table 17.** Final confirmatory bifactor analysis of optimized collection of ABCD exposome variables

**Supplemental Table 18.** Association of ABCD exposome factor scores with obesity

**Supplemental Table 19.** Association of ABCD exposome factor scores with late/post-pubertal status

**Supplemental Table 20.** Association of ABCD exposome factor scores with parent-reported child psychopathology

**Supplemental Table 21.** Association of ABCD exposome factor scores with depression and ADHD

**Supplemental Table 22.** Association of ABCD exposome factor scores with BMI

**Supplemental Table 23.** Association of ABCD exposome factor scores with pubertal development

**Supplemental Table 24.** Association of ABCD exposome factor scores with the P-factor accounting for site and family relatedness

**Supplemental Table 25.** Association of ABCD exposome factor scores with the CBCL accounting for site and family relatedness

**Supplemental Table 26.** Association of ABCD exposome factor scores with BMI accounting for site and family relatedness

**Supplemental Table 27.** Association of ABCD exposome factor scores with pubertal development in accounting for site and family relatedness

**Supplemental Table 28.** Final confirmatory bifactor analysis of PNC exposome variables

**Supplemental Table 29.** Association of PNC exposome factor scores with obesity

**Supplemental Table 30.** Association of PNC exposome factor scores with pubertal development

**Supplemental Figures**

**Supplemental Figure 1.** Schematic illustration of dimensionality reduction pipeline of environmental exposures in ABCD Study

**Supplemental Figure 2.** ABCD psychopathology bifactor model

**Supplemental Figure 3.** PNC exposome bifactor model

**Supplemental Figure 4.** Scree plot of eigenvalues of the initial exposome mixed correlation matrix of 96 environmental exposures in ABCD Study

**Supplemental Methods**

**Supplemental Table 1.** Demographic characteristics of ABCD Study sample

|  | **N/mean** | **%/SD** |
| --- | --- | --- |
| **Sex (F)** | 5356 | 47.7% |
| **Age (years)** | 10.93 | 0.64 |
| **Race** |  |  |
| White | 8453 | 75.2% |
| Black | 2269 | 20.2% |
| Asian | 723 | 6.4% |
| Native American | 386 | 3.4% |
| Native Hawaiian/Pacific Islander | 70 | 0.6% |
| **Hispanic** | 2226 | 20.1% |
| **Household income** |  |  |
| <$5,000 | 347 | 3.3% |
| $5,000 - $11,999 | 336 | 3.2% |
| $12,000 - $15,999 | 241 | 2.3% |
| $16,000 - $24,999 | 446 | 4.3% |
| $25,000 - $34,999 | 604 | 5.8% |
| $35,000 - $49,999 | 838 | 8.1% |
| $50,000 - $74,999 | 1363 | 13.2% |
| $75,000 - $99,999 | 1460 | 14.1% |
| $100,000 - $199,999 | 3371 | 32.5% |
| >$200,000 | 1358 | 13.1% |
| **Parent education (years)** | 16.51 | 2.63 |

Basic demographic information is presented for the 11,235 participants that make up the ABCD Study population, including age, sex, race, ethnicity, household income, and parents’ education.

**Supplemental Table 2.** Comparison of ABCD Study and PNC datasets

|  | **ABCD Sample** (N=11,235) | | **PNC replication sample** (N=4,993) | |
| --- | --- | --- | --- | --- |
|  | **N/mean** | **%/SD** | **N/mean** | **%/SD** |
| **Sex (F)** | 5,356 | 47.70% | 2,137 | 47.60% |
| **Age (years)** | 10.93 | 0.64 | 10.89 | 1.75 |
| **White race** | 8,453 | 75.20% | 2,521 | 56.10% |
| **Black race** | 2,269 | 20.20% | 1,419 | 31.60% |
| **Hispanic ethnicity** | 2,226 | 20.10% | 329 | 7.30% |
| **Parent education (years) [average of both parents in ABCD Study,**  **only maternal education in PNC due to missing data in fathers]** | 16.51 | 2.63 | 14.63 | 2.46 |
| **Self-report of trauma & internalizing symptoms** | 11,235 | 100% | 2,135 | 47.50% |

A demographic comparison of the ABCD Study sample and the PNC replication dataset.

**Supplemental Table 3.** Full set of exposome measures included in ABCD Study analysis

| **ABCD instrument** | **ABCD item code** | **ABCD item description** | **ABCD subinstrument code** | **ABCD subitem code** | **ABCD subitem description** | **Reporter** | **Time point** |
| --- | --- | --- | --- | --- | --- | --- | --- |
| ABCD Longitudinal Summary Scores Sports Activity (abcd_lsssa01) | sai_ss_dance_nmonth_p_l | Months per year ballet/dance (past year) | abcd_lpsaiq01 | sai_p_activities_l___0 | Ballet, Dance | Parent | 1-year follow-up |
|  |  |  |  | sai_p_dance_nmonth_l | Since we last saw you, about how many months did your child participate in this activity? | Parent | 1-year follow-up |
|  | sai_ss_base_nmonth_p_l | Months per year baseball/softball (past year) |  | sai_p_activities_l___1 | Baseball, Softball | Parent | 1-year follow-up |
|  |  |  |  | sai_p_base_nmonth_l | Since we last saw you, about how many months did your child participate in this activity? | Parent | 1-year follow-up |
|  | sai_ss_basket_nmonth_p_l | Months per year basketball (past year) |  | sai_p_activities_l___2 | Basketball | Parent | 1-year follow-up |
|  |  |  |  | sai_p_basket_nmonth_l | Since we last saw you, about how many months did your child participate in this activity? | Parent | 1-year follow-up |
|  | sai_ss_climb_nmonth_p_l | Months per year climbing (past year) |  | sai_p_activities_l___3 | Climbing | Parent | 1-year follow-up |
|  |  |  |  | sai_p_climb_nmonth_l | Since we last saw you, about how many months did your child participate in this activity? | Parent | 1-year follow-up |
|  | sai_ss_fhock_nmonth_p_l | Months per year field hockey (past year) |  | sai_p_activities_l___4 | Field hockey | Parent | 1-year follow-up |
|  |  |  |  | sai_p_fhock_nmonth_l | Since we last saw you, about how many months did your child participate in this activity? | Parent | 1-year follow-up |
|  | sai_ss_fball_nmonth_p_l | Months per year football (past year) |  | sai_p_activities_l___5 | Football | Parent | 1-year follow-up |
|  |  |  |  | sai_p_fball_nmonth_l | Since we last saw you, about how many months did your child participate in this activity? | Parent | 1-year follow-up |
|  | sai_ss_gym_nmonth_p_l | Months per year gymnastics (past year) |  | sai_p_activities_l___6 | Gymnastics | Parent | 1-year follow-up |
|  |  |  |  | sai_p_gym_nmonth_l | Since we last saw you, about how many months did your child participate in this activity? | Parent | 1-year follow-up |
|  | sai_ss_ihock_nmonth_p_l | Months per year ice hockey (past year) |  | sai_p_activities_l___7 | Ice Hockey | Parent | 1-year follow-up |
|  |  |  |  | sai_p_ihock_nmonth_l | Since we last saw you, about how many months did your child participate in this activity? | Parent | 1-year follow-up |
|  | sai_ss_polo_nmonth_p_l | Months per year horseback riding/polo (past year) |  | sai_p_activities_l___8 | Horseback Riding, Polo | Parent | 1-year follow-up |
|  |  |  |  | sai_p_polo_nmonth_l | Since we last saw you, about how many months did your child participate in this activity? | Parent | 1-year follow-up |
|  | sai_ss_iskate_nmonth_p_l | Months per year ice/inline skating (past year) |  | sai_p_activities_l___9 | Ice or Inline Skating | Parent | 1-year follow-up |
|  |  |  |  | sai_p_iskate_nmonth_l | Since we last saw you, about how many months did your child participate in this activity? | Parent | 1-year follow-up |
|  | sai_ss_m_arts_nmonth_p_l | Months per year martial arts (past year) |  | sai_p_activities_l___10 | Martial Arts | Parent | 1-year follow-up |
|  |  |  |  | sai_p_m_arts_nmonth_l | Since we last saw you, about how many months did your child participate in this activity? | Parent | 1-year follow-up |
| ABCD Longitudinal Summary Scores Sports Activity (abcd_lsssa01) | sai_ss_lax_nmonth_p_l | Months per year lacrosse (past year) | abcd_lpsaiq01 | sai_p_activities_l___11 | Lacrosse | Parent | 1-year follow-up |
|  |  |  |  | sai_p_lax_nmonth_l | Since we last saw you, about how many months did your child participate in this activity? | Parent | 1-year follow-up |
|  | sai_ss_rugby_nmonth_p_l | Months per year rugby (past year) |  | sai_p_activities_l___12 | Rugby | Parent | 1-year follow-up |
|  |  |  |  | sai_p_rugby_nmonth_l | Since we last saw you, about how many months did your child participate in this activity? | Parent | 1-year follow-up |
|  | sai_ss_skate_nmonth_p_l | Months per year skateboarding (past year) |  | sai_p_activities_l___13 | Skateboarding | Parent | 1-year follow-up |
|  |  |  |  | sai_p_skate_nmonth_l | Since we last saw you, about how many months did your child participate in this activity? | Parent | 1-year follow-up |
|  | sai_ss_sboard_nmonth_p_l | Months per year skiing/snowboarding (past year) |  | sai_p_activities_l___14 | Skiing, Snowboarding | Parent | 1-year follow-up |
|  |  |  |  | sai_p_sboard_nmonth_l | Since we last saw you, about how many months did your child participate in this activity? | Parent | 1-year follow-up |
|  | sai_ss_soc_nmonth_p_l | Months per year soccer (past year) |  | sai_p_activities_l___15 | Soccer | Parent | 1-year follow-up |
|  |  |  |  | sai_p_soc_nmonth_l | Since we last saw you, about how many months did your child participate in this activity? | Parent | 1-year follow-up |
|  | sai_ss_surf_nmonth_p_l | Months per year surfing (past year) |  | sai_p_activities_l___16 | Surfing | Parent | 1-year follow-up |
|  |  |  |  | sai_p_surf_nmonth_l | Since we last saw you, about how many months did your child participate in this activity? | Parent | 1-year follow-up |
|  | sai_ss_wpolo_nmonth_p_l | Months per year swimming/water polo (past year) |  | sai_p_activities_l___17 | Swimming, Water Polo | Parent | 1-year follow-up |
|  |  |  |  | sai_p_wpolo_nmonth_l | Since we last saw you, about how many months did your child participate in this activity? | Parent | 1-year follow-up |
|  | sai_ss_tennis_nmonth_p_l | Months per year tennis (past year) |  | sai_p_activities_l___18 | Tennis | Parent | 1-year follow-up |
|  |  |  |  | sai_p_tennis_nmonth_l | Since we last saw you, about how many months did your child participate in this activity? | Parent | 1-year follow-up |
|  | sai_ss_run_nmonth_p_l | Months per year track/running/cross-country (past year) |  | sai_p_activities_l___19 | Track, Running, Cross-country | Parent | 1-year follow-up |
|  |  |  |  | sai_p_run_nmonth_l | Since we last saw you, about how many months did your child participate in this activity? | Parent | 1-year follow-up |
|  | sai_ss_mma_nmonth_p_l | Months per year wrestling/mixed martial arts (past year) |  | sai_p_activities_l___20 | Wrestling, Mixed Martial Arts | Parent | 1-year follow-up |
|  |  |  |  | sai_p_mma_nmonth_l | Since we last saw you, about how many months did your child participate in this activity? | Parent | 1-year follow-up |
|  | sai_ss_vball_nmonth_p_l | Months per year volleyball (past year) |  | sai_p_activities_l___21 | Volleyball | Parent | 1-year follow-up |
|  |  |  |  | sai_p_vball_nmonth_l | Since we last saw you, about how many months did your child participate in this activity? | Parent | 1-year follow-up |
|  | sai_ss_yoga_nmonth_p_l | Months per year yoga/tai chi (past year) |  | sai_p_activities_l___22 | Yoga, Tai Chi | Parent | 1-year follow-up |
|  |  |  |  | sai_p_yoga_nmonth_l | Since we last saw you, about how many months did your child participate in this activity? | Parent | 1-year follow-up |
|  | sai_ss_music_nmonth_p_l | Months per year playing musical instrument (singing/choir/guitar/piano/drums/violin/flute/band/rock band/orchestra) (past year) |  | sai_p_activities_l___23 | Musical Instrument (Singing, Choir, Guitar, Piano, Drums, Violin, Flute, Band, Rock Band, Orchestra) | Parent | 1-year follow-up |
| ABCD Longitudinal Summary Scores Sports Activity (abcd_lsssa01) | sai_ss_music_nmonth_p_l | Months per year playing musical instrument (singing/choir/guitar/piano/drums/violin/flute/band/rock band/orchestra) (past year) | abcd_lpsaiq01 | sai_p_music_instr___0 | Guitar, Bass Guitar, Ukulele | Parent | 1-year follow-up |
|  |  |  |  | sai_p_music_instr___1 | Flute, Piccolo, Recorder | Parent | 1-year follow-up |
|  |  |  |  | sai_p_music_instr___2 | Clarinet, Saxophone, Oboe, Bassoon | Parent | 1-year follow-up |
|  |  |  |  | sai_p_music_instr___3 | Violin, Viola | Parent | 1-year follow-up |
|  |  |  |  | sai_p_music_instr___4 | Cello, Bass | Parent | 1-year follow-up |
|  |  |  |  | sai_p_music_instr___5 | Piano | Parent | 1-year follow-up |
|  |  |  |  | sai_p_music_instr___6 | Harp | Parent | 1-year follow-up |
|  |  |  |  | sai_p_music_instr___7 | Drums, Percussion | Parent | 1-year follow-up |
|  |  |  |  | sai_p_music_instr___8 | Trumpet, Trombone, Horns | Parent | 1-year follow-up |
|  |  |  |  | sai_p_music_instr___9 | Vocal, Singing | Parent | 1-year follow-up |
|  |  |  |  | sai_p_music_instr___10 | Electric Keyboard, Organ | Parent | 1-year follow-up |
|  |  |  |  | sai_p_music_instr___11 | DJ, Electronic Dance Music | Parent | 1-year follow-up |
|  |  |  |  | sai_p_music_instr___12 | Other | Parent | 1-year follow-up |
|  |  |  |  | sai_p_music_nmonth_l | Since we last saw you, about how many months did your child participate in this activity? | Parent | 1-year follow-up |
|  | sai_ss_art_nmonth_p_l | Months per year drawing/painting/graphic art/photography/pottery/sculpting (past year) |  | sai_p_activities_l___24 | Drawing, Painting, Graphic Art, Photography, Pottery, Sculpting | Parent | 1-year follow-up |
|  |  |  |  | sai_p_art_nmonth_l | Since we last saw you, about how many months did your child participate in this activity? | Parent | 1-year follow-up |
|  | sai_ss_drama_nmonth_p_l | Months per year drama/theater/acting/film (past year) |  | sai_p_activities_l___25 | Drama, Theater, Acting, Film | Parent | 1-year follow-up |
|  |  |  |  | sai_p_drama_nmonth_l | Since we last saw you, about how many months did your child participate in this activity? | Parent | 1-year follow-up |
|  | sai_ss_crafts_nmonth_p_l | Months per year doing crafts like knitting/building model cars or airplanes (past year) |  | sai_p_activities_l___26 | Crafts like Knitting, Building Model Cars or Airplanes | Parent | 1-year follow-up |
|  |  |  |  | sai_p_crafts_nmonth_l | Since we last saw you, about how many months did your child participate in this activity? | Parent | 1-year follow-up |
|  | sai_ss_chess_nmonth_p_l | Months per year playing competitive games like chess/cards/darts (past year) |  | sai_p_activities_l___27 | Competitive Games like Chess, Cards, or Darts | Parent | 1-year follow-up |
|  |  |  |  | sai_p_chess_nmonth_l | Since we last saw you, about how many months did your child participate in this activity? | Parent | 1-year follow-up |
|  | sai_ss_collect_nmonth_p_l | Months per year participating in hobbies like collecting stamps or coins (past year) |  | sai_p_activities_l___28 | Hobbies like collecting stamps or coins | Parent | 1-year follow-up |
|  |  |  |  | sai_p_collect_nmonth_l | Since we last saw you, about how many months did your child participate in this activity? | Parent | 1-year follow-up |
| ABCD Sum Scores Culture & Environment Parent (abcd_sscep01) | nsc_p_ss_mean_3_items | Neighborhood Safety Protocol: mean of parent report: (neighborhood1r_p + neighborhood2r_p + neighborhood3r_p)/3 | abcd_pnsc01 | neighborhood1r_p | "I feel safe walking in my neighborhood, day or night" | Parent | 1-year follow-up |
| ABCD Sum Scores Culture & Environment Parent (abcd_sscep01) | nsc_p_ss_mean_3_items | Neighborhood Safety Protocol: mean of parent report: (neighborhood1r_p + neighborhood2r_p + neighborhood3r_p)/3 | abcd_pnsc01 | neighborhood2r_p | "Violence is not a problem in my neighborhood" | Parent | 1-year follow-up |
|  |  |  |  | neighborhood3r_p | "My neighborhood is safe from crime" | Parent | 1-year follow-up |
|  | fes_p_ss_fc | Conflict subscale from the Family Environment Scale: sum of parent report (raw score): fam_enviro1_p + fam_enviro2r_p + fam_enviro3_p + fam_enviro4r_p + fam_enviro5_p + fam_enviro6_p + fam_enviro7r_p + fam_enviro8_p + fam_enviro9r_p | fes02 | fam_enviro1_p | "We fight a lot in our family" | Parent | 1-year follow-up |
|  |  |  |  | fam_enviro2_p | "Family members rarely become openly angry" | Parent | 1-year follow-up |
|  |  |  |  | fam_enviro3_p | "Family members sometimes get so angry they throw things" | Parent | 1-year follow-up |
|  |  |  |  | fam_enviro4_p | "Family members hardly ever lose their tempers" | Parent | 1-year follow-up |
|  |  |  |  | fam_enviro5_p | "Family members often criticize each other" | Parent | 1-year follow-up |
|  |  |  |  | fam_enviro6_p | "Family members sometimes hit each other" | Parent | 1-year follow-up |
|  |  |  |  | fam_enviro7_p | "If there is a disagreement in our family, we try hard to smooth things over and keep the peace" | Parent | 1-year follow-up |
|  |  |  |  | fam_enviro8_p | "Family members often try to one-up or outdo each other" | Parent | 1-year follow-up |
|  |  |  |  | fam_enviro9_p | "In our family, we believe you don't ever get anywhere by raising your voice" | Parent | 1-year follow-up |
|  | fes_p_ss_fc_pr | Conflict subscale from the Family Environment Scale: sum of parent report (prorated score: Calculation: (fes_p_ss_fc)*(fes_p_ss_fc_nt)/(fes_p_ss_fc_na) | abcd_sscep01 | fes_p_ss_fc | Conflict subscale from the Family Environment Scale: sum of parent report (raw score) | Parent | 1-year follow-up |
|  |  |  |  | fes_p_ss_fc_nt | Number of questions | Parent | 1-year follow-up |
|  |  |  |  | fes_p_ss_fc_na | Number of questions answered | Parent | 1-year follow-up |
|  | macv_p_ss_fs | MACVS Family Support subscale: mean: (mex_american2_p + mex_american7_p + mex_american12_p + mex_american16_p + mex_american21_p + mex_american26_p)/6 | macv01 | mex_american2_p | "Parents should teach their children that the family always comes first" | Parent | 1-year follow-up |
|  |  |  |  | mex_american7_p | "Family provides a sense of security because they will always be there for you" | Parent | 1-year follow-up |
|  |  |  |  | mex_american12_p | "It is always important to be united as a family" | Parent | 1-year follow-up |
|  |  |  |  | mex_american16_p | "It is important to have close relationships with aunts/uncles, grandparents, and cousins" | Parent | 1-year follow-up |
|  |  |  |  | mex_american21_p | "Holidays and celebrations are important because the whole family comes together" | Parent | 1-year follow-up |
|  |  |  |  | mex_american26_p | "It is important for family members to show their love and affection to one another" | Parent | 1-year follow-up |
|  | macv_p_ss_fo | MACVS Family Obligation subscale: mean: (mex_american3_p + mex_american8_p + mex_american13_p + mex_american17_p + mex_american22_p)/5 |  | mex_american3_p | "Children should be taught that it is their duty to care for their parents when their parents get old" | Parent | 1-year follow-up |
|  |  |  |  | mex_american8_p | "If a relative is having a hard time financially, one should help them out if possible" | Parent | 1-year follow-up |
|  |  |  |  | mex_american13_p | "A person should share their home with relatives if they need a place to stay" | Parent | 1-year follow-up |
|  |  |  |  | mex_american17_p | "Older kids should take care of and be role models for their younger brothers and sisters" | Parent | 1-year follow-up |
|  |  |  |  | mex_american22_p | "Parents should be willing to make great sacrifices to make sure their children have a better life" | Parent | 1-year follow-up |
| ABCD Sum Scores Culture & Environment Parent (abcd_sscep01) | macv_p_ss_isr | MACVS Independence & Self-Reliance subscale: mean: (mex_american5_p + mex_american10_p + mex_american14_p + mex_american19_p + mex_american24_p)/5 | macv01 | mex_american5_p | "People should learn how to take care of themselves and not depend on others" | Parent | 1-year follow-up |
|  |  |  |  | mex_american10_p | "The most important thing parents can teach their children is to be independent from others" | Parent | 1-year follow-up |
|  |  |  |  | mex_american14_p | "As children get older their parents should allow them to make their own decisions" | Parent | 1-year follow-up |
|  |  |  |  | mex_american19_p | "When there are problems in life, a person can only count on him or herself" | Parent | 1-year follow-up |
|  |  |  |  | mex_american24_p | "Parents should encourage children to solve their own problems" | Parent | 1-year follow-up |
|  | macv_p_ss_fr | MACVS Family as Referent subscale: mean: (mex_american4_p + mex_american9_p + mex_american18_p + mex_american23_p + mex_american27_p)/5 |  | mex_american4_p | "Children should always do things to make their parents happy" | Parent | 1-year follow-up |
|  |  |  |  | mex_american9_p | "When it comes to important decisions, the family should ask for advice from close relatives" | Parent | 1-year follow-up |
|  |  |  |  | mex_american18_p | "Children should be taught to always be good because they represent the family" | Parent | 1-year follow-up |
|  |  |  |  | mex_american23_p | "A person should always think about their family when making important decisions" | Parent | 1-year follow-up |
|  |  |  |  | mex_american27_p | "It is important to work hard and do one's best because this work reflects on the family" | Parent | 1-year follow-up |
|  | macv_p_ss_r | MACVS Religion subscale: mean: (mex_american1_p + mex_american6_p + mex_american11_p + mex_american15_p + mex_american20_p + mex_american25_p + mex_american28_p)/7 |  | mex_american1_p | "One's belief in God gives inner strength and meaning to life" | Parent | 1-year follow-up |
|  |  |  |  | mex_american6_p | "God is first; family is second" | Parent | 1-year follow-up |
|  |  |  |  | mex_american11_p | "Parents should teach their children how to pray" | Parent | 1-year follow-up |
|  |  |  |  | mex_american15_p | "If everything is taken away, one still has their faith in God" | Parent | 1-year follow-up |
|  |  |  |  | mex_american20_p | "It is important to thank God every day for all one has" | Parent | 1-year follow-up |
|  |  |  |  | mex_american25_p | "It is important to follow the Word of God" | Parent | 1-year follow-up |
|  |  |  |  | mex_american28_p | "Religion should be an important part of one's life" | Parent | 1-year follow-up |
|  | meim_p_ss_exp | MEIM-R Exploration subscale: mean: (meim_1_p + meim_4_p + meim_5_p)/3 | abcd_meim01 | meim_1_p | "I have spent time trying to find out more about my ethnic group, such as its history, traditions, and customs" | Parent | baseline |
|  |  |  |  | meim_4_p | "I have often done things that will help me understand my ethnic background better" | Parent | baseline |
|  |  |  |  | meim_5_p | "I have often talked to other people in order to learn more about my ethnic group" | Parent | baseline |
|  | meim_p_ss_com | MEIM-R Commitment and Attachment subscale: mean: (meim_2_p + meim_3_p + meim_6_p)/3 |  | meim_2_p | "I have a strong sense of belonging to my own ethnic group" | Parent | baseline |
|  |  |  |  | meim_3_p | "I understand pretty well what my ethnic group membership means to me" | Parent | baseline |
|  |  |  |  | meim_6_p | "I feel a strong attachment towards my own ethnic group" | Parent | baseline |
|  | meim_p_ss_total | MEIM-R overall mean: (meim_1_p + meim_2_p + meim_3_p + meim_4_p + meim_5_p + meim_6_p)/6 |  | meim_1_p | "I have spent time trying to find out more about my ethnic group, such as its history, traditions, and customs" | Parent | baseline |
| ABCD Sum Scores Culture & Environment Parent (abcd_sscep01) | meim_p_ss_total | MEIM-R overall mean: (meim_1_p + meim_2_p + meim_3_p + meim_4_p + meim_5_p + meim_6_p)/6 | abcd_meim01 | meim_2_p | "I have a strong sense of belonging to my own ethnic group" | Parent | baseline |
|  |  |  |  | meim_3_p | "I understand pretty well what my ethnic group membership means to me" | Parent | baseline |
|  |  |  |  | meim_4_p | "I have often done things that will help me understand my ethnic background better" | Parent | baseline |
|  |  |  |  | meim_5_p | "I have often talked to other people in order to learn more about my ethnic group" | Parent | baseline |
|  |  |  |  | meim_6_p | "I feel a strong attachment towards my own ethnic group" | Parent | baseline |
| ABCD Sum Scores Physical Health Parent (abcd_ssphp01) | cna_p_ss_sum | Mind Diet score: sum: cna_1_p + cna_2_p + cna_3_p + cna_4_p + cna_5_p + cna_6_p + cna_7_p + cna_8_p + cna_9_p + cna_10_p + cna_11_p + cna_12_p + cna_13_p + cna_14_p | abcd_cna01 | cna_1_p | In a typical week, does your child eat whole grains 3 or more times per day? | Parent | 1-year follow-up |
|  |  |  |  | cna_2_p | In a typical week, does your child eat green leafy vegetables 6 or more times? | Parent | 1-year follow-up |
|  |  |  |  | cna_3_p | In a typical week, does your child eat other vegetables 1 or more time per day? | Parent | 1-year follow-up |
|  |  |  |  | cna_4_p | In a typical week, does your child eat berries 2 or more times? | Parent | 1-year follow-up |
|  |  |  |  | cna_5_p | In a typical week, does your child eat red meats and meat products less than 4 times? | Parent | 1-year follow-up |
|  |  |  |  | cna_6_p | In a typical week, does your child eat fish 1 or more times? | Parent | 1-year follow-up |
|  |  |  |  | cna_7_p | In a typical week, does your child eat poultry 2 or more times? | Parent | 1-year follow-up |
|  |  |  |  | cna_8_p | In a typical week, does your child eat beans 4 or more times? | Parent | 1-year follow-up |
|  |  |  |  | cna_9_p | In a typical week, does your child eat nuts 5 or more times? | Parent | 1-year follow-up |
|  |  |  |  | cna_10_p | In a typical week, does your child eat fast food or fried food less than 1 time? | Parent | 1-year follow-up |
|  |  |  |  | cna_11_p | In a typical week, does your child eat food in which olive oil is used as the primary oil? | Parent | 1-year follow-up |
|  |  |  |  | cna_12_p | In a typical week, does your child eat less than 1 tablespoon of butter or margarine per day? | Parent | 1-year follow-up |
|  |  |  |  | cna_13_p | In a typical week, does your child eat cheese less than 1 time? | Parent | 1-year follow-up |
|  |  |  |  | cna_14_p | In a typical week, does your child eat pastries or sweets less than 5 times? | Parent | 1-year follow-up |
| ABCD Sum Scores Culture & Environment Youth (abcd_sscey01) | fes_y_ss_fc | Conflict subscale from the Family Environment Scale: sum of youth report (raw score): fes_youth_q1 + fes_youth_q2 + fes_youth_q3 + fes_youth_q4 + fes_youth_q5 + fes_youth_q6 + fes_youth_q7 + fes_youth_q8 + fes_youth_q9 | abcd_fes01 | fes_youth_q1 | "We fight a lot in our family" | Youth | 1-year follow-up |
|  |  |  |  | fes_youth_q2 | "Family members rarely become openly angry" | Youth | 1-year follow-up |
|  |  |  |  | fes_youth_q3 | "Family members sometimes get so angry they throw things" | Youth | 1-year follow-up |
|  |  |  |  | fes_youth_q4 | "Family members hardly ever lose their tempers" | Youth | 1-year follow-up |
|  |  |  |  | fes_youth_q5 | "Family members often criticize each other" | Youth | 1-year follow-up |
| ABCD Sum Scores Culture & Environment Youth (abcd_sscey01) | fes_y_ss_fc | Conflict subscale from the Family Environment Scale: sum of youth report (raw score): fes_youth_q1 + fes_youth_q2 + fes_youth_q3 + fes_youth_q4 + fes_youth_q5 + fes_youth_q6 + fes_youth_q7 + fes_youth_q8 + fes_youth_q9 | abcd_fes01 | fes_youth_q6 | "Family members sometimes hit each other" | Youth | 1-year follow-up |
|  |  |  |  | fes_youth_q7 | "If there's a disagreement in our family, we try hard to smooth things over and keep the peace" | Youth | 1-year follow-up |
|  |  |  |  | fes_youth_q8 | "Family members often try to one-up or outdo each other" | Youth | 1-year follow-up |
|  |  |  |  | fes_youth_q9 | "In our family, we believe you don't ever get anywhere by raising your voice" | Youth | 1-year follow-up |
|  | fes_y_ss_fc_pr | Conflict subscale from the Family Environment Scale: sum of youth report (prorated score): (fes_y_ss_fc)*(fes_y_ss_fc_nt)/(fes_y_ss_fc_na) | abcd_sscey01 | fes_y_ss_fc | Conflict subscale from the Family Environment Scale: sum of youth report (raw score) | Youth | 1-year follow-up |
|  |  |  |  | fes_y_ss_fc_nt | Number of questions | Youth | 1-year follow-up |
|  |  |  |  | fes_y_ss_fc_na | Number of questions answered | Youth | 1-year follow-up |
|  | crpbi_y_ss_parent | CRPBI Acceptance subscale: mean of primary caregiver report by youth: (crpbi_parent1_y + crpbi_parent2_y + crpbi_parent3_y, crpbi_parent4_y + crpbi_parent5_y)/5 | crpbi01 | crpbi_parent1_y | "First caregiver makes me feel better after talking over my worries with him/her" | Youth | 1-year follow-up |
|  |  |  |  | crpbi_parent2_y | "First caregiver smiles at me very often" | Youth | 1-year follow-up |
|  |  |  |  | crpbi_parent3_y | "First caregiver is able to make me feel better when I am upset" | Youth | 1-year follow-up |
|  |  |  |  | crpbi_parent4_y | "First caregiver believes in showing his/her love for me" | Youth | 1-year follow-up |
|  |  |  |  | crpbi_parent5_y | "First caregiver is easy to talk to" | Youth | 1-year follow-up |
|  | crpbi_y_ss_caregiver | CRPBI Acceptance subscale: mean of secondary caregiver report by youth: (crpbi_caregiver12_y + crpbi_caregiver13_y + crpbi_caregiver14_y + crpbi_caregiver15_y + crpbi_caregiver16_y)/5 |  | crpbi_parent12_y | "Second caregiver makes me feel better after talking over my worries with him/her" | Youth | 1-year follow-up |
|  |  |  |  | crpbi_parent13_y | "Second caregiver smiles at me very often" | Youth | 1-year follow-up |
|  |  |  |  | crpbi_parent14_y | "Second caregiver is able to make me feel better when I am upset" | Youth | 1-year follow-up |
|  |  |  |  | crpbi_parent15_y | "Second caregiver believes in showing his/her love for me" | Youth | 1-year follow-up |
|  |  |  |  | crpbi_parent16_y | "Second caregiver is easy to talk to" | Youth | 1-year follow-up |
|  | pmq_y_ss_mean | Parental Monitoring score: mean: (parent_monitor_q1_y + parent_monitor_q2_y + parent_monitor_q3_y + parent_monitor_q4_y parent_monitor_q5_y)/5 | pmq01 | parent_monitor_q1_y | How often do your parents/guardians know where you are? | Youth | 1-year follow-up |
|  |  |  |  | parent_monitor_q2_y | How often do your parents know who you are with when you are not at school and away from home? | Youth | 1-year follow-up |
|  |  |  |  | parent_monitor_q3_y | If you are at home when your parents or guardians are not, how often do you know how to get in touch with them? | Youth | 1-year follow-up |
|  |  |  |  | parent_monitor_q4_y | How often do you talk to your mom/dad or guardian about your plans for the coming day, such as your plans about what will happen at school or what you are going to do with friends? | Youth | 1-year follow-up |
|  |  |  |  | parent_monitor_q5_y | In an average week, how many times do you and your parents/guardians, eat dinner together? | Youth | 1-year follow-up |
| ABCD Parent Adult Self Report Scores Aseba (ASR) (abcd_asrs01) | asr_scr_internal_t | Internalizing Problems ASR Syndrome Scale (t-score) | pasr01 | asr_q14_p | I cry a lot | Parent | baseline |
|  | asr_scr_depress_t | Depressive Problems ASR DSM-5-Oriented Scale (t-score) |  | asr_q18_p | I deliberately try to hurt or kill myself | Parent | baseline |
|  |  |  |  | asr_q24_p | I don't eat as well as I should | Parent | baseline |
| ABCD Parent Adult Self Report Scores Aseba (ASR) (abcd_asrs01) | asr_scr_depress_t | Depressive Problems ASR DSM-5-Oriented Scale (t-score) | pasr01 | asr_q35_p | I feel worthless and inferior | Parent | baseline |
|  |  |  |  | asr_q52_p | I feel too guilty | Parent | baseline |
|  |  |  |  | asr_q54_p | I feel tired without good reason | Parent | baseline |
|  |  |  |  | asr_q60_p | There is very little I enjoy | Parent | baseline |
|  |  |  |  | asr_q77_p | I sleep more than most other people during day and/or night | Parent | baseline |
|  |  |  |  | asr_q78_p | I have trouble making decisions | Parent | baseline |
|  |  |  |  | asr_q91_p | I think about killing myself | Parent | baseline |
|  |  |  |  | asr_q96_p | I think about sex too much | Parent | baseline |
|  |  |  |  | asr_q100_p | I have trouble sleeping | Parent | baseline |
|  |  |  |  | asr_q102_p | I don't have much energy | Parent | baseline |
|  |  |  |  | asr_q103_p | I am unhappy, sad, or depressed | Parent | baseline |
|  |  |  |  | asr_q107_p | I feel that I can't succeed | Parent | baseline |
|  | asr_scr_anxdisord_t | Anxiety Problems ASR DSM-5-Oriented Scale (t-score) |  | asr_q22_p | I worry about my future | Parent | baseline |
|  |  |  |  | asr_q29_p | I am afraid of certain animals, situations, or places | Parent | baseline |
|  |  |  |  | asr_q45_p | I am nervous or tense | Parent | baseline |
|  |  |  |  | asr_q50_p | I am too fearful or anxious | Parent | baseline |
|  |  |  |  | asr_q56h_p | Heart pounding or racing | Parent | baseline |
|  |  |  |  | asr_q72_p | I worry about my family | Parent | baseline |
|  |  |  |  | asr_q112_p | I worry a lot | Parent | baseline |
|  | asr_scr_somaticpr_t | Somatic Problems ASR DSM-5-Oriented Scale (t-score) |  | asr_q51_p | I feel dizzy or lightheaded | Parent | baseline |
|  |  |  |  | asr_q56a_p | Aches or pains (not stomach or headaches) | Parent | baseline |
|  |  |  |  | asr_q56b_p | Headaches | Parent | baseline |
|  |  |  |  | asr_q56c_p | Nausea, feel sick | Parent | baseline |
|  |  |  |  | asr_q56d_p | Problems with eyes (not if corrected by glasses) | Parent | baseline |
|  |  |  |  | asr_q56e_p | Rashes or other skin problems | Parent | baseline |
|  |  |  |  | asr_q56f_p | Stomachaches | Parent | baseline |
|  |  |  |  | asr_q56g_p | Vomiting, throwing up | Parent | baseline |
|  |  |  |  | asr_q56i_p | Numbness or tingling in body parts | Parent | baseline |
|  | asr_scr_external_t | Externalizing Problems ASR Syndrome Scale (t-score) |  | asr_q25_p | I don't get along with other people | Parent | baseline |
|  | asr_scr_avoidant_t | Avoidant Personality Problems ASR DSM-5-Oriented Scale (t-score) |  | asr_q42_p | I would rather be alone than with others | Parent | baseline |
|  |  |  |  | asr_q47_p | I lack self-confidence | Parent | baseline |
|  |  |  |  | asr_q67_p | I have trouble making or keeping friends | Parent | baseline |
|  |  |  |  | asr_q71_p | I am self-conscious or easily embarrassed | Parent | baseline |
|  |  |  |  | asr_q75_p | I am too shy or timid | Parent | baseline |
|  |  |  |  | asr_q111_p | I keep from getting involved with others | Parent | baseline |
|  | asr_scr_antisocial_t | Antisocial Personality Problems ASR DSM-5-Oriented Scale (t-score) |  | asr_q03_p | I argue a lot | Parent | baseline |
|  |  |  |  | asr_q05_p | I blame others for my problems | Parent | baseline |
|  |  |  |  | asr_q16_p | I am mean to others | Parent | baseline |
| ABCD Parent Adult Self Report Scores Aseba (ASR) (abcd_asrs01) | asr_scr_antisocial_t | Antisocial Personality Problems ASR DSM-5-Oriented Scale (t-score) | pasr01 | asr_q21_p | I damage or destroy things belonging to others | Parent | baseline |
|  |  |  |  | asr_q23_p | I break rules at work or elsewhere | Parent | baseline |
|  |  |  |  | asr_q26_p | I don't feel guilty after doing something I shouldn't | Parent | baseline |
|  |  |  |  | asr_q28_p | I get along badly with my family | Parent | baseline |
|  |  |  |  | asr_q37_p | I get in many fights | Parent | baseline |
|  |  |  |  | asr_q39_p | I hang around people who get into trouble | Parent | baseline |
|  |  |  |  | asr_q43_p | I lie or cheat | Parent | baseline |
|  |  |  |  | asr_q57_p | I physically attack people | Parent | baseline |
|  |  |  |  | asr_q76_p | My behavior is irresponsible | Parent | baseline |
|  |  |  |  | asr_q82_p | I steal | Parent | baseline |
|  |  |  |  | asr_q92_p | I do things that may cause me trouble with the law | Parent | baseline |
|  |  |  |  | asr_q95_p | I have a hot temper | Parent | baseline |
|  |  |  |  | asr_q97_p | I threaten to hurt people | Parent | baseline |
|  |  |  |  | asr_q101_p | I stay away from my job even when I'm not sick or not on vacation | Parent | baseline |
|  |  |  |  | asr_q114_p | I fail to pay my debts or meet other financial responsibilities | Parent | baseline |
|  |  |  |  | asr_q120_p | I drive too fast | Parent | baseline |
|  |  |  |  | asr_q122_p | I have trouble keeping a job | Parent | baseline |
|  | asr_scr_adhd_t | ADHD Problems ASR DSM-5-Oriented Scale (t-score) |  | asr_q01_p | I am too forgetful | Parent | baseline |
|  | asr_scr_inattention_t | Inattention ASR DSM-5-Oriented Scale (t-score) |  | asr_q08_p | I have trouble concentrating or paying attention for long | Parent | baseline |
|  |  |  |  | asr_q59_p | I fail to finish things I should do | Parent | baseline |
|  |  |  |  | asr_q61_p | My work performance is poor | Parent | baseline |
|  |  |  |  | asr_q108_p | I tend to lose things | Parent | baseline |
|  |  |  |  | asr_q119_p | I am not good at details | Parent | baseline |
|  | asr_scr_hyperactive_t | Hyperactivity-Impulsivity ASR DSM-5-Oriented Scale (t-score) |  | asr_q10_p | I have trouble sitting still | Parent | baseline |
|  |  |  |  | asr_q36_p | I accidentally get hurt a lot, accident-prone | Parent | baseline |
|  |  |  |  | asr_q41_p | I am impulsive or act without thinking | Parent | baseline |
|  |  |  |  | asr_q89_p | I rush into things without considering the risks | Parent | baseline |
|  |  |  |  | asr_q105_p | People think I am disorganized | Parent | baseline |
|  |  |  |  | asr_q115_p | I feel restless or fidgety | Parent | baseline |
|  |  |  |  | asr_q118_p | I am too impatient | Parent | baseline |
|  | asr_scr_totprob_t | Total Problems ASR Syndrome Scale (t-score) |  | asr_q02_p | I make good use of my opportunities | Parent | baseline |
|  |  |  |  | asr_q04_p | I work up to my ability | Parent | baseline |
|  |  |  |  | asr_q06_p | I use drugs (other than alcohol, nicotine) for nonmedical purposes | Parent | baseline |
|  |  |  |  | asr_q07_p | I brag | Parent | baseline |
|  |  |  |  | asr_q09_p | I can't get my mind off certain thoughts | Parent | baseline |
|  |  |  |  | asr_q11_p | I am too dependent on others | Parent | baseline |
|  |  |  |  | asr_q12_p | I feel lonely | Parent | baseline |
| ABCD Parent Adult Self Report Scores Aseba (ASR) (abcd_asrs01) | asr_scr_totprob_t | Total Problems ASR Syndrome Scale (t-score) | pasr01 | asr_q13_p | I feel confused or in a fog | Parent | baseline |
|  |  |  |  | asr_q15_p | I am pretty honest | Parent | baseline |
|  |  |  |  | asr_q17_p | I daydream a lot | Parent | baseline |
|  |  |  |  | asr_q19_p | I try to get a lot of attention | Parent | baseline |
|  |  |  |  | asr_q20_p | I damage or destroy my things | Parent | baseline |
|  |  |  |  | asr_q27_p | I am jealous of others | Parent | baseline |
|  |  |  |  | asr_q30_p | My relations with the opposite sex are poor | Parent | baseline |
|  |  |  |  | asr_q31_p | I am afraid I might think or do something bad | Parent | baseline |
|  |  |  |  | asr_q32_p | I feel that I have to be perfect | Parent | baseline |
|  |  |  |  | asr_q33_p | I feel that no one loves me | Parent | baseline |
|  |  |  |  | asr_q34_p | I feel that others are out to get me | Parent | baseline |
|  |  |  |  | asr_q38_p | My relations with neighbors are poor | Parent | baseline |
|  |  |  |  | asr_q39_p | I hang around people who get into trouble | Parent | baseline |
|  |  |  |  | asr_q40_p | I hear sounds and voices that other people think aren't there | Parent | baseline |
|  |  |  |  | asr_q44_p | I feel overwhelmed by my responsibilities | Parent | baseline |
|  |  |  |  | asr_q46_p | Parts of my body twitch or make nervous movements | Parent | baseline |
|  |  |  |  | asr_q48_p | I am not liked by others | Parent | baseline |
|  |  |  |  | asr_q49_p | I can do certain things better than other people | Parent | baseline |
|  |  |  |  | asr_q53_p | I have trouble planning for the future | Parent | baseline |
|  |  |  |  | asr_q55_p | My moods swing between elation and depression | Parent | baseline |
|  |  |  |  | asr_q58_p | I pick my skin or other parts of my body | Parent | baseline |
|  |  |  |  | asr_q62_p | I am poorly coordinated or clumsy | Parent | baseline |
|  |  |  |  | asr_q63_p | I would rather be with older people than with people of my own age | Parent | baseline |
|  |  |  |  | asr_q64_p | I have trouble setting priorities | Parent | baseline |
|  |  |  |  | asr_q65_p | I refuse to talk | Parent | baseline |
|  |  |  |  | asr_q66_p | I repeat certain acts over and over | Parent | baseline |
|  |  |  |  | asr_q68_p | I scream or yell a lot | Parent | baseline |
|  |  |  |  | asr_q69_p | I am secretive or keep things to myself | Parent | baseline |
|  |  |  |  | asr_q70_p | I see things that other people think aren't there | Parent | baseline |
|  |  |  |  | asr_q73_p | I meet my responsibilities to my family | Parent | baseline |
|  |  |  |  | asr_q74_p | I show off or clown | Parent | baseline |
|  |  |  |  | asr_q79_p | I have a speech problem | Parent | baseline |
|  |  |  |  | asr_q80_p | I stand up for my rights | Parent | baseline |
|  |  |  |  | asr_q81_p | My behavior is very changeable | Parent | baseline |
|  |  |  |  | asr_q83_p | I am easily bored | Parent | baseline |
|  |  |  |  | asr_q84_p | I do things that other people think are strange | Parent | baseline |
|  |  |  |  | asr_q85_p | I have thoughts that other people would think are strange | Parent | baseline |
|  |  |  |  | asr_q86_p | I am stubborn, sullen, or irritable | Parent | baseline |
| ABCD Parent Adult Self Report Scores Aseba (ASR) (abcd_asrs01) | asr_scr_totprob_t | Total Problems ASR Syndrome Scale (t-score) | pasr01 | asr_q87_p | My moods or feeling change suddenly | Parent | baseline |
|  |  |  |  | asr_q88_p | I enjoy being with people | Parent | baseline |
|  |  |  |  | asr_q90_p | I drink too much alcohol or get drunk | Parent | baseline |
|  |  |  |  | asr_q93_p | I talk too much | Parent | baseline |
|  |  |  |  | asr_q94_p | I tease others a lot | Parent | baseline |
|  |  |  |  | asr_q98_p | I like to help others | Parent | baseline |
|  |  |  |  | asr_q99_p | I dislike staying in one place for very long | Parent | baseline |
|  |  |  |  | asr_q104_p | I am louder than others | Parent | baseline |
|  |  |  |  | asr_q106_p | I try to be fair to others | Parent | baseline |
|  |  |  |  | asr_q109_p | I like to try new things | Parent | baseline |
|  |  |  |  | asr_q110_p | I wish I were of the opposite sex | Parent | baseline |
|  |  |  |  | asr_q113_p | I worry about my relations with the opposite sex | Parent | baseline |
|  |  |  |  | asr_q116_p | I get upset too easily | Parent | baseline |
|  |  |  |  | asr_q117_p | I have trouble managing my money or credit card | Parent | baseline |
|  |  |  |  | asr_q121_p | I tend to be late for appointments | Parent | baseline |
|  |  |  |  | asr_q123_p | I am a happy person | Parent | baseline |
|  |  |  |  | asr_q124_p | In the past 6 months, about how many times per day did you use tobacco (including smokeless tobacco)? | Parent | baseline |
|  |  |  |  | asr_q125_p | In the past 6 months, on how many days were you drunk? | Parent | baseline |
|  |  |  |  | asr_q126_p | In the past 6 months, on how many days did you use drugs for nonmedical purposes (including marijuana, cocaine, and other drugs, except alcohol and nicotine)? | Parent | baseline |
| Sum Scores Traumatic Brain Injury (abcd_tbi01) | tbi_ss_nmrpi | Number of periods with multiple or repeated injuries: tbi_7a + tbi_7g + tbi_8g | abcd_otbi01 | tbi_7a | Did your child experience a period of time in his/her life when he/she experienced multiple, repeated impacts to the head? (e.g., abused, contact sports) | Parent | baseline |
|  |  |  |  | tbi_7g | Was there another instance where your child experienced a period of time in his/her life when he/she experienced multiple, repeated impacts to the head? | Parent | baseline |
|  |  |  |  | tbi_8g | Was there another instance where your child experienced a period of time in his/her life when he/she experienced multiple, repeated impacts to the head? | Parent | baseline |
|  | tbi_ss_worst_overall | Worst Injury Overall (Improbable TBI; Possible mild TBI; Mild TBI; Moderate TBI; Severe TBI) |  | tbi_1 | Has your child ever been hospitalized or treated in an emergency room following an injury to his/her head or neck? | Parent | baseline |
|  |  |  |  | tbi_1b | Was he/she knocked out or did he/she lose consciousness (LOC)? If yes, how long? | Parent | baseline |
|  |  |  |  | tbi_1c | Was he/she dazed or did he/she have a gap in his/her memory from the injury? | Parent | baseline |
|  |  |  |  | tbi_2 | Has your child ever injured his/her head or neck in a car accident or from crashing some other moving vehicle like a bicycle, motorcyle, or ATV? | Parent | baseline |
|  |  |  |  | tbi_2b | Was he/she knocked out or did he/she lose consciousness (LOC)? If yes, how long? | Parent | baseline |
| Sum Scores Traumatic Brain Injury (abcd_tbi01) | tbi_ss_worst_overall | Worst Injury Overall (Improbable TBI; Possible mild TBI; Mild TBI; Moderate TBI; Severe TBI) | abcd_otbi01 | tbi_2c | Was he/she dazed or did he/she have a gap in his/her memory from the injury? | Parent | baseline |
|  |  |  |  | tbi_3 | Has you child ever injured his/her head or neck in a fall or from being hit by something? (For example, falling from a bike or horse, rollerblading, falling on ice, being hit by a rock) Has your child ever injured his/her head or neck playing sports or on the playground? | Parent | baseline |
|  |  |  |  | tbi_3b | Was he/she knocked out or did he/she lose consciousness (LOC)? If yes, how long? | Parent | baseline |
|  |  |  |  | tbi_3c | Was he/she dazed or did he/she have a gap in his/her memory from the injury? | Parent | baseline |
|  |  |  |  | tbi_4 | Has your child ever injured his/her head or neck in a fight, from being hit by something, or from being shaken violently? Has your child ever been shot in the head? | Parent | baseline |
|  |  |  |  | tbi_4b | Was he/she knocked out or did he/she lose consciousness (LOC)? If yes, how long? | Parent | baseline |
|  |  |  |  | tbi_4c | Was he/she dazed or did he/she have a gap in his/her memory from the injury? | Parent | baseline |
|  |  |  |  | tbi_5 | Has your child ever been nearby when an explosion or blast has occurred? | Parent | baseline |
|  |  |  |  | tbi_5b | Was he/she knocked out or did he/she lose consciousness (LOC)? If yes, how long? | Parent | baseline |
|  |  |  |  | tbi_5c | Was he/she dazed or did he/she have a gap in his/her memory from the injury? | Parent | baseline |
|  |  |  |  | tbi_6o | Do you want to report any more injuries with LOC? | Parent | baseline |
|  |  |  |  | tbi_6p | If yes, how many more? | Parent | baseline |
|  |  |  |  | tbi_6q | How many minutes was the longest knock out? | Parent | baseline |
|  |  |  |  | tbi_6r | How many were more than or equal to 30 min? | Parent | baseline |
|  |  |  |  | tbi_7a | Did your child experience a period of time in his/her life when he/she experienced multiple, repeated impacts to the head? (e.g., abused, contact sports) | Parent | baseline |
|  |  |  |  | tbi_7c1 | Was he/she knocked out or did he/she lose consciousness (LOC)? If yes, how long? | Parent | baseline |
|  |  |  |  | tbl_7c2 | Was he/she dazed or did he/she have a gap in his/her memory from the injury? | Parent | baseline |
|  |  |  |  | tbi_7e | At what age did these effects begin? | Parent | baseline |
|  |  |  |  | tbi_7f | At what age did these effects end? | Parent | baseline |
|  |  |  |  | tbi_7g | Was there another instance where your child experienced a period of time in his/her life when he/she experienced multiple, repeated impacts to the head? | Parent | baseline |
|  |  |  |  | tbi_7i | What was the typical effect of the injury? | Parent | baseline |
|  |  |  |  | tbi_7k | At what age did these effects begin? | Parent | baseline |
|  |  |  |  | tbi_7l | At what age did these effects end? | Parent | baseline |
| Sum Scores Traumatic Brain Injury (abcd_tbi01) | tbi_ss_worst_overall | Worst Injury Overall (Improbable TBI; Possible mild TBI; Mild TBI; Moderate TBI; Severe TBI) | abcd_otbi01 | tbi_8g | Was there another instance where your child experienced a period of time in his/her life when he/she experienced multiple, repeated impacts to the head? | Parent | baseline |
|  |  |  |  | tbi_8i | What was the typical effect of the injury? | Parent | baseline |
|  |  |  |  | tbi_8k | At what age did these effects begin? | Parent | baseline |
|  |  |  |  | tbi_8l | At what age did these effects end? | Parent | baseline |
| ABCD Parent Community Risk and Protective Factors (CRPF) (abcd_crpf01) | su_risk_p_1 | If your child wanted to get some beer, wine, or hard liquor (for example vodka, whiskey, or gin), how easy would it be for her/him to get some? |  |  |  | Parent | 1-year follow-up |
|  | su_risk_p_2 | If your child wanted to get some cigarettes, how easy would it be for her/him to get some? |  |  |  | Parent | 1-year follow-up |
|  | su_risk_p_3 | If your child wanted to get some e-cigarettes, vape pens, or e-hookah, how easy would it be for her/him to get some? |  |  |  | Parent | 1-year follow-up |
|  | su_risk_p_4 | If your child wanted to get some marijuana, how easy would it be for her/him to get some? |  |  |  | Parent | 1-year follow-up |
|  | su_risk_p_5 | If your child wanted to get a drug like cocaine, LSD, or amphetamines, how easy would it be for her/him to get some? |  |  |  | Parent | 1-year follow-up |
|  | su_risk_p_6 | Is "medical marijuana" (marijuana prescribed by a doctor) legal in your state? |  |  |  | Parent | 1-year follow-up |
| ABCD Youth Neighborhood Safety/Crime Survey Modified from PhenX (NSC) (abcd_nsc01) | neighborhood_crime_y | "My neighborhood is safe from crime" |  |  |  | Youth | 1-year follow-up |
| ABCD Youth Screen Time Survey (STQ) (abcd_stq01) | screen1_wkdy_y | On a typical weekday, how many hours do you watch TV shows or movies? |  |  |  | Youth | 1-year follow-up |
|  | screen2_wkdy_y | On a typical weekday, how many hours do you watch videos (such as YouTube)? |  |  |  | Youth | 1-year follow-up |
|  | screen3_wkdy_y | On a typical weekday, how many hours do you play video games on a computer, console, phone or other device (Xbox, Play Station, iPad)? |  |  |  | Youth | 1-year follow-up |
|  | screen4_wkdy_y | On a typical weekday, how many hours do you text on a cell phone, tablet, or computer (e.g. GChat, Whatsapp, etc.)? |  |  |  | Youth | 1-year follow-up |
|  | screen5_wkdy_y | On a typical weekday, how many hours do you visit social networking sites like Facebook, Twitter, Instagram, etc.? |  |  |  | Youth | 1-year follow-up |
|  | screen_wkdy_y | On a typical weekday, how many hours do you video chat (Skype, Facetime, etc.)? |  |  |  | Youth | 1-year follow-up |
|  | screen7_wknd_y | On a typical weekend day, how many hours do you watch TV shows or movies? |  |  |  | Youth | 1-year follow-up |
|  | screen8_wknd_y | On a typical weekend day, how many hours do you watch videos (such as YouTube)? |  |  |  | Youth | 1-year follow-up |
|  | screen9_wknd_y | On a typical weekend day, how many hours do you play video games on a computer, console, phone or other device (Xbox, Play Station, iPad)? |  |  |  | Youth | 1-year follow-up |
| ABCD Youth Screen Time Survey (STQ) (abcd_stq01) | screen10_wknd_y | On a typical weekend day, how many hours do you text on a cell phone, tablet, or computer (GChat, Whatsapp, etc.)? |  |  |  | Youth | 1-year follow-up |
|  | screen11_wknd_y | On a typical weekend day, how many hours do you visit social networking sites like Facebook, Twitter, Instagram, etc.? |  |  |  | Youth | 1-year follow-up |
|  | screen12_wknd_y | On a typical weekend day, how many hours do you video chat (Skype, Facetime, etc.)? |  |  |  | Youth | 1-year follow-up |
|  | screen13_y | How often do you play mature-rated video games (e.g., Call of Duty, Grand Theft Auto, Assassin's Creed, etc.)? |  |  |  | Youth | 1-year follow-up |
|  | screen14_y | How often do you watch R-rated movies? |  |  |  | Youth | 1-year follow-up |
| ABCD Parental Monitoring Survey (pmq01) | parent_monitor_q1_y | How often do your parents/guardians know where you are? |  |  |  | Youth | 1-year follow-up |
|  | parent_monitor_q2_y | How often do your parents know who you are with when you are not at school and away from home? |  |  |  | Youth | 1-year follow-up |
|  | parent_monitor_q3_y | If you are at home when your parents or guardians are not, how often do you know how to get in touch with them? |  |  |  | Youth | 1-year follow-up |
|  | parent_monitor_q4_y | How often do you talk to your mom/dad or guardian about your plans for the coming day, such as your plans about what will happen at school or what you are going to do with friends? |  |  |  | Youth | 1-year follow-up |
|  | parent_monitor_q5_y | In an average week, how many times do you and your parents/guardians, eat dinner together? |  |  |  | Youth | 1-year follow-up |
| ABCD Parental Rules on Substance Use (prq01) | parent_rules_q1 | What are the family rules about drinking alcohol for your son/daughter? |  |  |  | Parent | 1-year follow-up |
|  | parent_rules_q1a | Are these the same rules for all family members? |  |  |  | Parent | 1-year follow-up |
|  | parent_rules_q2 | Do you have penalties for violating family rules about drinking? |  |  |  | Parent | 1-year follow-up |
|  | parent_rules_q4 | What are the family rules about smoking cigarettes for your son/daughter? |  |  |  | Parent | 1-year follow-up |
|  | parent_rules_q5 | Are these the same rules for all family members? |  |  |  | Parent | 1-year follow-up |
|  | parent_rules_q6 | Do you enforce penalties for violating family rules about smoking? |  |  |  | Parent | 1-year follow-up |
|  | parent_rules_q7 | What are the family rules about using marijuana for your son/daughter? |  |  |  | Parent | 1-year follow-up |
| ABCD Youth Discrimination Measure (abcd_ydmes01) | dim_yesno_q1 | In the past 12 months, have you felt discriminated against because of your race, ethnicity, or color? |  |  |  | Youth | 1-year follow-up |
|  | dim_yesno_q2 | In the past 12 months, have you felt discriminated against because you are (or your family is) from another country? |  |  |  | Youth | 1-year follow-up |
|  | dim_yesno_q3 | In the past 12 months, have you felt discriminated against because someone thought you were gay, lesbian, or bisexual? |  |  |  | Youth | 1-year follow-up |
|  | dim_yesno_q4 | In the past 12 months, have you felt discriminated against because of your weight? |  |  |  | Youth | 1-year follow-up |
|  | dim_matrix_q1 | How often do teachers treat you unfairly or negatively because of your ethnic background? |  |  |  | Youth | 1-year follow-up |
| ABCD Youth Discrimination Measure (abcd_ydmes01) | dim_matrix_q2 | How often do other adults outside school treat you unfairly or negatively because of your ethnic background? |  |  |  | Youth | 1-year follow-up |
|  | dim_matrix_q3 | How often do other students treat you unfairly or negatively because of your ethnic background? |  |  |  | Youth | 1-year follow-up |
|  | dim_matrix_q4 | "I feel that others behave in an unfair or negative way toward my ethnic group" |  |  |  | Youth | 1-year follow-up |
|  | dim_matrix_q5 | "I feel that I am not wanted in American society" |  |  |  | Youth | 1-year follow-up |
|  | dim_matrix_q6 | "I don't feel accepted by other Americans" |  |  |  | Youth | 1-year follow-up |
|  | dim_matrix_q7 | "I feel that other Americans have something against me" |  |  |  | Youth | 1-year follow-up |
| ABCD School Risk and Protective Factors Survey (srpf01) | school_2_y | "In my school, students have lots of chances to help decide things like class activities and rules" |  |  |  | Youth | 1-year follow-up |
|  | school_3_y | "I get along with my teachers" |  |  |  | Youth | 1-year follow-up |
|  | school_4_y | "My teacher(s) notices when I am doing a good job and lets me know about it" |  |  |  | Youth | 1-year follow-up |
|  | school_5_y | "There are lots of chances for students in my school to get involved in sports, clubs, or other school activities outside of class" |  |  |  | Youth | 1-year follow-up |
|  | school_6_y | "I feel safe at my school" |  |  |  | Youth | 1-year follow-up |
|  | school_7_y | "The school lets my parents know when I have done something well" |  |  |  | Youth | 1-year follow-up |
|  | school_8_y | "I like school because I do well in class" |  |  |  | Youth | 1-year follow-up |
|  | school_9_y | "I feel I'm just as smart as other kids my age" |  |  |  | Youth | 1-year follow-up |
|  | school_10_y | "There are lots of chances to be part of class discussions or activities" |  |  |  | Youth | 1-year follow-up |
|  | school_12_y | "In general, I like school a lot" |  |  |  | Youth | 1-year follow-up |
|  | school_15_y | "Usually, school bores me" |  |  |  | Youth | 1-year follow-up |
|  | school_17_y | "Getting good grades is not so important to me" |  |  |  | Youth | 1-year follow-up |
| ABCD Parent Acculturation Survey Modified from PhenX (ACC) (pacc01) | accult_q1_p | How well do you speak English? |  |  |  | Parent | 1-year follow-up |
|  | accult_q2_p | Besides English, do you speak or understand another language or dialect? |  |  |  | Parent | 1-year follow-up |
| ABCD Youth Acculturation Survey Modified from PhenX (ACC) (yacc01) | accult_q1_y | How well do you speak English? |  |  |  | Youth | 1-year follow-up |
|  | accult_q2_y | Besides English, do you speak or understand another language or dialect? |  |  |  | Youth | 1-year follow-up |
| ABCD Parent Family History Summary Scores (abcd_fhxssp01) | famhx_ss_fath_prob_alc_p | Father alcohol problem | fhxp102 | famhx_4_p | Has ANY blood relative of your child ever had any problems due to alcohol, such as: Marital separation or divorce; Laid off or fired from work; Arrests or DUIs; Alcohol harmed their health; In an alcohol treatment program; Suspended or expelled from school 2 or more times; Isolated self from family, caused arguments or were drunk a lot. | Parent | baseline |
|  |  |  |  | famhx4a_p___1 | Biological father (Marital) | Parent | baseline |
|  |  |  |  | famhx4a_p___2 | Biological father (Work) | Parent | baseline |
|  |  |  |  | famhx4a_p___3 | Biological father (Arrests/DUI) | Parent | baseline |
|  |  |  |  | famhx4a_p___7 | Biological father (Health) | Parent | baseline |
|  |  |  |  | famhx4a_p___4 | Biological father (Alcohol treatment program) | Parent | baseline |
|  |  |  |  | famhx4a_p___5 | Biological father (School) | Parent | baseline |
|  |  |  |  | famhx4a_p___6 | Biological father (Isolated self, arguments, drunk a lot) | Parent | baseline |
|  | famhx_ss_patgf_prob_alc_p | Paternal grandfather alcohol problem |  | famhx4b_p___1 | Paternal grandfather (Marital) | Parent | baseline |
|  |  |  |  | famhx4b_p___2 | Paternal grandfather (Work) | Parent | baseline |
|  |  |  |  | famhx4b_p___3 | Paternal grandfather (Arrests/DUI) | Parent | baseline |
|  |  |  |  | famhx4b_p___7 | Paternal grandfather (Health) | Parent | baseline |
|  |  |  |  | famhx4b_p___4 | Paternal grandfather (Alcohol treatment program) | Parent | baseline |
|  |  |  |  | famhx4b_p___5 | Paternal grandfather (School) | Parent | baseline |
|  |  |  |  | famhx4b_p___6 | Paternal grandfather (Isolated self, arguments, drunk a lot) | Parent | baseline |
|  | famhx_ss_patgm_prob_alc_p | Paternal grandmother alcohol problem |  | famhx_4c_p___1 | Paternal grandmother (Marital) | Parent | baseline |
|  |  |  |  | famhx_4c_p___2 | Paternal grandmother (Work) | Parent | baseline |
|  |  |  |  | famhx_4c_p___3 | Paternal grandmother (Arrests/DUI) | Parent | baseline |
|  |  |  |  | famhx_4c_p___7 | Paternal grandmother (Health) | Parent | baseline |
|  |  |  |  | famhx_4c_p___4 | Paternal grandmother (Alcohol treatment program) | Parent | baseline |
|  |  |  |  | famhx_4c_p___5 | Paternal grandmother (School) | Parent | baseline |
|  |  |  |  | famhx_4c_p___6 | Paternal grandmother (Isolated self, arguments, drunk a lot) | Parent | baseline |
|  | famhx_ss_moth_prob_alc_p | Mother alcohol problem |  | famhx_4d_p___1 | Biological mother (Marital) | Parent | baseline |
|  |  |  |  | famhx_4d_p___2 | Biological mother (Work) | Parent | baseline |
|  |  |  |  | famhx_4d_p___3 | Biological mother (Arrests/DUI) | Parent | baseline |
|  |  |  |  | famhx_4d_p___7 | Biological mother (Health) | Parent | baseline |
|  |  |  |  | famhx_4d_p___4 | Biological mother (Alcohol treatment program) | Parent | baseline |
|  |  |  |  | famhx_4d_p___5 | Biological mother (School) | Parent | baseline |
|  |  |  |  | famhx_4d_p___6 | Biological mother (Isolated self, arguments, drunk a lot) | Parent | baseline |
|  | famhx_ss_matgf_prob_alc_p | Maternal grandfather alcohol problem |  | famhx_4e_p___1 | Maternal grandfather (Marital) | Parent | baseline |
|  |  |  |  | famhx_4e_p___2 | Maternal grandfather (Work) | Parent | baseline |
|  |  |  |  | famhx_4e_p___3 | Maternal grandfather (Arrests/DUI) | Parent | baseline |
|  |  |  |  | famhx_4e_p___7 | Maternal grandfather (Health) | Parent | baseline |
|  |  |  |  | famhx_4e_p___4 | Maternal grandfather (Alcohol treatment program) | Parent | baseline |
| ABCD Parent Family History Summary Scores (abcd_fhxssp01) | famhx_ss_matgf_prob_alc_p | Maternal grandfather alcohol problem | fhxp102 | famhx_4e_p___5 | Maternal grandfather (School) | Parent | baseline |
|  |  |  |  | famhx_4e_p___6 | Maternal grandfather (Isolated self, arguments, drunk a lot) | Parent | baseline |
|  | famhx_ss_matgm_prob_alc_p | Maternal grandmother alcohol problem |  | famhx_4f_p___1 | Maternal grandmother (Marital) | Parent | baseline |
|  |  |  |  | famhx_4f_p___2 | Maternal grandmother (Work) | Parent | baseline |
|  |  |  |  | famhx_4f_p___3 | Maternal grandmother (Arrests/DUI) | Parent | baseline |
|  |  |  |  | famhx_4f_p___7 | Maternal grandmother (Health) | Parent | baseline |
|  |  |  |  | famhx_4f_p___4 | Maternal grandmother (Alcohol treatment program) | Parent | baseline |
|  |  |  |  | famhx_4f_p___5 | Maternal grandmother (School) | Parent | baseline |
|  |  |  |  | famhx_4f_p___6 | Maternal grandmother (Isolated self, arguments, drunk a lot) | Parent | baseline |
|  | famhx_ss_fath_prob_dg_p | Father drug use problem |  | fam_history_5_yes_no | Has ANY blood relative of your child ever had any problems due to drugs, such as: Marital separation or divorce; Laid off or fired from work; Arrests or DUIs; Drugs harmed their health; In a drug treatment program; Suspended or expelled from school 2 or more times; Isolated self from family, caused arguments or were high a lot. | Parent | baseline |
|  |  |  |  | fam_history_q5a_drugs___1 | Biological father (Marital) | Parent | baseline |
|  |  |  |  | fam_history_q5a_drugs___2 | Biological father (Work) | Parent | baseline |
|  |  |  |  | fam_history_q5a_drugs___3 | Biological father (Arrests/DUI) | Parent | baseline |
|  |  |  |  | fam_history_q5a_drugs___7 | Biological father (Health) | Parent | baseline |
|  |  |  |  | fam_history_q5a_drugs___4 | Biological father (Drug treatment program) | Parent | baseline |
|  |  |  |  | fam_history_q5a_drugs___5 | Biological father (School) | Parent | baseline |
|  |  |  |  | fam_history_q5a_drugs___6 | Biological father (Isolated self, arguments, high a lot) | Parent | baseline |
|  | famhx_ss_patgf_prob_dg_p | Paternal grandfather drug use problem |  | fam_history_q5b_drugs___1 | Paternal grandfather (Marital) | Parent | baseline |
|  |  |  |  | fam_history_q5b_drugs___2 | Paternal grandfather (Work) | Parent | baseline |
|  |  |  |  | fam_history_q5b_drugs___3 | Paternal grandfather (Arrests/DUI) | Parent | baseline |
|  |  |  |  | fam_history_q5b_drugs___7 | Paternal grandfather (Health) | Parent | baseline |
|  |  |  |  | fam_history_q5b_drugs___4 | Paternal grandfather (Drug treatment program) | Parent | baseline |
|  |  |  |  | fam_history_q5b_drugs___5 | Paternal grandfather (School) | Parent | baseline |
|  |  |  |  | fam_history_q5b_drugs___6 | Paternal grandfather (Isolated self, arguments, high a lot) | Parent | baseline |
|  | famhx_ss_patgm_prob_dg_p | Paternal grandmother drug use problem |  | fam_history_q5c_drugs___1 | Paternal grandmother (Marital) | Parent | baseline |
|  |  |  |  | fam_history_q5c_drugs___2 | Paternal grandmother (Work) | Parent | baseline |
|  |  |  |  | fam_history_q5c_drugs___3 | Paternal grandmother (Arrests/DUI) | Parent | baseline |
|  |  |  |  | fam_history_q5c_drugs___7 | Paternal grandmother (Health) | Parent | baseline |
|  |  |  |  | fam_history_q5c_drugs___4 | Paternal grandmother (Drug treatment program) | Parent | baseline |
|  |  |  |  | fam_history_q5c_drugs___5 | Paternal grandmother (School) | Parent | baseline |
|  |  |  |  | fam_history_q5c_drugs___6 | Paternal grandmother (Isolated self, arguments, high a lot) | Parent | baseline |
| ABCD Parent Family History Summary Scores (abcd_fhxssp01) | famhx_ss_moth_prob_dg_p | Mother drug use problem | fhxp102 | fam_history_q5d_drugs___1 | Biological mother (Marital) | Parent | baseline |
|  |  |  |  | fam_history_q5d_drugs___2 | Biological mother (Work) | Parent | baseline |
|  |  |  |  | fam_history_q5d_drugs___3 | Biological mother (Arrests/DUI) | Parent | baseline |
|  |  |  |  | fam_history_q5d_drugs___7 | Biological mother (Health) | Parent | baseline |
|  |  |  |  | fam_history_q5d_drugs___4 | Biological mother (Drug treatment program) | Parent | baseline |
|  |  |  |  | fam_history_q5d_drugs___5 | Biological mother (School) | Parent | baseline |
|  |  |  |  | fam_history_q5d_drugs___6 | Biological mother (Isolated self, arguments, high a lot) | Parent | baseline |
|  | famhx_ss_matgf_prob_dg_p | Maternal grandfather drug use problem |  | fam_history_q5e_drugs___1 | Maternal grandfather (Marital) | Parent | baseline |
|  |  |  |  | fam_history_q5e_drugs___2 | Maternal grandfather (Work) | Parent | baseline |
|  |  |  |  | fam_history_q5e_drugs___3 | Maternal grandfather (Arrests/DUI) | Parent | baseline |
|  |  |  |  | fam_history_q5e_drugs___7 | Maternal grandfather (Health) | Parent | baseline |
|  |  |  |  | fam_history_q5e_drugs___4 | Maternal grandfather (Drug treatment program) | Parent | baseline |
|  |  |  |  | fam_history_q5e_drugs___5 | Maternal grandfather (School) | Parent | baseline |
|  |  |  |  | fam_history_q5e_drugs___6 | Maternal grandfather (Isolated self, arguments, high a lot) | Parent | baseline |
|  | famhx_ss_matgm_prob_dg_p | Maternal grandmother drug use problem |  | fam_history_q5f_drugs___1 | Maternal grandmother (Marital) | Parent | baseline |
|  |  |  |  | fam_history_q5f_drugs___2 | Maternal grandmother (Work) | Parent | baseline |
|  |  |  |  | fam_history_q5f_drugs___3 | Maternal grandmother (Arrests/DUI) | Parent | baseline |
|  |  |  |  | fam_history_q5f_drugs___7 | Maternal grandmother (Health) | Parent | baseline |
|  |  |  |  | fam_history_q5f_drugs___4 | Maternal grandmother (Drug treatment program) | Parent | baseline |
|  |  |  |  | fam_history_q5f_drugs___5 | Maternal grandmother (School) | Parent | baseline |
|  |  |  |  | fam_history_q5f_drugs___6 | Maternal grandmother (Isolated self, arguments, high a lot) | Parent | baseline |
|  | famhx_ss_fath_prob_dprs_p | Father depression problem |  | fam_history_6_yes_no | Has ANY blood relative of your child ever suffered from depression, that is, have they felt so low for a period of at least two weeks that they hardly ate or slept or couldn't work or do whatever they usually do? | Parent | baseline |
|  |  |  |  | fam_history_q6a_depression | Biological father | Parent | baseline |
|  | famhx_ss_patgf_prob_dprs_p | Paternal grandfather depression problem |  | fam_history_q6b_depression | Paternal grandfather | Parent | baseline |
|  | famhx_ss_patgm_prob_dprs_p | Paternal grandmother depression problem |  | fam_history_q6c_depression | Paternal grandmother | Parent | baseline |
|  | famhx_ss_moth_prob_dprs_p | Mother depression problem |  | fam_history_q6d_depression | Biological mother | Parent | baseline |
|  | famhx_ss_matgf_prob_dprs_p | Maternal grandfather depression problem |  | fam_history_q6e_depression | Maternal grandfather | Parent | baseline |
|  | famhx_ss_matgm_prob_dprs_p | Maternal grandmother depression problem |  | fam_history_q6f_depression | Maternal grandmother | Parent | baseline |
| ABCD Parent Family History Summary Scores (abcd_fhxssp01) | famhx_ss_fath_prob_ma_p | Father mania problem | fhxp201 | fam_history_7_yes_no | Has ANY blood relative of your child ever had a period of time when others were concerned because they suddenly became more active day and night and seemed not to need any sleep and talked much more than usual for them? | Parent | baseline |
|  |  |  |  | fam_history_q7a_mania | Biological father | Parent | baseline |
|  | famhx_ss_patgf_prob_ma_p | Paternal grandfather mania problem |  | fam_history_q7b_mania | Paternal grandfather | Parent | baseline |
|  | famhx_ss_patgm_prob_ma_p | Paternal grandmother mania problem |  | fam_history_q7c_mania | Paternal grandmother | Parent | baseline |
|  | famhx_ss_moth_prob_ma_p | Mother mania problem |  | fam_history_q7d_mania | Biological mother | Parent | baseline |
|  | famhx_ss_matgf_prob_ma_p | Maternal grandfather mania problem |  | fam_history_q7e_mania | Maternal grandfather | Parent | baseline |
|  | famhx_ss_matgm_prob_ma_p | Maternal grandmother mania problem |  | fam_history_q7f_mania | Maternal grandmother | Parent | baseline |
|  | famhx_ss_fath_prob_vs_p | Father visions of others spying/plotting problem |  | fam_history_8_yes_no | Has ANY blood relative of your child ever had a period lasting six months when they saw visions or heard voices or thought people were spying on them or plotting against them? | Parent | baseline |
|  |  |  |  | fam_history_q8a_visions | Biological father | Parent | baseline |
|  | famhx_ss_patgf_prob_vs_p | Paternal grandfather visions of others spying/plotting problem |  | fam_history_q8b_visions | Paternal grandfather | Parent | baseline |
|  | famhx_ss_patgm_prob_vs_p | Paternal grandmother visions of others spying/plotting problem |  | fam_history_q8c_visions | Paternal grandmother | Parent | baseline |
|  | famhx_ss_moth_prob_vs_p | Mother visions of others spying/plotting problem |  | fam_history_q8d_visions | Biological mother | Parent | baseline |
|  | famhx_ss_matgf_prob_vs_p | Maternal grandfather visions of others spying/plotting problem |  | fam_history_q8e_visions | Maternal grandfather | Parent | baseline |
|  | famhx_ss_matgm_prob_vs_p | Maternal grandmother visions of others spying/plotting problem |  | fam_history_q8f_visions | Maternal grandmother | Parent | baseline |
|  | famhx_ss_fath_prob_trb_p | Father trouble holds job/fights/police problem |  | fam_history_9_yes_no | Has ANY blood relative of your child been the kind of person who never holds a job for long, or gets into fights, or gets into trouble with the police from time to time, or had any trouble with the law as a child or an adult? | Parent | baseline |
|  |  |  |  | fam_history_q9a_trouble | Biological father | Parent | baseline |
|  | famhx_ss_patgf_prob_trb_p | Paternal grandfather trouble holds job/fights/police problem |  | fam_history_q9b_trouble | Paternal grandfather | Parent | baseline |
|  | famhx_ss_patgm_prob_trb_p | Paternal grandmother trouble holds job/fights/police problem |  | fam_history_q9c_trouble | Paternal grandmother | Parent | baseline |
|  | famhx_ss_moth_prob_trb_p | Mother trouble holds job/fights/police problem |  | fam_history_q9d_trouble | Biological mother | Parent | baseline |
|  | famhx_ss_matgf_prob_trb_p | Maternal grandfather trouble holds job/fights/police problem |  | fam_history_q9e_trouble | Maternal grandfather | Parent | baseline |
|  | famhx_ss_matgm_prob_trb_p | Maternal grandmother trouble holds job/fights/police problem |  | fam_history_q9f_trouble | Maternal grandmother | Parent | baseline |
|  | famhx_ss_fath_prob_nrv_p | Father nerves/nervous breakdown problem |  | fam_history_10_yes_no | Has ANY blood relative of your child ever had any other problems with their nerves, or had a nervous breakdown? | Parent | baseline |
|  |  |  |  | fam_history_q10a_nerves | Biological father | Parent | baseline |
|  | famhx_ss_patgf_prob_nrv_p | Paternal grandfather nerves/nervous breakdown problem |  | fam_history_q10b_nerves | Paternal grandfather | Parent | baseline |
|  | famhx_ss_patgm_prob_nrv_p | Paternal grandmother nerves/nervous breakdown problem |  | fam_history_q10c_nerves | Paternal grandmother | Parent | baseline |
|  | famhx_ss_moth_prob_nrv_p | Mother nerves/nervous breakdown problem |  | fam_history_q10d_nerves | Biological mother | Parent | baseline |
|  | famhx_ss_matgf_prob_nrv_p | Maternal grandfather nerves/nervous breakdown problem |  | fam_history_q10e_nerves | Maternal grandfather | Parent | baseline |
|  | famhx_ss_matgm_prob_nrv_p | Maternal grandmother nerves/nervous breakdown problem |  | fam_history_q10f_nerves | Maternal grandmother | Parent | baseline |
|  | famhx_ss_fath_prob_prf_p | Father been to a doctor or counselor due to emotional/mental problem |  | fam_history_11_yes_no | Has ANY blood relative of your child ever been to a doctor or a counselor about any emotional or mental problems, or problems with alcohol or drugs? | Parent | baseline |
| ABCD Parent Family History Summary Scores (abcd_fhxssp01) | famhx_ss_fath_prob_prf_p | Father been to a doctor or counselor due to emotional/mental problem | fhxp201 | fam_history_q11a_professional | Biological father | Parent | baseline |
|  | famhx_ss_patgf_prob_prf_p | Paternal grandfather been to a doctor or counselor due to emotional/mental problem |  | fam_history_q11b_professional | Paternal grandfather | Parent | baseline |
|  | famhx_ss_patgm_prob_prf_p | Paternal grandmother been to a doctor or counselor due to emotional/mental problem |  | fam_history_q11c_professional | Paternal grandmother | Parent | baseline |
|  | famhx_ss_moth_prob_prf_p | Mother been to a doctor or counselor due to emotional/mental problem |  | fam_history_q11d_professional | Biological mother | Parent | baseline |
|  | famhx_ss_matgf_prob_prf_p | Maternal grandfather been to a doctor or counselor due to emotional/mental problem |  | fam_history_q11e_professional | Maternal grandfather | Parent | baseline |
|  | famhx_ss_matgm_prob_prf_p | Maternal grandmother been to a doctor or counselor due to emotional/mental problem |  | fam_history_q11f_professional | Maternal grandmother | Parent | baseline |
|  | famhx_ss_fath_prob_hspd_p | Father hospitalized due to emotional/mental problem |  | fam_history_12_yes_no | Has ANY blood relative of your child ever been hospitalized because of emotional or mental problems, or drug or alcohol problems? | Parent | baseline |
|  |  |  |  | fam_history_q12a_hospitalized | Biological father | Parent | baseline |
|  | famhx_ss_patgf_prob_hspd_p | Paternal grandfather hospitalized due to emotional/mental problem |  | fam_history_q12b_hospitalized | Paternal grandfather | Parent | baseline |
|  | famhx_ss_patgm_prob_hspd_p | Paternal grandmother hospitalized due to emotional/mental problem |  | fam_history_q12c_hospitalized | Paternal grandmother | Parent | baseline |
|  | famhx_ss_moth_prob_hspd_p | Mother hospitalized due to emotional/mental problem |  | fam_history_q12d_hospitalized | Biological mother | Parent | baseline |
|  | famhx_ss_matgf_prob_hspd_p | Maternal grandfather hospitalized due to emotional/mental problem |  | fam_history_q12e_hospitalized | Maternal grandfather | Parent | baseline |
|  | famhx_ss_matgm_prob_hspd_p | Maternal grandmother hospitalized due to emotional/mental problem |  | fam_history_q12f_hospitalized | Maternal grandmother | Parent | baseline |
|  | famhx_ss_fath_prob_scd_p | Father attempted or committed suicide |  | fam_history_13_yes_no | Has ANY blood relative of your child ever attempted or committed suicide? | Parent | baseline |
|  |  |  |  | fam_history_q13a_suicide | Biological father | Parent | baseline |
|  | famhx_ss_patgf_prob_scd_p | Paternal grandfather attempted or committed suicide |  | fam_history_q13b_suicide | Paternal grandfather | Parent | baseline |
|  | famhx_ss_patgm_prob_scd_p | Paternal grandmother attempted or committed suicide |  | fam_history_q13c_suicide | Paternal grandmother | Parent | baseline |
|  | famhx_ss_moth_prob_scd_p | Mother attempted or committed suicide |  | fam_history_q13d_suicide | Biological mother | Parent | baseline |
|  | famhx_ss_matgf_prob_scd_p | Maternal grandfather attempted or committed suicide |  | fam_history_q13e_suicide | Maternal grandfather | Parent | baseline |
|  | famhx_ss_matgm_prob_scd_p | Maternal grandmother attempted or committed suicide |  | fam_history_q13f_suicide | Maternal grandmother | Parent | baseline |
| ABCD Developmental History Questionnaire (dhx01) | devhx_3_p | How old were you/biological mother when the child was born? |  |  |  | Parent | baseline |
|  | devhx_4_p | How old was the child's biological father when he/she was born? |  |  |  | Parent | baseline |
|  | devhx_5_p | Does your child have a twin? |  |  |  | Parent | baseline |
|  | devhx_6_p | Was your pregnancy with this child a planned pregnancy? |  |  |  | Parent | baseline |
|  | devhx_7_p | How far along (in weeks) were you with your child when you found out that you were pregnant? |  |  |  | Parent | baseline |
|  | devhx_8_prescript_med | Before the biological mother/you found out she was pregnant, but while she/you might have been pregnant with this child, did she/you use prescription medications? |  |  |  | Parent | baseline |
| ABCD Developmental History Questionnaire (dhx01) | devhx_8_tobacco | Before the biological mother/you found out she was pregnant, but while she/you might have been pregnant with this child, did she/you use tobacco? |  |  |  | Parent | baseline |
|  | devhx_8_alcohol | Before the biological mother/you found out she was pregnant, but while she/you might have been pregnant with this child, did she/you use alcohol? |  |  |  | Parent | baseline |
|  | devhx_8_marijuana | Before the biological mother/you found out she was pregnant, but while she/you might have been pregnant with this child, did she/you use marijuana? |  |  |  | Parent | baseline |
|  | devhx_8_coc_crack | Before the biological mother/you found out she was pregnant, but while she/you might have been pregnant with this child, did she/you use cocaine/crack? |  |  |  | Parent | baseline |
|  | devhx_8_her_morph | Before the biological mother/you found out she was pregnant, but while she/you might have been pregnant with this child, did she/you use heroin/morphine? |  |  |  | Parent | baseline |
|  | devhx_8_oxycont | Before the biological mother/you found out she was pregnant, but while she/you might have been pregnant with this child, did she/you use oxycontin? |  |  |  | Parent | baseline |
|  | devhx_8_other_drugs | Before the biological mother/you found out she was pregnant, but while she/you might have been pregnant with this child, did she/you use any other drugs? |  |  |  | Parent | baseline |
|  | devhx_9_prescript_med | Once the biological mother/you knew she was pregnant, was she/you using prescription medications? |  |  |  | Parent | baseline |
|  | devhx_9_tobacco | Once the biological mother/you knew she was pregnant, was she/you using tobacco? |  |  |  | Parent | baseline |
|  | devhx_9_alcohol | Once the biological mother/you knew she was pregnant, was she/you using alcohol? |  |  |  | Parent | baseline |
|  | devhx_9_marijuana | Once the biological mother/you knew she was pregnant, was she/you using marijuana? |  |  |  | Parent | baseline |
|  | devhx_9_coc_crack | Once the biological mother/you knew she was pregnant, was she/you using cocaine/crack? |  |  |  | Parent | baseline |
|  | devhx_9_her_morph | Once the biological mother/you knew she was pregnant, was she/you using heroin/morphine? |  |  |  | Parent | baseline |
|  | devhx_9_oxycont | Once the biological mother/you knew she was pregnant, was she/you using oxycontin? |  |  |  | Parent | baseline |
|  | devhx_9_other_drugs | Once the biological mother/you knew she was pregnant, was she/you using any other drugs? |  |  |  | Parent | baseline |
|  | devhx_10 | Did the biological mother/you take prenatal vitamins during the pregnancy? |  |  |  | Parent | baseline |
|  | devhx_caffeine_11 | Did the biological mother/you have any caffeine during pregnancy (from conception until delivery)? |  |  |  | Parent | baseline |
|  | devhx_10a3_p | During the pregnancy with this child, did the biological mother/you have severe nausea and vomiting extending past the 6th month or accompanied by weight loss? |  |  |  | Parent | baseline |
| ABCD Developmental History Questionnaire (dhx01) | devhx_10b3_p | During the pregnancy with this child, did the biological mother/you have heavy bleeding requiring bed rest or special treatment? |  |  |  | Parent | baseline |
|  | devhx_10c3_p | During the pregnancy with this child, did the biological mother/you have pre-eclampsia, eclampsia, or toxemia? |  |  |  | Parent | baseline |
|  | devhx_10d3_p | During the pregnancy with this child, did the biological mother/you have a severe gall bladder attack? |  |  |  | Parent | baseline |
|  | devhx_10e3_p | During the pregnancy with this child, did the biological mother/you have persistent proteinuria? |  |  |  | Parent | baseline |
|  | devhx_10f3_p | During the pregnancy with this child, did the biological mother/you have rubella (German measles) during the first 3 months of pregnancy? |  |  |  | Parent | baseline |
|  | devhx_10g3_p | During the pregnancy with this child, did the biological mother/you have severe anemia? |  |  |  | Parent | baseline |
|  | devhx_10h3_p | During the pregnancy with this child, did the biological mother/you have urinary tract infections? |  |  |  | Parent | baseline |
|  | devhx_10i3_p | During the pregnancy with this child, did the biological mother/you have pregnancy-related diabetes? |  |  |  | Parent | baseline |
|  | devhx_10j3_p | During the pregnancy with this child, did the biological mother/you have pregnancy-related high blood pressure? |  |  |  | Parent | baseline |
|  | devhx_10k3_p | During the pregnancy with this child, did the biological mother/you have previa, abruptio, or other problems with the placenta? |  |  |  | Parent | baseline |
|  | devhx_10l3_p | During the pregnancy with this child, did the biological mother/you have an accident or injury requiring medical care? |  |  |  | Parent | baseline |
|  | devhx_10m3_p | During the pregnancy with this child, did the biological mother/you have any other conditions requiring medical care? |  |  |  | Parent | baseline |
|  | devhx_11_p | About how many times did you/biological mother see a doctor or other medical professional for prenatal care during this pregnancy? |  |  |  | Parent | baseline |
|  | devhx_12a_p | Was the child born prematurely? |  |  |  | Parent | baseline |
|  | devhx_13_3_p | Was the child born by Caesarian section? |  |  |  | Parent | baseline |
|  | devhx_14a3_p | Was the child blue at birth? |  |  |  | Parent | baseline |
|  | devhx_14b3_p | Did the child have a slow heart beat at birth? |  |  |  | Parent | baseline |
|  | devhx_14c3_p | Did the child not breathe at first at birth? |  |  |  | Parent | baseline |
|  | devhx_14d3_p | Did the child have convulsions at birth? |  |  |  | Parent | baseline |
|  | devhx_14e3_p | Did the child have jaundice needing treatment at birth? |  |  |  | Parent | baseline |
|  | devhx_14f3_p | Did the child require oxygen at birth? |  |  |  | Parent | baseline |
|  | devhx_14g3_p | Did the child require a blood transfusion at birth? |  |  |  | Parent | baseline |
|  | devhx_14h3_p | Did the child have Rh incompatibility at birth? |  |  |  | Parent | baseline |
|  | devhx_15 | For how many days after birth was the child in an incubator? |  |  |  | Parent | baseline |
|  | devhx_16_p | About how many days in the first 12 months of life, did the child have a fever of 104 degrees or greater? |  |  |  | Parent | baseline |
| ABCD Developmental History Questionnaire (dhx01) | devhx_17_p | About how many days in the first 12 months of life did the child have any infections or serious illnesses? |  |  |  | Parent | baseline |
|  | devhx_18_p | For how many months was the child breast fed? |  |  |  | Parent | baseline |
|  | devhx_19a_p | At approximately what age was the child FIRST able to roll over? |  |  |  | Parent | baseline |
|  | devhx_19b_p | At approximately what age was the child FIRST able to sit without assistance? |  |  |  | Parent | baseline |
|  | devhx_19c_p | At approximately what age was the child FIRST able to walk without assistance? |  |  |  | Parent | baseline |
|  | devhx_19d_p | At approximately what age was the child FIRST able to say his/her first word? |  |  |  | Parent | baseline |
|  | devhx_20_p | Would you say the child's motor development (sitting, crawling, walking) was earlier, average, or later than most other children? |  |  |  | Parent | baseline |
|  | devhx_21_p | Would you say the child's speech development was earlier, average, or later than most other children? |  |  |  | Parent | baseline |
|  | devhx_22_3_p | Has the child ever wet the bed at night? |  |  |  | Parent | baseline |
| ABCD Parent Diagnostic Interview for DSM-5 (KSADS) Traumatic Events (abcd_ptsd01) | ksads_ptsd_raw_754_p | A car accident in which your child or another person in the car was hurt bad enough to require medical attention |  |  |  | Parent | 1-year follow-up |
|  | ksads_ptsd_raw_755_p | Another significant accident for which your child needed specialized and intensive medical treatment |  |  |  | Parent | 1-year follow-up |
|  | ksads_ptsd_raw_756_p | Witnessed or caught in a fire that caused significant property damage or personal injury |  |  |  | Parent | 1-year follow-up |
|  | ksads_ptsd_raw_757_p | Witnessed or caught in a natural disaster that caused significant property damage or personal injury |  |  |  | Parent | 1-year follow-up |
|  | ksads_ptsd_raw_758_p | Witnessed or present during an act of terrorism (e.g., Boston marathon bombing) |  |  |  | Parent | 1-year follow-up |
|  | ksads_ptsd_raw_759_p | Witnessed death or mass destruction in a war zone |  |  |  | Parent | 1-year follow-up |
|  | ksads_ptsd_raw_760_p | Witnessed someone shot or stabbed in the community |  |  |  | Parent | 1-year follow-up |
|  | ksads_ptsd_raw_761_p | Shot, stabbed, or beaten brutally by a non-family member |  |  |  | Parent | 1-year follow-up |
|  | ksads_ptsd_raw_762_p | Shot, stabbed, or beaten brutally by a grown up in the home |  |  |  | Parent | 1-year follow-up |
|  | ksads_ptsd_raw_763_p | Beaten to the point of having bruises by a grown up in the home |  |  |  | Parent | 1-year follow-up |
|  | ksads_ptsd_raw_764_p | A non-family member threatened to kill your child |  |  |  | Parent | 1-year follow-up |
|  | ksads_ptsd_raw_765_p | A family member threatened to kill your child |  |  |  | Parent | 1-year follow-up |
|  | ksads_ptsd_raw_766_p | Witness the grownups in the home push, shove or hit one another |  |  |  | Parent | 1-year follow-up |
|  | ksads_ptsd_raw_767_p | A grown up in the home touched your child in his or her privates, had your child touch their privates, or did other sexual things to your child |  |  |  | Parent | 1-year follow-up |
| ABCD Parent Diagnostic Interview for DSM-5 (KSADS) Traumatic Events (abcd_ptsd01) | ksads_ptsd_raw_768_p | An adult outside your family touched your child in his or her privates, had your child touch their privates or did other sexual things to your child |  |  |  | Parent | 1-year follow-up |
|  | ksads_ptsd_raw_769_p | A peer forced your child to do something sexually |  |  |  | Parent | 1-year follow-up |
|  | ksads_ptsd_raw_770_p | Learned about the sudden unexpected death of a loved one |  |  |  | Parent | 1-year follow-up |
| Residential History Derived Scores (abcd_rhds01) | reshist_addr1_valid | Residential history validity |  |  |  | Address-derived | baseline |
|  | reshist_addr1_status | Residential history status message |  |  |  | Address-derived | baseline |
|  | reshist_addr1_years | Residential history years of residence |  |  |  | Address-derived | baseline |
|  | reshist_addr1_d1a | Gross residential density |  |  |  | Address-derived | baseline |
|  | reshist_addr1_walkindex | National walkability index |  |  |  | Address-derived | baseline |
|  | reshist_addr1_grndtot | Uniform Crime Reports: grand total crimes |  |  |  | Address-derived | baseline |
|  | reshist_addr1_p1tot | Uniform Crime Reports: total adult offenses |  |  |  | Address-derived | baseline |
|  | reshist_addr1_p1vlnt | Uniform Crime Reports: adult violent crimes |  |  |  | Address-derived | baseline |
|  | reshist_addr1_drugtot | Uniform Crime Reports: drug abuse violations total |  |  |  | Address-derived | baseline |
|  | reshist_addr1_drgsale | Uniform Crime Reports: drug sale total |  |  |  | Address-derived | baseline |
|  | reshist_addr1_mjsale | Uniform Crime Reports: marijuana sale total |  |  |  | Address-derived | baseline |
|  | reshist_addr1_drgposs | Uniform Crime Reports: drug possession total |  |  |  | Address-derived | baseline |
|  | reshist_addr1_dui | Uniform Crime Reports: DUI |  |  |  | Address-derived | baseline |
|  | reshist_addr1_adi_edu_l | Area Deprivation Index: percentage of population aged at least 25 with less than 9 years of education |  |  |  | Address-derived | baseline |
|  | reshist_addr1_adi_edu_h | Area Deprivation Index: percentage of population aged at least 25 with at least a high school diploma |  |  |  | Address-derived | baseline |
|  | reshist_addr1_adi_work_c | Area Deprivation Index: percentage of employed persons aged at least 16 in white collar occupations |  |  |  | Address-derived | baseline |
|  | reshist_addr1_adi_income | Area Deprivation Index: median family income |  |  |  | Address-derived | baseline |
|  | reshist_addr1_adi_in_dis | Area Deprivation Index: income disparity (defined by Singh as the log of 100 x ratio of the number of households with <10,000 annual income to the number of households with >50,000 annual income) |  |  |  | Address-derived | baseline |
|  | reshist_addr1_adi_home_v | Area Deprivation Index: median home value |  |  |  | Address-derived | baseline |
|  | reshist_addr1_adi_rent | Area Deprivation Index: median gross rent |  |  |  | Address-derived | baseline |
|  | reshist_addr1_adi_mortg | Area Deprivation Index: median monthly mortgage |  |  |  | Address-derived | baseline |
| Residential History Derived Scores (abcd_rhds01) | reshist_addr1_adi_home_o | Area Deprivation Index: percentage of house/residence ownership |  |  |  | Address-derived | baseline |
|  | reshist_addr1_adi_crowd | Area Deprivation Index: percentage of occupied housing units with more than 1 person per room (crowding) |  |  |  | Address-derived | baseline |
|  | reshist_addr1_adi_unemp | Area Deprivation Index: percentage of civilian labor force population aged at least 16 unemployed (unemployment rate) |  |  |  | Address-derived | baseline |
|  | reshist_addr1_adi_pov | Area Deprivation Index: percentage of families below the poverty level |  |  |  | Address-derived | baseline |
|  | reshist_addr1_adi_b138 | Area Deprivation Index: percentage of population below 138% of the poverty threshold |  |  |  | Address-derived | baseline |
|  | reshist_addr1_adi_sp | Area Deprivation Index: percentage of single households |  |  |  | Address-derived | baseline |
|  | reshist_addr1_adi_ncar | Area Deprivation Index: percentage of occupied housing units without a motor vehicle |  |  |  | Address-derived | baseline |
|  | reshist_addr1_adi_ntel | Area Deprivation Index: percentage of occupied housing units without a telephone |  |  |  | Address-derived | baseline |
|  | reshist_addr1_adi_nplumb | Area Deprivation Index: percentage of occupied housing units without complete plumbing |  |  |  | Address-derived | baseline |
|  | reshist_addr1_adi_wsum | Area Deprivation Index: scaled weighted sum (based on Kind et al., Annals of Internal Medicine, 2014) |  |  |  | Address-derived | baseline |
|  | reshist_addr1_adi_perc | Area Deprivation Index: national percentiles (higher means higher value of ADI) |  |  |  | Address-derived | baseline |
|  | reshist_addr1_popdensity | UN adjusted population density |  |  |  | Address-derived | baseline |
|  | reshist_addr1_no2 | 3 years average of ground level NO2 at 10x10km2 |  |  |  | Address-derived | baseline |
|  | reshist_addr1_pm25 | Annual average of PM 2.5 at 10x10km2 |  |  |  | Address-derived | baseline |
|  | reshist_addr1_proxrd | Proximity to major roads (in meters) |  |  |  | Address-derived | baseline |
|  | reshist_addr1_percentile | Percentage of time spent at primary residential address |  |  |  | Address-derived | baseline |
|  | reshist_addr1_pm252016aa | Annual average of PM 2.5 at 1x1km2 |  |  |  | Address-derived | baseline |
|  | reshist_addr1_leadrisk_poverty | Percentage of individuals below -125% of the poverty threshold |  |  |  | Address-derived | baseline |
|  | reshist_addr1_leadrisk_housing | Estimated percentage of homes at risk for lead exposure given lead-based paint |  |  |  | Address-derived | baseline |
|  | reshist_addr1_leadrisk | Estimated lead risk (1-10 scale) |  |  |  | Address-derived | baseline |
|  | reshist_state_sexism_factor | State level indicators of sexism from survey and implicit bias measures |  |  |  | Address-derived | baseline |
|  | reshist_state_racism_factor | State level indicators of racism from survey and implicit bias measures and state level structural variables |  |  |  | Address-derived | baseline |
|  | reshist_state_so_factor | State level indicators of bias against sexual orientation from structural variables |  |  |  | Address-derived | baseline |
|  | reshist_state_immigrant_factor | State level indicators of immigrant bias from survey and implicit bias measures and state level structural variables |  |  |  | Address-derived | baseline |
| Residential History Derived Scores (abcd_rhds01) | reshist_state_mj_laws | Marijuana state law during the same year as the assessment |  |  |  | Address-derived | baseline |
| ABCD Youth Life Events (abcd_yle01) | ple_died_y | Someone in family died |  |  |  | Youth | 1-year follow-up |
|  | ple_injured_y | Family member was seriously injured |  |  |  | Youth | 1-year follow-up |
|  | ple_crime_y | Saw crime or accident |  |  |  | Youth | 1-year follow-up |
|  | ple_friend_y | Lost a close friend |  |  |  | Youth | 1-year follow-up |
|  | ple_friend_injur_y | Close friend was seriously sick/injured |  |  |  | Youth | 1-year follow-up |
|  | ple_financial_y | Negative change in parent's financial situation |  |  |  | Youth | 1-year follow-up |
|  | ple_sud_y | Family member had drug and/or alcohol problem |  |  |  | Youth | 1-year follow-up |
|  | ple_ill_y | You got seriously sick |  |  |  | Youth | 1-year follow-up |
|  | ple_injur_y | You got seriously injured |  |  |  | Youth | 1-year follow-up |
|  | ple_argue_y | Parents argued more than previously |  |  |  | Youth | 1-year follow-up |
|  | ple_job_y | Mother/father figure lost job |  |  |  | Youth | 1-year follow-up |
|  | ple_away_y | One parent was away from home more often |  |  |  | Youth | 1-year follow-up |
|  | ple_arrest_y | Someone in the family was arrested |  |  |  | Youth | 1-year follow-up |
|  | ple_friend_died_y | Close friend died |  |  |  | Youth | 1-year follow-up |
|  | ple_mh_y | Family member had mental/emotional problem |  |  |  | Youth | 1-year follow-up |
|  | ple_sib_y | Brother or sister left home |  |  |  | Youth | 1-year follow-up |
|  | ple_victim_y | Was a victim of crime/violence/assault |  |  |  | Youth | 1-year follow-up |
|  | ple_separ_y | Parents separated or divorced |  |  |  | Youth | 1-year follow-up |
|  | ple_law_y | Parents/caregiver got into trouble with the law |  |  |  | Youth | 1-year follow-up |
|  | ple_school_y | Attended a new school |  |  |  | Youth | 1-year follow-up |
|  | ple_move_y | Family moved |  |  |  | Youth | 1-year follow-up |
|  | ple_jail_y | One of the parents/caregivers went to jail |  |  |  | Youth | 1-year follow-up |
|  | ple_step_y | Got new stepmother or stepfather |  |  |  | Youth | 1-year follow-up |
|  | ple_new_job_y | Parent/caregiver got a new job |  |  |  | Youth | 1-year follow-up |
|  | ple_new_sib_y | Got new brother or sister |  |  |  | Youth | 1-year follow-up |
|  |  |  |  |  |  |  |  |
| Sum Scores Mental Health Youth (abcd_mhy02) | ple_y_ss_total_number | Total number of events | abcd_yle01 | ple_died_y | Someone in family died | Youth | 1-year follow-up |
|  |  |  |  | ple_injured_y | Family member was seriously injured | Youth | 1-year follow-up |
|  |  |  |  | ple_crime_y | Saw crime or accident | Youth | 1-year follow-up |
|  |  |  |  | ple_friend_y | Lost a close friend | Youth | 1-year follow-up |
|  |  |  |  | ple_friend_injur_y | Close friend was seriously sick/injured | Youth | 1-year follow-up |
|  |  |  |  | ple_financial_y | Negative change in parent's financial situation | Youth | 1-year follow-up |
|  |  |  |  | ple_sud_y | Family member had drug and/or alcohol problem | Youth | 1-year follow-up |
|  |  |  |  | ple_ill_y | You got seriously sick | Youth | 1-year follow-up |
|  |  |  |  | ple_injur_y | You got seriously injured | Youth | 1-year follow-up |
|  |  |  |  | ple_argue_y | Parents argued more than previously | Youth | 1-year follow-up |
|  |  |  |  | ple_job_y | Mother/father figure lost job | Youth | 1-year follow-up |
|  |  |  |  | ple_away_y | One parent was away from home more often | Youth | 1-year follow-up |
|  |  |  |  | ple_arrest_y | Someone in the family was arrested | Youth | 1-year follow-up |
|  |  |  |  | ple_friend_died_y | Close friend died | Youth | 1-year follow-up |
|  |  |  |  | ple_mh_y | Family member had mental/emotional problem | Youth | 1-year follow-up |
|  |  |  |  | ple_sib_y | Brother or sister left home | Youth | 1-year follow-up |
|  |  |  |  | ple_victim_y | Was a victim of crime/violence/assault | Youth | 1-year follow-up |
|  |  |  |  | ple_separ_y | Parents separated or divorced | Youth | 1-year follow-up |
|  |  |  |  | ple_law_y | Parents/caregiver got into trouble with the law | Youth | 1-year follow-up |
|  |  |  |  | ple_school_y | Attended a new school | Youth | 1-year follow-up |
|  |  |  |  | ple_move_y | Family moved | Youth | 1-year follow-up |
|  |  |  |  | ple_jail_y | One of the parents/caregivers went to jail | Youth | 1-year follow-up |
|  |  |  |  | ple_step_y | Got new stepmother or stepfather | Youth | 1-year follow-up |
|  |  |  |  | ple_new_job_y | Parent/caregiver got a new job | Youth | 1-year follow-up |
|  |  |  |  | ple_new_sib_y | Got new brother or sister | Youth | 1-year follow-up |
|  | ple_y_ss_total_good | Total number of good events |  | ple_died_fu_y | Was this a good or bad experience? | Youth | 1-year follow-up |
|  |  |  |  | ple_injured_fu_y | Was this a good or bad experience? | Youth | 1-year follow-up |
|  |  |  |  | ple_crime_fu_y | Was this a good or bad experience? | Youth | 1-year follow-up |
| Sum Scores Mental Health Youth (abcd_mhy02) | ple_y_ss_total_good | Total number of good events | abcd_yle01 | ple_friend_fu_y | Was this a good or bad experience? | Youth | 1-year follow-up |
|  |  |  |  | ple_friend_injur_fu_y | Was this a good or bad experience? | Youth | 1-year follow-up |
|  |  |  |  | ple_financial_fu_y | Was this a good or bad experience? | Youth | 1-year follow-up |
|  |  |  |  | ple_sud_fu_y | Was this a good or bad experience? | Youth | 1-year follow-up |
|  |  |  |  | ple_ill_fu_y | Was this a good or bad experience? | Youth | 1-year follow-up |
|  |  |  |  | ple_injur_fu_y | Was this a good or bad experience? | Youth | 1-year follow-up |
|  |  |  |  | ple_argue_fu_y | Was this a good or bad experience? | Youth | 1-year follow-up |
|  |  |  |  | ple_job_fu_y | Was this a good or bad experience? | Youth | 1-year follow-up |
|  |  |  |  | ple_away_fu_y | Was this a good or bad experience? | Youth | 1-year follow-up |
|  |  |  |  | ple_arrest_fu_y | Was this a good or bad experience? | Youth | 1-year follow-up |
|  | ple_y_ss_total_bad | Total number of bad events |  | ple_friend_died_fu_y | Was this a good or bad experience? | Youth | 1-year follow-up |
|  |  |  |  | ple_mh_fu_y | Was this a good or bad experience? | Youth | 1-year follow-up |
|  |  |  |  | ple_sib_fu_y | Was this a good or bad experience? | Youth | 1-year follow-up |
|  |  |  |  | ple_victim_fu_y | Was this a good or bad experience? | Youth | 1-year follow-up |
|  |  |  |  | ple_separ_fu_y | Was this a good or bad experience? | Youth | 1-year follow-up |
|  |  |  |  | ple_law_fu_y | Was this a good or bad experience? | Youth | 1-year follow-up |
|  |  |  |  | ple_school_fu_y | Was this a good or bad experience? | Youth | 1-year follow-up |
|  |  |  |  | ple_move_fu_y | Was this a good or bad experience? | Youth | 1-year follow-up |
|  |  |  |  | ple_jail_fu_y | Was this a good or bad experience? | Youth | 1-year follow-up |
|  |  |  |  | ple_step_fu_y | Was this a good or bad experience? | Youth | 1-year follow-up |
|  |  |  |  | ple_new_job_fu_y | Was this a good or bad experience? | Youth | 1-year follow-up |
|  |  |  |  | ple_new_sib_fu_y | Was this a good or bad experience? | Youth | 1-year follow-up |
|  | ple_y_ss_affect_sum | How much affected (sum) |  | ple_died_fu2_y | How much did the event affect you? | Youth | 1-year follow-up |
|  |  |  |  | ple_injured_fu2_y | How much did the event affect you? | Youth | 1-year follow-up |
|  |  |  |  | ple_crime_fu2_y | How much did the event affect you? | Youth | 1-year follow-up |
|  |  |  |  | ple_friend_fu2_y | How much did the event affect you? | Youth | 1-year follow-up |
|  |  |  |  | ple_friend_injur_fu2_y | How much did the event affect you? | Youth | 1-year follow-up |
|  |  |  |  | ple_financial_fu2_y | How much did the event affect you? | Youth | 1-year follow-up |
| Sum Scores Mental Health Youth (abcd_mhy02) | ple_y_ss_affect_sum | How much affected (sum) | abcd_yle01 | ple_sud_fu2_y | How much did the event affect you? | Youth | 1-year follow-up |
|  | ple_y_ss_affected_good_sum | How much affected (good) (sum) |  | ple_ill_fu2_y | How much did the event affect you? | Youth | 1-year follow-up |
|  |  |  |  | ple_injur_fu2_y | How much did the event affect you? | Youth | 1-year follow-up |
|  |  |  |  | ple_argue_fu2_y | How much did the event affect you? | Youth | 1-year follow-up |
|  |  |  |  | ple_job_fu2_y | How much did the event affect you? | Youth | 1-year follow-up |
|  |  |  |  | ple_away_fu2_y | How much did the event affect you? | Youth | 1-year follow-up |
|  |  |  |  | ple_arrest_fu2_y | How much did the event affect you? | Youth | 1-year follow-up |
|  | ple_y_ss_affected_bad_sum | How much affected (bad) (sum) |  | ple_friend_died_fu2_y | How much did the event affect you? | Youth | 1-year follow-up |
|  |  |  |  | ple_mh_fu2_y | How much did the event affect you? | Youth | 1-year follow-up |
|  |  |  |  | ple_sib_fu2_y | How much did the event affect you? | Youth | 1-year follow-up |
|  |  |  |  | ple_victim_fu2_y | How much did the event affect you? | Youth | 1-year follow-up |
|  |  |  |  | ple_separ_fu2_y | How much did the event affect you? | Youth | 1-year follow-up |
|  |  |  |  | ple_law_fu2_y | How much did the event affect you? | Youth | 1-year follow-up |
|  | ple_y_ss_affected_bad_mean | How much affected (bad) (mean) |  | ple_school_fu2_y | How much did the event affect you? | Youth | 1-year follow-up |
|  |  |  |  | ple_move_fu2_y | How much did the event affect you? | Youth | 1-year follow-up |
|  |  |  |  | ple_jail_fu2_y | How much did the event affect you? | Youth | 1-year follow-up |
|  |  |  |  | ple_step_fu2_y | How much did the event affect you? | Youth | 1-year follow-up |
|  |  |  |  | ple_new_job_fu2_y | How much did the event affect you? | Youth | 1-year follow-up |
|  |  |  |  | ple_new_sib_fu2_y | How much did the event affect you? | Youth | 1-year follow-up |
| Sum Scores Mental Health Parent (abcd_mhp02) | ple_p_ss_total_number | Total number of events | abcd_ple01 | ple_died_p | Someone in family died | Parent | 1-year follow-up |
|  |  |  |  | ple_injured_p | Family member was seriously injured | Parent | 1-year follow-up |
|  |  |  |  | ple_crime_p | Saw crime or accident | Parent | 1-year follow-up |
|  |  |  |  | ple_friend_p | Lost a close friend | Parent | 1-year follow-up |
|  |  |  |  | ple_friend_injur_p | Close friend was seriously sick/injured | Parent | 1-year follow-up |
|  |  |  |  | ple_financial_p | Negative change in parent's financial situation | Parent | 1-year follow-up |
|  |  |  |  | ple_sud_p | Family member had drug and/or alcohol problem | Parent | 1-year follow-up |
|  |  |  |  | ple_ill_p | You got seriously sick | Parent | 1-year follow-up |
|  |  |  |  | ple_injur_p | You got seriously injured | Parent | 1-year follow-up |
| Sum Scores Mental Health Parent (abcd_mhp02) | ple_p_ss_total_number | Total number of events | abcd_ple01 | ple_argue_p | Parents argued more than previously | Parent | 1-year follow-up |
|  |  |  |  | ple_job_p | Mother/father figure lost job | Parent | 1-year follow-up |
|  |  |  |  | ple_away_p | One parent was away from home more often | Parent | 1-year follow-up |
|  |  |  |  | ple_arrest_p | Someone in the family was arrested | Parent | 1-year follow-up |
|  |  |  |  | ple_friend_died_p | Close friend died | Parent | 1-year follow-up |
|  |  |  |  | ple_mh_p | Family member had mental/emotional problem | Parent | 1-year follow-up |
|  |  |  |  | ple_sib_p | Brother or sister left home | Parent | 1-year follow-up |
|  |  |  |  | ple_victim_p | Was a victim of crime/violence/assault | Parent | 1-year follow-up |
|  |  |  |  | ple_separ_p | Parents separated or divorced | Parent | 1-year follow-up |
|  |  |  |  | ple_law_p | Parents/caregiver got into trouble with the law | Parent | 1-year follow-up |
|  |  |  |  | ple_school_p | Attended a new school | Parent | 1-year follow-up |
|  |  |  |  | ple_move_p | Family moved | Parent | 1-year follow-up |
|  |  |  |  | ple_jail_p | One of the parents/caregivers went to jail | Parent | 1-year follow-up |
|  |  |  |  | ple_step_p | Got new stepmother or stepfather | Parent | 1-year follow-up |
|  |  |  |  | ple_new_job_p | Parent/caregiver got a new job | Parent | 1-year follow-up |
|  |  |  |  | ple_new_sib_p | Got new brother or sister | Parent | 1-year follow-up |
|  | ple_p_ss_total_good | Total number of good events |  | ple_died_fu_p | Was this a good or bad experience for your child? | Parent | 1-year follow-up |
|  |  |  |  | ple_injured_fu_p | Was this a good or bad experience for your child? | Parent | 1-year follow-up |
|  |  |  |  | ple_crime_fu_p | Was this a good or bad experience for your child? | Parent | 1-year follow-up |
|  |  |  |  | ple_friend_fu_p | Was this a good or bad experience for your child? | Parent | 1-year follow-up |
|  |  |  |  | ple_friend_injur_fu_p | Was this a good or bad experience for your child? | Parent | 1-year follow-up |
|  |  |  |  | ple_financial_fu_p | Was this a good or bad experience for your child? | Parent | 1-year follow-up |
|  |  |  |  | ple_sud_fu_p | Was this a good or bad experience for your child? | Parent | 1-year follow-up |
|  |  |  |  | ple_ill_fu_p | Was this a good or bad experience for your child? | Parent | 1-year follow-up |
|  |  |  |  | ple_injur_fu_p | Was this a good or bad experience for your child? | Parent | 1-year follow-up |
|  |  |  |  | ple_argue_fu_p | Was this a good or bad experience for your child? | Parent | 1-year follow-up |
|  |  |  |  | ple_job_fu_p | Was this a good or bad experience for your child? | Parent | 1-year follow-up |
|  |  |  |  | ple_away_fu_p | Was this a good or bad experience for your child? | Parent | 1-year follow-up |
| Sum Scores Mental Health Parent (abcd_mhp02) | ple_p_ss_total_good | Total number of good events | abcd_ple01 | ple_arrest_fu_p | Was this a good or bad experience for your child? | Parent | 1-year follow-up |
|  | ple_p_ss_total_bad | Total number of bad events |  | ple_friend_died_fu_p | Was this a good or bad experience for your child? | Parent | 1-year follow-up |
|  |  |  |  | ple_mh_fu_p | Was this a good or bad experience for your child? | Parent | 1-year follow-up |
|  |  |  |  | ple_sib_fu_p | Was this a good or bad experience for your child? | Parent | 1-year follow-up |
|  |  |  |  | ple_victim_fu_p | Was this a good or bad experience for your child? | Parent | 1-year follow-up |
|  |  |  |  | ple_separ_fu_p | Was this a good or bad experience for your child? | Parent | 1-year follow-up |
|  |  |  |  | ple_law_fu_p | Was this a good or bad experience for your child? | Parent | 1-year follow-up |
|  |  |  |  | ple_school_fu_p | Was this a good or bad experience for your child? | Parent | 1-year follow-up |
|  |  |  |  | ple_move_fu_p | Was this a good or bad experience for your child? | Parent | 1-year follow-up |
|  |  |  |  | ple_jail_fu_p | Was this a good or bad experience for your child? | Parent | 1-year follow-up |
|  |  |  |  | ple_step_fu_p | Was this a good or bad experience for your child? | Parent | 1-year follow-up |
|  |  |  |  | ple_new_job_fu_p | Was this a good or bad experience for your child? | Parent | 1-year follow-up |
|  |  |  |  | ple_new_sib_fu_p | Was this a good or bad experience for your child? | Parent | 1-year follow-up |
|  | ple_p_ss_affected_good_sum | How much affected (good) (sum) |  | ple_died_fu2_p | How much did the event affect your child? | Parent | 1-year follow-up |
|  |  |  |  | ple_injured_fu2_p | How much did the event affect your child? | Parent | 1-year follow-up |
|  |  |  |  | ple_crime_fu2_p | How much did the event affect your child? | Parent | 1-year follow-up |
|  |  |  |  | ple_friend_fu2_p | How much did the event affect your child? | Parent | 1-year follow-up |
|  |  |  |  | ple_friend_injur_fu2_p | How much did the event affect your child? | Parent | 1-year follow-up |
|  |  |  |  | ple_financial_fu2_p | How much did the event affect your child? | Parent | 1-year follow-up |
|  |  |  |  | ple_sud_fu2_p | How much did the event affect your child? | Parent | 1-year follow-up |
|  | ple_p_ss_affected_bad_sum | How much affected (bad) (sum) |  | ple_ill_fu2_p | How much did the event affect your child? | Parent | 1-year follow-up |
|  |  |  |  | ple_injur_fu2_p | How much did the event affect your child? | Parent | 1-year follow-up |
|  |  |  |  | ple_argue_fu2_p | How much did the event affect your child? | Parent | 1-year follow-up |
|  |  |  |  | ple_job_fu2_p | How much did the event affect your child? | Parent | 1-year follow-up |
|  |  |  |  | ple_away_fu2_p | How much did the event affect your child? | Parent | 1-year follow-up |
|  |  |  |  | ple_arrest_fu2_p | How much did the event affect your child? | Parent | 1-year follow-up |
|  | ple_p_ss_affected_mean | How much affected (bad) (mean) |  | ple_friend_died_fu2_p | How much did the event affect your child? | Parent | 1-year follow-up |
|  |  |  |  | ple_mh_fu2_p | How much did the event affect your child? | Parent | 1-year follow-up |
| Sum Scores Mental Health Parent (abcd_mhp02) | ple_p_ss_affected_mean | How much affected (bad) (mean) | abcd_ple01 | ple_sib_fu2_p | How much did the event affect your child? | Parent | 1-year follow-up |
|  |  |  |  | ple_victim_fu2_p | How much did the event affect your child? | Parent | 1-year follow-up |
|  |  |  |  | ple_separ_fu2_p | How much did the event affect your child? | Parent | 1-year follow-up |
|  |  |  |  | ple_law_fu2_p | How much did the event affect your child? | Parent | 1-year follow-up |
|  | ple_p_ss_affect_sum | How much affected (sum) |  | ple_school_fu2_p | How much did the event affect your child? | Parent | 1-year follow-up |
|  |  |  |  | ple_move_fu2_p | How much did the event affect your child? | Parent | 1-year follow-up |
|  |  |  |  | ple_jail_fu2_p | How much did the event affect your child? | Parent | 1-year follow-up |
|  |  |  |  | ple_step_fu2_p | How much did the event affect your child? | Parent | 1-year follow-up |
|  |  |  |  | ple_new_job_fu2_p | How much did the event affect your child? | Parent | 1-year follow-up |
|  |  |  |  | ple_new_sib_fu2_p | How much did the event affect your child? | Parent | 1-year follow-up |
| ABCD Longitudinal Parent Demographics Survey (abcd_lpds01) | demo_fam_exp1_v2_l | Needed food but couldn't afford to buy it or couldn't afford to go out to get it |  |  |  | Parent | 1-year follow-up |
|  | demo_fam_exp2_v2_l | Were without telephone service because you could not afford it |  |  |  | Parent | 1-year follow-up |
|  | demo_fam_exp3_v2_l | Didn't pay the full amount of the rent or mortgage because you could not afford it |  |  |  | Parent | 1-year follow-up |
|  | demo_fam_exp4_v2_l | Were evicted from your home for not paying the rent or mortgage |  |  |  | Parent | 1-year follow-up |
|  | demo_fam_exp5_v2_l | Had services turned off by the gas or electric company, or the oil company wouldn't deliver oil because payments were not made |  |  |  | Parent | 1-year follow-up |
|  | demo_fam_exp6_v2_l | Had someone who needed to see a doctor or go to the hospital but didn't go because you could not afford it |  |  |  | Parent | 1-year follow-up |
|  | demo_fam_exp7_v2_l | Had someone who needed a dentist but couldn't go because you could not afford it |  |  |  | Parent | 1-year follow-up |
|  | demo_yrs_1_l | How often does your child attend religious services? |  |  |  | Parent | 1-year follow-up |
|  | demo_yrs_2_l | In general, how important are your child's religious and spiritual beliefs in his/her daily life? |  |  |  | Parent | 1-year follow-up |

The full range of ABCD exposome variables used in factor analysis and generation. The ABCD instrument and item descriptions for each of the 348 measures that went directly into exploratory factor analyses are displayed in the leftmost three columns. Some of these measures were ABCD-derived summary variables; in these cases, the constituent instruments and items are described in the subsequent three columns. For every item, reporter and time point are displayed as well. Measures relating to genetics, neuroimaging, psychological state, and substance use were omitted.

**Supplemental Table 4.** Full set of psychopathology measures included in ABCD Study analysis

| **ABCD instrument** | **ABCD item code** | **ABCD item description** | **Reporter** | **Time point** |
| --- | --- | --- | --- | --- |
| ABCD Parent Diagnostic Interview for DSM-5 Full (KSADS-5) (abcd_ksad01) | ksads_14_77_p | Difficulty sustaining attention since elementary school (Present) | Parent | 1-year follow-up |
|  | ksads_14_79_p | Difficulty sustaining attention for more than one school year (Past) | Parent | 1-year follow-up |
|  | ksads_14_81_p | Easily distracted since elementary school (Present) | Parent | 1-year follow-up |
|  | ksads_14_83_p | Easily distracted for more than one school year (Past) | Parent | 1-year follow-up |
|  | ksads_14_85_p | Difficulty remaining seated since elementary school (Present) | Parent | 1-year follow-up |
|  | ksads_14_87_p | Difficulty remaining seated for more than one school year (Past) | Parent | 1-year follow-up |
|  | ksads_14_88_p | Impulsivity (Present) | Parent | 1-year follow-up |
|  | ksads_14_90_p | Impulsivity for more than one school year (Past) | Parent | 1-year follow-up |
|  | ksads_15_95_p | Often disobeys rules/requests (Present) | Parent | 1-year follow-up |
|  | ksads_15_96_p | Often disobeys rules/requests (Past) | Parent | 1-year follow-up |
|  | ksads_15_94_p | Often argues with adults/authority (Past) | Parent | 1-year follow-up |
|  | ksads_15_93_p | Often argues with adults/authority (Present) | Parent | 1-year follow-up |
|  | ksads_16_98_p | Often lies (Present) | Parent | 1-year follow-up |
|  | ksads_16_99_p | Often lies (Past) | Parent | 1-year follow-up |
|  | ksads_16_104_p | Often bullies others (Present) | Parent | 1-year follow-up |
|  | ksads_16_105_p | Often bullies others (Past) | Parent | 1-year follow-up |
|  | ksads_16_107_p | Stealing (Past) | Parent | 1-year follow-up |
|  | ksads_16_106_p | Stealing (Present) | Parent | 1-year follow-up |
| ABCD Prodromal Psychosis Scale (pps01) | prodromal_1_y | Did places that you know well, such as your bedroom, or other rooms in your home, your classroom or school yard, suddenly seem weird, strange or confusing to you; like not the real world? | Youth | 1-year follow-up |
|  | prodromal_2_y | Did you hear strange sounds that you never noticed before like banging, clicking, hissing, clapping, or ringing in your ears? | Youth | 1-year follow-up |
| ABCD Prodromal Psychosis Scale (pps01) | prodromal_3_y | Do things that you see appear different from the way they usually do (brighter or duller, larger or smaller, or changed in some other way)? | Youth | 1-year follow-up |
|  | prodromal_4_y | Did you feel like you had special, unusual powers like you could make things happen by magic, or that you could magically know what was inside another person's mind, or magically know what was going to happen in the future when other people could not? | Youth | 1-year follow-up |
|  | prodromal_5_y | Did you feel that someone else, who is not you, has taken control over the private, personal, thoughts or ideas inside your head? | Youth | 1-year follow-up |
|  | prodromal_6_y | Did you suddenly find it hard to figure out how to say something quickly and easily so that other people would understand what you meant? | Youth | 1-year follow-up |
|  | prodromal_7_y | Did you ever feel very certain that you have very special abilities or magical talents that other people do not have? | Youth | 1-year follow-up |
|  | prodromal_8_y | Did you suddenly feel that you could not trust other people because they seemed to be watching you or talking about you in an unfriendly way? | Youth | 1-year follow-up |
|  | prodromal_9_y | Do you sometimes get strange feelings on or just beneath your skin, like bugs crawling? | Youth | 1-year follow-up |
|  | prodromal_10_y | Did you lose concentration because you noticed sounds in the distance that you usually don't hear? | Youth | 1-year follow-up |
|  | prodromal_11_y | Although you could not see anything or anyone, did you suddenly start to feel that an invisible energy, creature, or some person was around you? | Youth | 1-year follow-up |
|  | prodromal_12_y | Did you start to worry at times that your mind was trying to trick you or was not working right? | Youth | 1-year follow-up |
|  | prodromal_13_y | Did you feel that the world is not real, you are not real, or that you are dead? | Youth | 1-year follow-up |
|  | prodromal_14_y | Did you feel confused because something you experienced didn't seem real or it seemed imaginary to you? | Youth | 1-year follow-up |
| ABCD Prodromal Psychosis Scale (pps01) | prodromal_15_y | Did you honestly believe in things that other people would say are unusual or weird? | Youth | 1-year follow-up |
|  | prodromal_16_y | Did you feel that parts of your body had suddenly changed or worked differently than before; like your legs had suddenly turned to something else or your nose could suddenly smell things you'd never actually smelled before? | Youth | 1-year follow-up |
|  | prodromal_17_y | Did you feel that sometimes your thoughts were so strong you could almost hear them, as if another person, NOT you, spoke them? | Youth | 1-year follow-up |
|  | prodromal_18_y | Did you feel that other people might want something bad to happen to you or that you could not trust other people? | Youth | 1-year follow-up |
|  | prodromal_19_y | Did you suddenly start to see unusual things that you never saw before like flashes, flames, blinding light, or shapes floating in front of you? | Youth | 1-year follow-up |
|  | prodromal_20_y | Have you seen things that other people can't see or don't seem to see? | Youth | 1-year follow-up |
|  | prodromal_21_y | Did you suddenly start to notice that people sometimes had a hard time understanding what you were saying, even though they used to understand you well? | Youth | 1-year follow-up |
| ABCD Youth Brief Problem Monitor (abcd_bpm01) | bpm_1_y | "I act too young for my age" | Youth | 1-year follow-up |
|  | bpm_2_y | "I argue a lot" | Youth | 1-year follow-up |
|  | bpm_3_y | "I fail to finish things I start" | Youth | 1-year follow-up |
|  | bpm_4_y | "I have trouble concentrating or paying attention" | Youth | 1-year follow-up |
|  | bpm_5_y | "I have trouble sitting still" | Youth | 1-year follow-up |
|  | bpm_6_y | "I destroy things belonging to others" | Youth | 1-year follow-up |
|  | bpm_7_y | "I disobey my parents" | Youth | 1-year follow-up |
|  | bpm_8_y | "I disobey at school" | Youth | 1-year follow-up |
|  | bpm_9_y | "I feel worthless or inferior" | Youth | 1-year follow-up |
|  | bpm_10_y | "I act without stopping to think" | Youth | 1-year follow-up |
|  | bpm_11_y | "I am too fearful or anxious" | Youth | 1-year follow-up |
|  | bpm_12_y | "I feel too guilty" | Youth | 1-year follow-up |
| ABCD Youth Brief Problem Monitor (abcd_bpm01) | bpm_13_y | "I am self-conscious or easily embarrassed" | Youth | 1-year follow-up |
|  | bpm_14_y | "I am inattentive or easily distracted" | Youth | 1-year follow-up |
|  | bpm_15_y | "I am stubborn" | Youth | 1-year follow-up |
|  | bpm_16_y | "I have a hot temper" | Youth | 1-year follow-up |
|  | bpm_17_y | "I threaten to hurt people" | Youth | 1-year follow-up |
|  | bpm_18_y | "I am unhappy, sad, or depressed" | Youth | 1-year follow-up |
|  | bpm_19_y | "I worry a lot" | Youth | 1-year follow-up |
| ABCD Youth NIH Toolbox Positive Affect Items (abcd_ytbpai01) | poa_nihtb_1_y | "I felt attentive" | Youth | 1-year follow-up |
|  | poa_nihtb_2_y | "I felt delighted" | Youth | 1-year follow-up |
|  | poa_nihtb_3_y | "I felt calm" | Youth | 1-year follow-up |
|  | poa_nihtb_4_y | "I felt at ease" | Youth | 1-year follow-up |
|  | poa_nihtb_5_y | "I felt enthusiastic" | Youth | 1-year follow-up |
|  | poa_nihtb_6_y | "I felt interested" | Youth | 1-year follow-up |
|  | poa_nihtb_7_y | "I felt confident" | Youth | 1-year follow-up |
|  | poa_nihtb_8_y | "I felt energetic" | Youth | 1-year follow-up |
|  | poa_nihtb_9_y | "I felt able to concentrate" | Youth | 1-year follow-up |
| ABCD Youth Diagnostic Interview for DSM-5 5 (KSADS-5) (abcd_ksad501) | ksads_23_945_t | Self-injurious behavior without suicidal intent (Present) | Youth | 1-year follow-up |
|  | ksads_23_946_t | Suicidal ideation (Passive) (Present) | Youth | 1-year follow-up |
|  | ksads_23_947_t | Suicidal ideation (Active, nonspecific) (Present) | Youth | 1-year follow-up |
|  | ksads_23_948_t | Suicidal ideation (Active, method) (Present) | Youth | 1-year follow-up |
|  | ksads_23_949_t | Suicidal ideation (Active, intent) (Present) | Youth | 1-year follow-up |
|  | ksads_23_950_t | Suicidal ideation (Active, plan) (Present) | Youth | 1-year follow-up |
|  | ksads_23_951_t | Preparatory actions toward imminent suicidal behavior (Present) | Youth | 1-year follow-up |
|  | ksads_23_956_t | Self-injurious behavior without suicidal intent (Past) | Youth | 1-year follow-up |
|  | ksads_23_957_t | Suicidal ideation (Passive) (Past) | Youth | 1-year follow-up |
|  | ksads_23_958_t | Suicidal ideation (Active, nonspecific) (Past) | Youth | 1-year follow-up |
|  | ksads_23_959_t | Suicidal ideation (Active, method) (Past) | Youth | 1-year follow-up |
|  | ksads_23_960_t | Suicidal ideation (Active, intent) (Past) | Youth | 1-year follow-up |
| ABCD Youth Diagnostic Interview for DSM-5 5 (KSADS-5) (abcd_ksad501) | ksads_23_961_t | Suicidal ideation (Active, plan) (Past) | Youth | 1-year follow-up |
|  | ksads_23_962_t | Preparatory actions toward imminent suicidal behavior (Past) | Youth | 1-year follow-up |
|  | ksads_23_964_t | Aborted attempt (Past) | Youth | 1-year follow-up |
|  | ksads_23_965_t | Suicide attempt (Past) | Youth | 1-year follow-up |
| ABCD Parent General Behavior Inventory-Mania (PGBI) (abcd_pgbi01) | gen_child_behav_1 | Has your child experienced periods of several days or more when, although he/she was feeling unusually happy and intensely energetic (clearly more than your child's usual self), he/she was also physically restless, unable to sit still, and had to keep moving or jumping from one activity to another? | Parent | 1-year follow-up |
|  | gen_child_behav_2 | Have there been periods of several days or more when your child's friends or other family members told you that your child seemed unusually happy or high - clearly different from his/her usual self or from a typical good mood? | Parent | 1-year follow-up |
|  | gen_child_behav_3 | Has your child's mood or energy shifted rapidly back and forth from happy to sad or high to low? | Parent | 1-year follow-up |
|  | gen_child_behav_4 | Has your child had periods of extreme happiness and intense energy that last several days or more when he/she also felt more anxious or tense (jittery, nervous, uptight) than usual (other than relates to the menstrual cycle)? | Parent | 1-year follow-up |
|  | gen_child_behav_5 | Have there been times of several days or more when, although your child was feeling unusually happy and intensely energetic (clearly more than his/her usual self), he/she also had to struggle very hard to control inner feelings of rage or an urge to smash or destroy things? | Parent | 1-year follow-up |
|  | gen_child_behav_6 | Has your child had periods of extreme happiness and intense energy (clearly more than his/her normal self) when, for several days or more, it took him/her over an hour to get to sleep at night? | Parent | 1-year follow-up |
|  | gen_child_behav_7 | Have you ever found that your child's feelings or energy are generally up or down, but rarely in the middle? | Parent | 1-year follow-up |
|  | gen_child_behav_8 | Has your child had periods lasting several days or more when he/she felt depressed or irritable, and then other periods of several days or more when he/she felt extremely high, elated, and overflowing with energy? | Parent | 1-year follow-up |
|  | gen_child_behav_9 | Have there been periods when, although your child was feeling unusually happy and intensely energetic, almost everything got on his/her nerves and made him/her irritable or angry (other than related to the menstrual cycle)? | Parent | 1-year follow-up |
| ABCD Parent General Behavior Inventory-Mania (PGBI) (abcd_pgbi01) | gen_child_behav_10 | Has your child had times when his/her thoughts and ideas came so fast that he/she couldn't get them all out, or they came so quickly others complained that they couldn't keep up with your child's ideas? | Parent | 1-year follow-up |

The full range of ABCD psychopathology variables used in factor analysis and generation. The ABCD instrument and item descriptions for each of the 93 measures that went into exploratory factor analyses are displayed in the leftmost three columns. Note that no summary variables were included. For every item, reporter and time point are displayed as well.

**Supplemental Table 5.** Parental mental health and drug use in ABCD Study

| Item | Item description | F1 | F2 | F3 |
| --- | --- | --- | --- | --- |
| famhx_ss_fath_prob_dg_p | Father drug use problem | **0.84** | 0.14 | -0.07 |
| famhx_ss_fath_prob_prf_p | Father been to a doctor or counselor due to emotional/mental problem | **0.81** | 0.06 | -0.03 |
| famhx_ss_fath_prob_nrv_p | Father nerves/nervous breakdown problem | **0.81** | 0.19 | -0.20 |
| famhx_ss_fath_prob_hspd_p | Father hospitalized due to emotional/mental problem | **0.78** | -0.08 | 0.21 |
| famhx_ss_fath_prob_scd_p | Father attempted or committed suicide | **0.77** | 0.04 | 0.01 |
| famhx_ss_fath_prob_ma_p | Father mania problem | **0.73** | -0.06 | 0.14 |
| famhx_ss_fath_prob_vs_p | Father visions of others spying/plotting problem | **0.70** | -0.04 | 0.14 |
| famhx_ss_moth_prob_dg_p | Mother drug use problem | 0.08 | **0.84** | -0.08 |
| famhx_ss_moth_prob_prf_p | Mother been to a doctor or counselor due to emotional/mental problem | 0.14 | **0.83** | -0.09 |
| famhx_ss_moth_prob_hspd_p | Mother hospitalized due to emotional/mental problem | -0.08 | **0.82** | 0.18 |
| famhx_ss_moth_prob_scd_p | Mother attempted or committed suicide | -0.05 | **0.77** | 0.07 |
| famhx_ss_moth_prob_ma_p | Mother mania problem | 0.02 | **0.73** | 0.15 |
| famhx_ss_moth_prob_nrv_p | Mother nerves/nervous breakdown problem | 0.23 | **0.71** | -0.12 |
| famhx_ss_moth_prob_vs_p | Mother visions of others spying/plotting problem | 0 | **0.62** | 0.14 |
| famhx_ss_fath_prob_dprs_p | Father depression problem | 0.30 | -0.14 | **0.79** |
| famhx_ss_moth_prob_dprs_p | Mother depression problem | -0.16 | 0.35 | **0.77** |
| famhx_ss_moth_prob_trb_p | Mother trouble with holding job/fighting/police | -0.23 | 0.41 | **0.71** |
| famhx_ss_fath_prob_trb_p | Father trouble with holding job/fighting/police | 0.34 | -0.05 | **0.67** |
| famhx_ss_moth_prob_alc_p | Mother alcohol problem | -0.12 | 0.30 | **0.64** |
| famhx_ss_fath_prob_alc_p | Father alcohol problem | 0.35 | -0.16 | **0.59** |
|  |  | Inter-Factor Correlations | | |
|  |  | F1 | F2 | F3 |
|  | F1 | 1 | 0.34 | 0.46 |
|  | F2 | 0.34 | 1 | 0.52 |
|  | F3 | 0.46 | 0.52 | 1 |
| Note. Largest absolute loading per row bolded; F = Factor; extraction method = least-squares; rotation = promax | | | | |

Results of the exploratory factor analysis of ABCD variables relating to parental mental health and drug use. *Factor 1* comprises variables related to paternal mental health, with the strongest indicator being paternal drug use issues. *Factor 2* comprises variables related to maternal mental health, with the strongest indicator being maternal drug use issues. *Factor 3* comprises variables most related to parental alcohol use issues, with the strongest indicators being paternal and maternal depression. Inter-factor correlations are shown at the bottom of the table.

**Supplemental Table 6.** Maternal drug use during pregnancy in ABCD Study

| Item | Item description | F1 | F2 | F3 |
| --- | --- | --- | --- | --- |
| devhx_9_oxycont | Oxycontin use knowing of pregnancy | **0.99** | 0.04 | -0.07 |
| devhx_9_her_morph | Heroin/Morphine use knowing of pregnancy | **0.96** | -0.03 | -0.02 |
| devhx_8_her_morph | Heroin/Morphine before knowing of pregnancy | **0.93** | 0.06 | 0.01 |
| devhx_9_other_drugs | Any other drug use knowing of pregnancy | **0.88** | -0.17 | 0.08 |
| devhx_8_oxycont | Oxycontin use before knowing of pregnancy | **0.85** | 0.12 | -0.03 |
| devhx_8_other_drugs | Any other drug use before knowing of pregnancy | **0.69** | 0.06 | -0.01 |
| devhx_9_coc_crack | Cocaine/Crack use knowing of pregnancy | **0.58** | 0.31 | 0.29 |
| devhx_8_coc_crack | Cocaine/Crack use before knowing of pregnancy | **0.56** | 0.28 | 0.31 |
| devhx_8_tobacco | Tobacco use before knowing of pregnancy | -0.08 | **1** | 0.01 |
| devhx_9_tobacco | Tobacco use knowing of pregnancy | 0.30 | **0.73** | -0.08 |
| devhx_8_marijuana | Marijuana use before knowing of pregnancy | 0.14 | **0.55** | 0.27 |
| devhx_9_marijuana | Marijuana use knowing of pregnancy | 0.38 | **0.44** | 0.18 |
| devhx_8_alcohol | Alcohol use before knowing of pregnancy | -0.13 | 0.07 | **0.92** |
| devhx_9_alcohol | Alcohol use knowing of pregnancy | 0.43 | -0.11 | **0.66** |
|  |  | Inter-Factor Correlations | | |
|  |  | F1 | F2 | F3 |
|  | F1 | 1 | 0.55 | 0.48 |
|  | F2 | 0.55 | 1 | 0.38 |
|  | F3 | 0.48 | 0.38 | 1 |
| Note. Largest absolute loading per row bolded; F = Factor; extraction method = least-squares; rotation = oblimin | | | | |

Results of the exploratory factor analysis of ABCD variables relating to maternal drug use during pregnancy. *Factor 1* comprises variables related to hard drug use during pregnancy, with the strongest indicator being oxycontin use (while knowing of pregnancy). *Factor 2* comprises variables related to tobacco or marijuana use during pregnancy, with the strongest indicator being tobacco use (before knowing of pregnancy). *Factor 3* comprises variables related to alcohol use during pregnancy, with the strongest indicator being alcohol use (before knowing of pregnancy). Inter-factor correlations are shown at the bottom of the table.

**Supplemental Table 7.** Geocoded data-derived neighborhood-level characteristics in ABCD Study

| Item | Item description | | | | | F1 | | F2 | F3 | F4 |
| --- | --- | --- | --- | --- | --- | --- | --- | --- | --- | --- |
| reshist_addr1_adi_in_dis | Income disparity (log of 100 x ratio of the number of households with <10000 annual income to the number of households with >50000 annual income) | | | | | **0.90** | | -0.07 | -0.10 | -0.06 |
| reshist_addr1_adi_ncar | Percentage of occupied housing units without a motor vehicle | | | | | **0.87** | | 0.08 | -0.03 | -0.16 |
| reshist_addr1_adi_home_o | Percentage of house ownership | | | | | **-0.82** | | -0.07 | -0.15 | -0.12 |
| reshist_addr1_leadrisk_poverty | Percentage of individuals below -125% of the poverty level | | | | | **0.79** | | -0.03 | -0.08 | 0.20 |
| reshist_addr1_adi_pov | Percentage of families below the poverty level | | | | | **0.64** | | -0.04 | -0.15 | 0.28 |
| reshist_addr1_adi_sp | Percentage of single owners/tenants | | | | | **0.55** | | 0.03 | -0.24 | 0.18 |
| reshist_addr1_adi_unemp | Unemployment rate (percentage of civilian labor force population above 16 and unemployed) | | | | | **0.53** | | 0.01 | -0.07 | 0.21 |
| reshist_addr1_adi_ntel | Percentage of occupied housing units without a telephone | | | | | **0.36** | | -0.04 | -0.09 | 0.06 |
| reshist_addr1_adi_nplumb | Percentage of occupied housing units without complete plumbing | | | | | **0.19** | | 0 | 0 | 0.06 |
| reshist_addr1_drgposs | Drug possession prevalence (derived from Unified Crime Reports) | | | | | -0.02 | | **1** | -0.02 | -0.01 |
| reshist_addr1_dui | DUI prevalence (derived from Unified Crime Reports) | | | | | -0.08 | | **0.99** | -0.02 | 0.01 |
| reshist_addr1_p1vlnt | Violent crime prevalence (derived from Unified Crime Reports) | | | | | 0.04 | | **0.98** | 0.04 | 0.02 |
| reshist_addr1_drgsale | Drug sale prevalence (derived from Unified Crime Reports) | | | | | 0.05 | | **0.97** | -0.01 | -0.01 |
| reshist_addr1_adi_home_v | Median home value | | | | | 0.07 | | 0.03 | **0.96** | 0.01 |
| reshist_addr1_adi_mortg | Median monthly mortgage | | | | | -0.06 | | 0.02 | **0.91** | 0.04 |
| reshist_addr1_adi_rent | Median gross rent | | | | | -0.20 | | -0.05 | **0.61** | 0.14 |
| reshist_addr1_adi_income | Median family income | | | | | -0.32 | | -0.02 | **0.58** | -0.30 |
| reshist_addr1_adi_crowd | Percentage of occupied housing units with more than 1 person per room (crowding) | | | | | 0.11 | | 0.04 | 0.11 | **0.78** |
| reshist_addr1_adi_edu_l | Percentage of population over 25 with less than 9 years of education | | | | | 0.22 | | 0.01 | -0.02 | **0.69** |
| reshist_addr1_adi_work_c | Percentage of employed persons above 16 in white collar occupations | | | | | 0.37 | | 0.05 | 0.27 | **-0.52** |
|  | |  |  |  |  | |  |  |  |  |

|  |  | Inter-Factor Correlations | | | |
| --- | --- | --- | --- | --- | --- |
|  |  | F1 | F2 | F3 | F4 |
|  | F1 | 1 | 0.11 | -0.46 | 0.48 |
|  | F2 | 0.11 | 1 | 0.24 | 0.02 |
|  | F3 | -0.46 | 0.24 | 1 | -0.25 |
|  | F4 | 0.48 | 0.02 | -0.25 | 1 |
| Note. Largest absolute loading per row bolded; F = Factor; extraction method = least-squares; rotation = oblimin | | | | | |

Results of the exploratory factor analysis of ABCD variables relating to neighborhood-level characteristics. *Factor 1* comprises variables related to neighborhood-level poverty, with the strongest indicator being income disparity. *Factor 2* comprises variables related to neighborhood-level crime, with the strongest indicator being prevalence of drug possession arrests. *Factor 3* comprises variables related to neighborhood-level affluence, with the strongest indicator being median home value. *Factor 4* comprises variables related to neighborhood-level population density, with the strongest indicator being home crowding (percentage of homes with more than 1 person per bedroom). Inter-factor correlations are shown at the bottom of the table.

**Supplemental Table 8.** Parent-reported household-/family-level poverty and religiosity in ABCD Study

| Item | Item description | F1 | F2 | F3 |
| --- | --- | --- | --- | --- |
| demo_fam_exp2_v2_l | Inability to afford telephone service | **0.91** | -0.02 | 0.03 |
| demo_fam_exp4_v2_l | Eviction due to inability to afford rent or mortgage | **0.87** | -0.06 | -0.04 |
| demo_fam_exp5_v2_l | Loss of utility services due to inability to pay gas, electric, or oil company | **0.84** | 0.01 | -0.03 |
| demo_fam_exp3_v2_l | Inability to pay full amount of rent or mortgage | **0.79** | 0.13 | 0.01 |
| demo_fam_exp1_v2_l | Inability to afford food or pay to go get it | **0.74** | 0.18 | 0.02 |
| demo_fam_exp7_v2_l | Inability to afford a needed visit to the dentist | 0.01 | **0.93** | 0.03 |
| demo_fam_exp6_v2_l | Inability to afford a needed visit to a doctor or to the hospital | 0.01 | **0.89** | -0.04 |
| demo_yrs_2_l | Parental importance of child's religious and spiritual beliefs | 0.06 | -0.01 | **0.89** |
| demo_yrs_1_l | Frequency of child’s attendance to religious services | -0.06 | 0.01 | **0.88** |
|  |  | Inter-Factor Correlations | | |
|  |  | F1 | F2 | F3 |
|  | F1 | 1 | 0.64 | 0.02 |
|  | F2 | 0.64 | 1 | 0.06 |
|  | F3 | 0.02 | 0.06 | 1 |
| Note. Largest absolute loading per row bolded; F = Factor; extraction method = principal-axis; rotation = geomin | | | | |

Results of the exploratory factor analysis of ABCD variables relating to household- and family-level poverty and religiosity. *Factor 1* comprises variables related to household poverty, with the strongest indicator being inability to afford telephone service. *Factor 2* comprises variables related to financial inability to seek medical treatment, with the strongest indicator being inability to afford a needed visit to the dentist. *Factor 3* comprises variables related to family religiosity, with the strongest indicator being parental importance of children’s religious/spiritual beliefs. Inter-factor correlations are shown at the bottom of the table.

**Supplemental Table 9.** Youth-reported school-level characteristics in ABCD Study

| Item | Item description | F1 | F2 | F3 | F4 |
| --- | --- | --- | --- | --- | --- |
| school_12_y | “In general, I like school a lot” | **0.88** | 0.03 | 0.03 | -0.01 |
| school_15_y | “Usually, school bores me” | **-0.78** | 0.03 | 0.03 | 0 |
| school_3_y | “I get along with my teachers” | **0.30** | 0.18 | 0.22 | 0.13 |
| school_10_y | “There are lots of chances to be part of class discussions or activities” | 0 | **0.84** | 0.01 | -0.02 |
| school_5_y | “There are lots of chances for students in my school to get involved in sports, clubs, or other school activities outside of class” | -0.02 | **0.50** | -0.04 | 0.04 |
| school_2_y | “In my school, students have lots of chances to help decide things like class activities and rules” | 0.04 | **0.46** | -0.01 | 0.12 |
| school_6_y | “I feel safe at my school” | 0.25 | **0.35** | 0.03 | 0.14 |
| school_8_y | “I like school because I do well in class” | 0.07 | -0.03 | **0.79** | 0.04 |
| school_17_y | “Getting good grades is not so important to me” | 0.12 | -0.02 | **-0.48** | 0.07 |
| school_9_y | “I feel I'm just as smart as other kids my age” | -0.02 | 0.21 | **0.47** | 0.02 |
| school_7_y | “The school lets my parents know when I have done something well” | 0 | -0.01 | -0.03 | **0.79** |
| school_4_y | “My teacher(s) notices when I am doing a good job and lets me know about it” | -0.03 | 0.10 | 0.20 | **0.49** |
|  |  | Inter-Factor Correlations | | | |
|  |  | F1 | F2 | F3 | F4 |
|  | F1 | 1 | 0.47 | 0.67 | 0.43 |
|  | F2 | 0.47 | 1 | 0.55 | 0.6 |
|  | F3 | 0.67 | 0.55 | 1 | 0.57 |
|  | F4 | 0.43 | 0.6 | 0.57 | 1 |
| Note. Largest absolute loading per row bolded; F = Factor; extraction method = least-squares; rotation = oblimin | | | | | |

Results of the exploratory factor analysis of ABCD variables relating to school-level characteristics. *Factor 1* comprises variables related to school enjoyment, with the strongest indicator being endorsement of “liking school a lot”. *Factor 2* comprises variables related to school involvement, with the strongest indicator being endorsement of “lots of chances to be part of class discussions or activities”. *Factor 3* comprises variables related to school achievement and grades, with the strongest indicator being endorsement of liking school “because I do well in class”. Factor 4 comprises variables related to positive reinforcement from school, with the strongest indicator being endorsement of the school informing parents “when I have done something well”. Inter-factor correlations are shown at the bottom of the table.

**Supplemental Table 10.** Pregnancy complications in ABCD Study

| Item | Item description | F1 | F2 | F3 |
| --- | --- | --- | --- | --- |
| devhx_10k3_p | Previa, abruptio, or other problems with the placenta during pregnancy | **0.85** | -0.04 | -0.14 |
| devhx_10f3_p | Rubella (German measles) during first 3 months of pregnancy | **0.58** | 0.14 | 0.53 |
| devhx_10b3_p | Heavy bleeding requiring bed rest or special treatment during pregnancy | **0.58** | 0.01 | 0.10 |
| devhx_10e3_p | Persistent proteinuria during pregnancy | **0.46** | 0.36 | 0.30 |
| devhx_10m3_p | Any other conditions requiring medical care during pregnancy | **0.45** | -0.04 | -0.01 |
| devhx_10l3_p | Accident or injury requiring medical care during pregnancy | **0.33** | 0.01 | 0.32 |
| devhx_10j3_p | Pregnancy-related high blood pressure | -0.06 | **0.93** | -0.01 |
| devhx_10c3_p | Pre-eclampsia, eclampsia, or toxemia during pregnancy | 0 | **0.91** | -0.05 |
| devhx_10i3_p | Pregnancy-related diabetes | 0.15 | **0.27** | 0.10 |
| devhx_10g3_p | Severe anemia during pregnancy | -0.05 | -0.03 | **0.71** |
| devhx_10h3_p | Urinary tract infections during pregnancy | -0.06 | -0.03 | **0.64** |
| devhx_10a3_p | Severe nausea and vomiting extending past the 6th month of pregnancy or accompanied by weight loss | -0.10 | 0.03 | **0.57** |
| devhx_10d3_p | Severe gall bladder attack during pregnancy | 0.26 | 0.13 | **0.41** |
|  |  | Inter-Factor Correlations | | |
|  |  | F1 | F2 | F3 |
|  | F1 | 1 | 0.42 | 0.54 |
|  | F2 | 0.42 | 1 | 0.45 |
|  | F3 | 0.54 | 0.45 | 1 |
| Note. Largest absolute loading per row bolded; F = Factor; extraction method = principal-axis; rotation = oblimin | | | | |

Results of the exploratory factor analysis of ABCD variables relating to pregnancy complications. *Factor 1* comprises variables most related to placental issues, with the strongest indicator being previa, abruptio, or other problems with the placenta during pregnancy. *Factor 2* comprises variables most related to blood pressure, with the strongest indicator being pregnancy-related high blood pressure. *Factor 3* comprises variables most related to blood oxygen, with the strongest indicator being severe anemia during pregnancy. Inter-factor correlations are shown at the bottom of the table.

**Supplemental Table 11.** Birth complications in ABCD Study

| Item | Item description | F1 | F2 | F3 |
| --- | --- | --- | --- | --- |
| devhx_14a3_p | Blue at birth | **0.93** | 0.06 | -0.05 |
| devhx_14c3_p | Did not breathe at first at birth | **0.77** | -0.05 | 0.19 |
| devhx_14b3_p | Slow heartbeat at birth | **0.65** | 0.09 | 0.05 |
| devhx_14d3_p | Convulsions at birth | 0.16 | **0.85** | -0.08 |
| devhx_14g3_p | Required blood transfusion at birth | -0.05 | **0.65** | 0.45 |
| devhx_14h3_p | Rh incompatibility at birth | -0.03 | **0.63** | -0.10 |
| devhx_14f3_p | Required oxygen at birth | 0.15 | -0.02 | **0.92** |
| devhx_14e3_p | Jaundice needing treatment at birth | -0.15 | 0.28 | **0.41** |
|  |  | Inter-Factor Correlations | | |
|  |  | F1 | F2 | F3 |
|  | F1 | 1 | 0.44 | 0.56 |
|  | F2 | 0.44 | 1 | 0.36 |
|  | F3 | 0.56 | 0.36 | 1 |
| Note. Largest absolute loading per row bolded; F = Factor; extraction method = least-squares; rotation = oblimin | | | | |

Results of the exploratory factor analysis of ABCD variables relating to birth complications. *Factor 1* comprises variables most related to circulation, with the strongest indicator being blue color at birth. *Factor 2* comprises variables most related to blood pressure, with the strongest indicator being convulsions at birth. *Factor 3* comprises variables most related to blood oxygen, with the strongest indicator being requiring oxygen at birth. Inter-factor correlations are shown at the bottom of the table.**Supplemental Table 12.** Parent-reported traumatic events experienced by child in ABCD Study

| Item | Item description | F1 | F2 | F3 | F4 |
| --- | --- | --- | --- | --- | --- |
| ksads_ptsd_raw_757_p | Witnessed or caught in a natural disaster that caused significant property damage or personal injury | **0.89** | -0.17 | -0.07 | -0.06 |
| ksads_ptsd_raw_762_p | Shot, stabbed, or beaten brutally by a grown up in the home | **0.89** | 0.22 | -0.04 | -0.01 |
| ksads_ptsd_raw_761_p | Shot, stabbed, or beaten brutally by a non-family member | **0.80** | 0.13 | 0.07 | 0.11 |
| ksads_ptsd_raw_759_p | Witnessed death or mass destruction in a war zone | **0.76** | -0.07 | 0.21 | 0.14 |
| ksads_ptsd_raw_758_p | Witnessed or present during an act of terrorism (e.g., Boston marathon bombing) | **0.75** | -0.06 | 0.31 | 0.02 |
| ksads_ptsd_raw_763_p | Beaten to the point of having bruises by a grown up in the home | **0.65** | 0.50 | -0.04 | -0.23 |
| ksads_ptsd_raw_756_p | Witnessed or caught in a fire that caused significant property damage or personal injury | **0.59** | -0.14 | 0.21 | 0 |
| ksads_ptsd_raw_760_p | Witnessed someone shot or stabbed in the community | **0.58** | 0.13 | 0.10 | 0.17 |
| ksads_ptsd_raw_754_p | Car accident in which your child or another person in the car was hurt bad enough to require medical attention | **0.55** | 0.03 | -0.05 | 0.12 |
| ksads_ptsd_raw_755_p | A significant accident for which the child needed specialized and intensive medical treatment | **0.55** | -0.06 | 0.09 | -0.06 |
| ksads_ptsd_raw_765_p | A family member threatened to kill the child | **0.48** | 0.13 | 0.43 | -0.11 |
| ksads_ptsd_raw_764_p | A non-family member threatened to kill the child | **0.45** | 0.02 | 0.44 | 0.03 |
| ksads_ptsd_raw_766_p | Witness the grownups in the home push, shove or hit one another | -0.25 | **1.10** | 0.01 | 0.09 |
| ksads_ptsd_raw_769_p | A peer forced the child to do something sexually | 0.18 | -0.05 | **0.69** | 0 |
| ksads_ptsd_raw_768_p | An adult outside the family touched the child in his or her privates, had the child touch their privates or did other sexual things to the child | 0.28 | 0.07 | **0.6** | 0.02 |
| ksads_ptsd_raw_767_p | A grown up in the home touched the child in his or her privates, had the child touch their privates, or did other sexual things to the child | 0.37 | 0.23 | **0.47** | -0.04 |
| ksads_ptsd_raw_770_p | Learned about the sudden unexpected death of a loved one | 0.01 | 0.04 | -0.01 | **0.78** |

|  |  | Inter-Factor Correlations | | | |
| --- | --- | --- | --- | --- | --- |
|  |  | F1 | F2 | F3 | F4 |
|  | F1 | 1 | 0.62 | 0.82 | 0.43 |
|  | F2 | 0.62 | 1 | 0.63 | 0.31 |
|  | F3 | 0.82 | 0.63 | 1 | 0.35 |
|  | F4 | 0.43 | 0.31 | 0.35 | 1 |
| Note. Scree plot suggested one factor, but due to the importance of trauma in the exposome, the solution was intentionally over-extracted to four factors (the maximum number before additional factors could no longer be identified by any rotation); the single-indicator factors (2 and 4) and Heywood case (factor 2) were used in the overall factor analysis as individual dichotomous items, and factors 1 and 3 have unit-weighting in composite (i.e. they are sum scores); largest absolute loading per row bolded; F = Factor; extraction method = least-squares; rotation = promax | | | | | |

Results of the exploratory factor analysis of ABCD variables relating to traumatic events. *Factor 1* comprises variables related to miscellaneous threatening childhood traumas, with the strongest indicator being witnessing or being caught in a significant natural disaster. *Factor 2* comprises only one variable related to family conflict, that being witnessing grownups in the home shove, push, or hit each other. *Factor 3* comprises variables related to childhood sexual assault, with the strongest indicator being a peer forcing the child to do something sexually. Factor 4 comprised only one variable related to death in the family, that being sudden unexpected death of a loved one. Inter-factor correlations are shown at the bottom of the table.

**Supplemental Table 13.** Youth-reported life events in ABCD Study

| Item | Item description | F1 | F2 | F3 | F4 | F5 |
| --- | --- | --- | --- | --- | --- | --- |
| ple_jail_y | One of the parents/caregivers went to jail | **1** | -0.06 | -0.05 | 0.03 | 0 |
| ple_arrest_y | Someone in the family was arrested | **0.87** | 0.02 | 0.08 | -0.04 | -0.02 |
| ple_law_y | Parents/caregivers got into trouble with the law | **0.71** | 0.12 | -0.01 | 0.05 | 0.06 |
| ple_sud_y | Family member had drug and/or alcohol problem | **0.33** | 0.30 | 0.12 | 0.02 | -0.04 |
| ple_financial_y | Negative change in parent's financial situation | -0.03 | **0.63** | 0.05 | 0.09 | -0.01 |
| ple_argue_y | Parents argued more than previously | 0.01 | **0.57** | 0.04 | 0.14 | -0.05 |
| ple_job_y | Mother/father figure lost job | 0.12 | **0.47** | -0.08 | 0.01 | 0.11 |
| ple_away_y | One parent was away from home more often | 0.10 | **0.40** | 0 | -0.04 | 0.10 |
| ple_mh_y | Family member had mental/emotional problem | 0.10 | **0.34** | 0.21 | 0 | 0.03 |
| ple_victim_y | Child was a victim of crime/violence/assault | 0.12 | **0.25** | 0.24 | 0.02 | -0.05 |
| ple_sib_y | Brother or sister left home | 0.14 | **0.21** | 0.10 | 0.04 | -0.07 |
| ple_friend_died_y | Child’s close friend died | -0.01 | -0.08 | **0.73** | 0.07 | -0.08 |
| ple_friend_y | Child lost a close friend | 0 | 0.06 | **0.54** | 0.04 | 0.10 |
| ple_friend_injur_y | Child’s close friend was seriously sick/injured | 0.02 | 0.11 | **0.51** | -0.06 | 0.07 |
| ple_ill_y | Child was seriously sick | 0.10 | 0.17 | **0.32** | -0.07 | 0.10 |
| ple_injured_y | Family member was seriously injured | 0.12 | 0.18 | **0.29** | -0.12 | 0.11 |
| ple_injur_y | Child was seriously injured | 0.05 | 0.20 | **0.28** | -0.12 | 0.18 |
| ple_crime_y | Child saw crime or accident | 0.12 | 0.18 | **0.26** | -0.08 | 0.07 |
| ple_died_y | Someone in family died | 0.11 | 0.05 | **0.11** | 0.02 | 0.10 |
| ple_step_y | Child got new stepmother or stepfather | 0 | -0.08 | 0.07 | **0.86** | 0.09 |
| ple_separ_y | Parents separated or divorced | 0.06 | 0.14 | -0.05 | **0.83** | -0.05 |
| ple_move_y | Family moved | -0.01 | -0.03 | 0 | 0.06 | **0.76** |
| ple_school_y | Child attended a new school | 0.03 | 0.02 | 0 | -0.01 | **0.59** |
| ple_new_job_y | Parent/caregiver got a new job | 0 | 0.29 | -0.02 | -0.01 | **0.40** |
| ple_new_sib_y | Child got new brother or sister | 0.06 | -0.11 | 0.05 | 0.10 | **0.39** |
|  |  | Inter-Factor Correlations | | | | |
|  |  | F1 | F2 | F3 | F4 | F5 |
|  | F1 | 1 | 0.45 | 0.28 | 0.47 | 0.27 |
|  | F2 | 0.45 | 1 | 0.33 | 0.28 | 0.22 |
|  | F3 | 0.28 | 0.33 | 1 | 0.15 | 0.19 |
|  | F4 | 0.47 | 0.28 | 0.15 | 1 | 0.34 |
|  | F5 | 0.27 | 0.22 | 0.19 | 0.34 | 1 |
| Note. Largest absolute loading per row bolded; F = Factor; extraction method = least-squares; rotation = oblimin | | | | | | |

Results of the exploratory factor analysis of ABCD variables relating to major life events. *Factor 1* comprises variables related to legal trouble, with the strongest indicator being jailtime for a parent or caregiver. *Factor 2* comprises variables related to family discordance, with the strongest indicator being negative change in parental financial situation. *Factor 3* comprises variables related to witnessing or experiencing casualty, with the strongest indicator being the death of a close friend. *Factor 4* comprises variables related to parental separation, with the strongest indicator being the addition of a new stepparent. *Factor 5* comprises variables related to major family life changes, with the strongest indicator being a family move. Inter-factor correlations are shown at the bottom of the table.

**Supplemental Table 14.** Bifactor indices^1^ for the exposome models (ABCD Study and PNC)

|  | ABCD Model | | | | | | | PNC Model | | | | | | |
| --- | --- | --- | --- | --- | --- | --- | --- | --- | --- | --- | --- | --- | --- | --- |
| **Factor** | **Explained Common Variance (ECV) (SS)** | **Explained Common Variance (ECV) (SG)** | **Explained Common Variance (ECV) (GS)** | **Omega** | **Omega-hierarchical (OmegaH)** | **H** | **Factor Determinacy (FD)** | **Explained Common Variance (ECV) (SS)** | **Explained Common Variance (ECV) (SG)** | **Explained Common Variance (ECV) (GS)** | **Omega** | **Omega-hierarchical (OmegaH)** | **H** | **Factor Determinacy (FD)** |
| **General** | 0.211 | 0.211 | 0.211 | 0.790 | 0.248 | 0.863 | 0.905 | 0.451 | 0.451 | 0.451 | 0.650 | 0.030 | 0.932 | 0.966 |
| **F1** | 0.475 | 0.120 | 0.445 | 0.745 | 0.601 | 0.792 | 0.877 | 0.904 | 0.158 | 0.096 | 0.729 | 0.677 | 0.739 | 0.864 |
| **F2** | 0.401 | 0.122 | 0.242 | 0.544 | 0.184 | 0.823 | 0.908 | 0.249 | 0.130 | 0.751 | 0.212 | 0.011 | 0.701 | 0.860 |
| **F3** | 0.631 | 0.124 | 0.343 | 0.403 | 0.160 | 0.801 | 0.888 | 0.893 | 0.246 | 0.107 | 0.825 | 0.751 | 0.829 | 0.916 |
| **F4** | 0.759 | 0.149 | 0.129 | 0.838 | 0.789 | 0.903 | 0.954 | 0.544 | 0.014 | 0.456 | 0.185 | 0.154 | 0.149 | 0.399 |
| **F5** | 0.838 | 0.150 | 0.050 | 0.843 | 0.755 | 0.959 | 0.980 |  |  |  |  |  |  |  |
| **F6** | 0.972 | 0.124 | 0.028 | 0.801 | 0.799 | 0.866 | 0.932 |  |  |  |  |  |  |  |

Abbreviations: ECV = explained common variance; ECV_SS = ECV of a specific factor with respect to itself; ECV_SG = ECV of a specific factor with respect to the general factor; ECV_GS = ECV of the general factor with respect to a specific factor; F = factor.

**Supplemental Table 15.** Exploratory factor analysis of optimized collection of ABCD psychopathology variables using iterated target rotation

| Item | Item description | F1 | F2 | F3 | F4 | F5 | F6 |
| --- | --- | --- | --- | --- | --- | --- | --- |
| prodromal_20_y | Have you seen things that other people can't see or don't seem to see? | **0.84** | 0.01 | 0.04 | 0 | -0.06 | -0.01 |
| prodromal_19_y | Did you suddenly start to see unusual things that you never saw before like flashes, flames, blinding light, or shapes floating in front of you? | **0.80** | 0.03 | 0.05 | -0.03 | -0.04 | -0.02 |
| prodromal_16_y | Did you feel that parts of your body had suddenly changed or worked differently than before; like your legs had suddenly turned to something else or your nose could suddenly smell things you'd never actually smelled before? | **0.76** | -0.02 | 0 | -0.01 | -0.02 | 0.10 |
| prodromal_4_y | Did you feel like you had special, unusual powers like you could make things happen by magic, or that you could magically know what was inside another person's mind, or magically know what was going to happen in the future when other people could not? | **0.76** | -0.06 | 0.09 | -0.01 | -0.05 | 0.08 |
| prodromal_14_y | Did you feel confused because something you experienced didn't seem real or it seemed imaginary to you? | **0.75** | 0 | -0.03 | 0 | -0.02 | -0.03 |
| prodromal_11_y | Although you could not see anything or anyone, did you suddenly start to feel that an invisible energy, creature, or some person was around you? | **0.75** | 0.05 | 0 | -0.03 | 0.02 | -0.03 |
| prodromal_10_y | Did you lose concentration because you noticed sounds in the distance that you usually don't hear? | **0.74** | 0.03 | -0.02 | 0.02 | 0.03 | 0.02 |
| prodromal_7_y | Did you ever feel very certain that you have very special abilities or magical talents that other people do not have? | **0.73** | 0.03 | 0.11 | -0.01 | -0.09 | 0.08 |
| prodromal_17_y | Did you feel that sometimes your thoughts were so strong you could almost hear them, as if another person, NOT you, spoke them? | **0.71** | 0.03 | -0.02 | -0.01 | 0.03 | 0.01 |
| prodromal_12_y | Did you start to worry at times that your mind was trying to trick you or was not working right? | **0.70** | 0.06 | -0.04 | 0.05 | -0.02 | -0.01 |
| prodromal_3_y | Do things that you see appear different from the way they usually do (brighter or duller, larger or smaller, or changed in some other way)? | **0.70** | 0.02 | -0.04 | 0.03 | 0 | -0.01 |
| prodromal_21_y | Did you suddenly start to notice that people sometimes had a hard time understanding what you were saying, even though they used to understand you well? | **0.70** | -0.12 | -0.03 | 0.06 | 0.12 | -0.01 |
| prodromal_2_y | Did you hear strange sounds that you never noticed before like banging, clicking, hissing, clapping, or ringing in your ears? | **0.68** | 0.06 | 0 | -0.04 | 0.01 | -0.04 |
| prodromal_1_y | Did places that you know well, such as your bedroom, or other rooms in your home, your classroom or school yard, suddenly seem weird, strange or confusing to you; like not the real world? | **0.68** | 0.04 | 0.01 | 0 | 0.03 | -0.02 |
| prodromal_8_y | Did you suddenly feel that you could not trust other people because they seemed to be watching you or talking about you in an unfriendly way? | **0.67** | -0.06 | -0.07 | 0.08 | 0.10 | -0.06 |
| prodromal_9_y | Do you sometimes get strange feelings on or just beneath your skin, like bugs crawling? | **0.66** | -0.03 | 0.01 | -0.01 | 0.08 | 0 |
| prodromal_6_y | Did you suddenly find it hard to figure out how to say something quickly and easily so that other people would understand what you meant? | **0.66** | -0.08 | -0.06 | 0.07 | 0.10 | -0.01 |
| prodromal_5_y | Did you feel that someone else, who is not you, has taken control over the private, personal, thoughts or ideas inside your head? | **0.62** | 0.18 | 0.02 | 0 | 0.06 | 0.02 |
| prodromal_13_y | Did you feel that the world is not real, you are not real, or that you are dead? | **0.61** | 0.18 | -0.02 | -0.03 | 0.02 | -0.05 |
| prodromal_18_y | Did you feel that other people might want something bad to happen to you or that you could not trust other people? | **0.59** | 0.09 | -0.03 | 0.01 | 0.13 | -0.06 |
| prodromal_15_y | Did you honestly believe in things that other people would say are unusual or weird? | **0.56** | 0.08 | 0.04 | -0.06 | 0.01 | -0.01 |
| ksads_23_951_t | Preparatory actions toward imminent suicidal behavior (Present) | -0.05 | **0.90** | -0.05 | 0.14 | 0 | 0.05 |
| ksads_23_961_t | Suicidal ideation (Active, plan) (Past) | -0.01 | **0.89** | 0.15 | -0.11 | -0.03 | 0.13 |
| ksads_23_948_t | Suicidal ideation (Active, method) (Present) | 0.02 | **0.87** | -0.15 | 0.09 | 0.10 | -0.07 |
| ksads_23_950_t | Suicidal ideation (Active, plan) (Present) | 0.02 | **0.86** | -0.13 | 0.13 | 0.03 | -0.01 |
| ksads_23_958_t | Suicidal ideation (Active, nonspecific) (Past) | 0.03 | **0.86** | 0.13 | -0.07 | 0.06 | -0.02 |
| ksads_23_962_t | Preparatory actions toward imminent suicidal behavior (Past) | 0.08 | **0.85** | 0.18 | -0.08 | -0.13 | 0.05 |
| ksads_23_960_t | Suicidal ideation (Active, intent) (Past) | 0.05 | **0.82** | 0.22 | -0.08 | -0.04 | -0.01 |
| ksads_23_947_t | Suicidal ideation (Active, nonspecific) (Present) | 0.05 | **0.81** | -0.15 | 0.08 | 0.07 | -0.12 |
| ksads_23_949_t | Suicidal ideation (Active, intent) (Present) | 0.08 | **0.81** | -0.21 | 0.18 | 0.02 | -0.05 |
| ksads_23_957_t | Suicidal ideation (Passive) (Past) | 0.03 | **0.78** | 0.07 | -0.05 | 0.06 | -0.06 |
| ksads_23_959_t | Suicidal ideation (Active, method) (Past) | 0.02 | **0.76** | 0.12 | -0.08 | 0.10 | 0.03 |
| ksads_23_946_t | Suicidal ideation (Passive) (Present) | 0.13 | **0.64** | -0.11 | 0.15 | 0.10 | -0.10 |
| ksads_23_964_t | Aborted suicide attempt (Past) | 0.2 | **0.64** | 0.14 | -0.15 | 0.03 | 0.07 |
| ksads_23_956_t | Self-injurious behavior without suicidal intent (Past) | 0.04 | **0.57** | 0 | -0.03 | 0.13 | -0.10 |
| ksads_23_945_t | Self-injurious behavior without suicidal intent (Present) | 0.11 | **0.54** | -0.06 | 0.01 | 0.13 | -0.08 |
| ksads_23_965_t | Suicide attempt (Past) | 0.13 | **0.51** | 0.09 | 0.09 | 0.10 | -0.10 |
| ksads_14_90_p | Impulsivity for more than one school year (Past) | -0.06 | 0.10 | **0.8** | 0.06 | 0.10 | -0.01 |
| ksads_14_88_p | Impulsivity (Present) | 0.07 | 0.02 | **0.76** | 0.07 | 0.05 | 0.01 |
| ksads_14_85_p | Difficulty remaining seated since elementary school (Present) | 0.07 | -0.07 | **0.74** | 0.06 | 0.09 | -0.01 |
| ksads_15_95_p | Often disobeys rules/requests (Present) | -0.01 | 0.05 | **0.73** | 0.10 | -0.01 | -0.03 |
| ksads_14_87_p | Difficulty remaining seated for more than one school year (Past) | -0.03 | -0.03 | **0.72** | 0.08 | 0.14 | -0.01 |
| ksads_15_93_p | Often argues with adults/authority (Present) | -0.03 | 0.10 | **0.71** | 0.06 | -0.01 | -0.03 |
| ksads_14_79_p | Difficulty sustaining attention for more than one school year (Past) | 0.02 | -0.02 | **0.69** | 0.04 | 0.14 | -0.03 |
| ksads_14_83_p* | Easily distracted for more than one school year (Past) |  |  |  |  |  |  |
| ksads_14_77_p* | Difficulty sustaining attention since elementary school (Present) | 0.06 | -0.07 | **0.66** | 0.09 | 0.09 | -0.05 |
| ksads_14_81_p** | Easily distracted since elementary school (Present) |  |  |  |  |  |  |
| ksads_15_96_p** | Often disobeys rules/requests (Past) | -0.04 | 0.10 | **0.64** | 0.16 | -0.02 | -0.01 |
| ksads_15_94_p | Often argues with adults/authority (Past) | -0.07 | 0.09 | **0.62** | 0.14 | 0.02 | -0.03 |
| ksads_16_98_p | Often lies (Present) | 0.09 | 0.02 | **0.60** | 0.17 | -0.05 | 0.04 |
| ksads_16_107_p | Stealing (Past) | 0.1 | -0.03 | **0.56** | 0.07 | -0.06 | -0.03 |
| ksads_16_105_p | Often bullies others (Past) | 0.05 | -0.02 | **0.53** | 0.16 | -0.05 | -0.04 |
| ksads_16_106_p | Stealing (Present) | 0.14 | 0 | **0.50** | 0.07 | -0.07 | -0.03 |
| ksads_16_99_p | Often lies (Past) | 0.13 | 0.02 | **0.50** | 0.17 | -0.07 | 0.01 |
| ksads_16_104_p | Often bullies others (Present) | 0.09 | -0.07 | **0.47** | 0.17 | -0.07 | -0.07 |
| gen_child_behav_4 | Has your child had periods of extreme happiness and intense energy that last several days or more when he/she also felt more anxious or tense (jittery, nervous, uptight) than usual (other than relates to the menstrual cycle)? | 0.06 | -0.14 | 0.02 | **0.87** | 0.03 | 0 |
| gen_child_behav_8 | Has your child had periods lasting several days or more when he/she felt depressed or irritable, and then other periods of several days or more when he/she felt extremely high, elated, and overflowing with energy? | -0.02 | 0.06 | 0.01 | **0.85** | 0 | -0.02 |
| gen_child_behav_9 | Have there been periods when, although your child was feeling unusually happy and intensely energetic, almost everything got on his/her nerves and made him/her irritable or angry (other than related to the menstrual cycle)? | -0.06 | 0.08 | 0.09 | **0.80** | -0.02 | 0 |
| gen_child_behav_5 | Have there been times of several days or more when, although your child was feeling unusually happy and intensely energetic (clearly more than his/her usual self), he/she also had to struggle very hard to control inner feelings of rage or an urge to smash or destroy things? | 0.04 | 0.06 | 0.09 | **0.80** | -0.02 | 0.01 |
| gen_child_behav_2 | Have there been periods of several days or more when your child's friends or other family members told you that your child seemed unusually happy or high - clearly different from his/her usual self or from a typical good mood? | 0.06 | -0.03 | -0.01 | **0.80** | -0.01 | 0.02 |
| gen_child_behav_6 | Has your child had periods of extreme happiness and intense energy (clearly more than his/her normal self) when, for several days or more, it took him/her over an hour to get to sleep at night? | 0.04 | -0.05 | 0.01 | **0.78** | 0.03 | 0.03 |
| gen_child_behav_3 | Has your child's mood or energy shifted rapidly back and forth from happy to sad or high to low? | -0.04 | 0.09 | 0.05 | **0.77** | -0.03 | -0.02 |
| gen_child_behavb_7 | Have you ever found that your child's feelings or energy are generally up or down, but rarely in the middle? | -0.02 | 0.04 | 0.13 | **0.75** | -0.03 | -0.01 |
| gen_child_behav_1 | Has your child experienced periods of several days or more when, although he/she was feeling unusually happy and intensely energetic (clearly more than your child's usual self), he/she was also physically restless, unable to sit still, and had to keep moving or jumping from one activity to another? | 0 | -0.02 | 0.15 | **0.72** | 0.04 | 0.03 |
| gen_child_behav_10 | Has your child had times when his/her thoughts and ideas came so fast that he/she couldn't get them all out, or they came so quickly others complained that they couldn't keep up with your child's ideas? | -0.04 | 0.03 | 0.18 | **0.60** | 0.07 | 0.03 |
| bpm_14_y | "I am inattentive or easily distracted" | 0.14 | -0.11 | 0.17 | -0.04 | **0.66** | -0.04 |
| bpm_10_y | "I act without stopping to think" | 0.01 | 0.06 | 0.14 | -0.06 | **0.63** | 0 |
| bpm_4_y | "I have trouble concentrating or paying attention" | 0.12 | -0.04 | 0.17 | -0.01 | **0.59** | -0.12 |
| bpm_16_y | "I have a hot temper" | 0.04 | 0.04 | 0.08 | 0.10 | **0.57** | 0.02 |
| bpm_7_y | "I disobey my parents" | -0.11 | 0.12 | 0.15 | 0.01 | **0.56** | 0 |
| bpm_2_y | "I argue a lot" | 0.02 | 0.06 | 0.12 | -0.01 | **0.56** | -0.03 |
| bpm_3_y | "I fail to finish things I start" | 0.05 | -0.02 | 0.05 | -0.01 | **0.55** | -0.07 |
| bpm_11_y | "I am too fearful or anxious" | 0.11 | 0.08 | -0.19 | 0.11 | **0.55** | 0.03 |
| bpm_15_y | "I am stubborn" | -0.1 | 0.12 | 0 | 0 | **0.55** | 0.05 |
| bpm_12_y | "I feel too guilty" | 0.14 | 0.10 | -0.09 | 0.04 | **0.52** | 0.02 |
| bpm_1_y | "I act too young for my age" | 0.09 | -0.03 | -0.01 | 0.01 | **0.52** | 0.03 |
| bpm_5_y | "I have trouble sitting still" | 0.13 | -0.05 | 0.17 | -0.02 | **0.52** | -0.02 |
| bpm_13_y | "I am self-conscious or easily embarrassed" | 0.09 | 0.11 | -0.20 | 0.02 | **0.50** | -0.01 |
| bpm_17_y | "I threaten to hurt people" | 0.02 | 0.23 | 0.12 | 0.04 | **0.49** | 0.03 |
| bpm_8_y | "I disobey at school" | 0 | 0.13 | 0.19 | 0.01 | **0.48** | -0.02 |
| bpm_9_y | "I feel worthless or inferior" | 0.16 | 0.27 | -0.16 | 0.07 | **0.48** | -0.07 |
| bpm_19_y | "I worry a lot" | 0.17 | 0.13 | -0.21 | 0.14 | **0.47** | 0.02 |
| bpm_6_y | "I destroy things belonging to others" | 0.07 | 0.11 | 0.14 | 0.07 | **0.45** | 0.01 |
| bpm_18_y | "I am unhappy, sad, or depressed" | 0.13 | 0.33 | -0.08 | 0.12 | **0.38** | -0.08 |
| poa_nihtb_5_y | "I felt enthusiastic" | 0.03 | -0.05 | 0.01 | 0.02 | 0.14 | **0.77** |
| poa_nihtb_7_y | "I felt confident" | 0.05 | -0.05 | 0.03 | -0.01 | -0.07 | **0.76** |
| poa_nihtb_6_y | "I felt interested" | 0.02 | 0.04 | -0.04 | -0.01 | 0.08 | **0.75** |
| poa_nihtb_2_y | "I felt delighted" | -0.03 | 0.04 | -0.04 | 0.01 | 0.06 | **0.74** |
| poa_nihtb_8_y | "I felt energetic" | 0.03 | -0.06 | 0.06 | -0.01 | 0.14 | **0.71** |
| poa_nihtb_4_y | "I felt at ease" | -0.02 | 0 | -0.02 | 0.01 | -0.05 | **0.70** |
| poa_nihtb_3_y | "I felt calm" | 0 | -0.05 | -0.07 | 0.06 | -0.13 | **0.60** |
| poa_nihtb_9_y | "I felt able to concentrate" | -0.05 | 0.07 | -0.10 | 0.06 | -0.31 | **0.59** |
| poa_nihtb_1_y | "I felt attentive” | -0.05 | 0.14 | -0.12 | 0.02 | -0.10 | **0.52** |
|  |  | Inter-Factor Correlations | | | | | |
|  |  | F1 | F2 | F3 | F4 | F5 | F6 |
|  | F1 | 1 | 0.48 | 0.15 | 0.22 | 0.51 | -0.06 |
|  | F2 | 0.48 | 1 | 0.13 | 0.24 | 0.39 | -0.13 |
|  | F3 | 0.15 | 0.13 | 1 | 0.46 | 0.22 | -0.05 |
|  | F4 | 0.22 | 0.24 | 0.46 | 1 | 0.22 | -0.07 |
|  | F5 | 0.51 | 0.39 | 0.22 | 0.22 | 1 | -0.25 |
|  | F6 | -0.06 | -0.13 | -0.05 | -0.07 | -0.25 | 1 |
| Note. F = Factor. | | | | | | | |
| * Items combined for purposes of factor analysis | | | | | | | |
| ** Items combined for purposes of factor analysis | | | | | | | |

Results of exploratory factor analysis of psychopathology items in the ABCD Study, using iterated target rotation designed to detect complex structure (cross-loadings). *Factor 1* comprises variables most related to symptoms of psychosis and associated prodrome, with the strongest indicators being seeing things that other people cannot or do not, seeing new and unusual things (i.e., flashes, flames, and shapes floating in front of the participant), and feeling like parts of the body were changing or working differently than before (i.e., legs turning to something else, nose able to smell new smells). *Factor 2* comprises variables most related to suicidal ideation or attempt (suicidality), with the strongest indicators being current preparatory actions for a suicide attempt and past or present active suicidal ideation (having a plan or method). *Factor 3* comprises variables most related to externalizing symptoms, with the strongest indicators being past or present impulsivity and difficulty remaining seated in school. *Factor 4* comprises variables most related to manic symptoms, with the strongest indicators being periods of extreme happiness/intense energy accompanied by heightened anxiety/tenseness, several-day periods of both depression/irritability and extreme happiness/intense energy, and periods of high irritability accompanied by unusual happiness/high energy. *Factor 5* comprises variables most related to self-reported symptoms, with the strongest indicators being reported inattentiveness/distractibility, acting without stopping to think, and trouble concentrating or paying attention. *Factor 6* comprises variables most related to self-reported positive affect, with the strongest indicators being reported enthusiasm, confidence, and interestedness. Inter-factor correlations are shown at the bottom of the table.

**Supplemental Table 16.** Final confirmatory bifactor analysis of optimized collection of ABCD psychopathology variables

| Item | Item description | General | F1 | F2 | F3 | F4 | F5 | F6 |
| --- | --- | --- | --- | --- | --- | --- | --- | --- |
| prodromal_1_y | Did places that you know well, such as your bedroom, or other rooms in your home, your classroom or school yard, suddenly seem weird, strange or confusing to you; like not the real world? | 0.476 | **0.539** |  |  |  |  |  |
| prodromal_2_y | Did you hear strange sounds that you never noticed before like banging, clicking, hissing, clapping, or ringing in your ears? | 0.448 | **0.562** |  |  |  |  |  |
| prodromal_3_y | Do things that you see appear different from the way they usually do (brighter or duller, larger or smaller, or changed in some other way)? | 0.429 | **0.563** |  |  |  |  |  |
| prodromal_4_y | Did you feel like you had special, unusual powers like you could make things happen by magic, or that you could magically know what was inside another person's mind, or magically know what was going to happen in the future when other people could not? | 0.389 | **0.639** |  |  |  |  |  |
| prodromal_5_y | Did you feel that someone else, who is not you, has taken control over the private, personal, thoughts or ideas inside your head? | 0.544 | **0.502** |  |  |  |  |  |
| prodromal_6_y | Did you suddenly find it hard to figure out how to say something quickly and easily so that other people would understand what you meant? | 0.448 | **0.514** |  |  |  |  |  |
| prodromal_7_y | Did you ever feel very certain that you have very special abilities or magical talents that other people do not have? | 0.391 | **0.639** |  |  |  |  |  |
| prodromal_8_y | Did you suddenly feel that you could not trust other people because they seemed to be watching you or talking about you in an unfriendly way? | 0.490 | **0.521** |  |  |  |  |  |
| prodromal_9_y | Do you sometimes get strange feelings on or just beneath your skin, like bugs crawling? | 0.452 | **0.518** |  |  |  |  |  |
| prodromal_10_y | Did you lose concentration because you noticed sounds in the distance that you usually don't hear? | 0.480 | **0.604** |  |  |  |  |  |
| prodromal_11_y | Although you could not see anything or anyone, did you suddenly start to feel that an invisible energy, creature, or some person was around you? | 0.499 | **0.599** |  |  |  |  |  |
| prodromal_12_y | Did you start to worry at times that your mind was trying to trick you or was not working right? | 0.454 | **0.557** |  |  |  |  |  |
| prodromal_13_y | Did you feel that the world is not real, you are not real, or that you are dead? | 0.496 | **0.502** |  |  |  |  |  |
| prodromal_14_y | Did you feel confused because something you experienced didn't seem real or it seemed imaginary to you? | 0.428 | **0.594** |  |  |  |  |  |
| prodromal_15_y | Did you honestly believe in things that other people would say are unusual or weird? | 0.381 | **0.457** |  |  |  |  |  |
| prodromal_16_y | Did you feel that parts of your body had suddenly changed or worked differently than before; like your legs had suddenly turned to something else or your nose could suddenly smell things you'd never actually smelled before? | 0.392 | **0.624** |  |  |  |  |  |
| prodromal_17_y | Did you feel that sometimes your thoughts were so strong you could almost hear them, as if another person, NOT you, spoke them? | 0.458 | **0.576** |  |  |  |  |  |
| prodromal_18_y | Did you feel that other people might want something bad to happen to you or that you could not trust other people? | 0.547 | **0.491** |  |  |  |  |  |
| prodromal_19_y | Did you suddenly start to see unusual things that you never saw before like flashes, flames, blinding light, or shapes floating in front of you? | 0.481 | **0.642** |  |  |  |  |  |
| prodromal_20_y | Have you seen things that other people can't see or don't seem to see? | 0.483 | **0.669** |  |  |  |  |  |
| prodromal_21_y | Did you suddenly start to notice that people sometimes had a hard time understanding what you were saying, even though they used to understand you well? | 0.481 | **0.530** |  |  |  |  |  |
| ksads_23_945_t | Self injurious behavior without suicidal intent (Present) | 0.532 |  | **0.384** |  |  |  |  |
| ksads_23_946_t | Suicidal ideation (Passive) (Present) | 0.654 |  | **0.501** |  |  |  |  |
| ksads_23_947_t | Suicidal ideation (Active, nonspecific) (Present) | 0.602 |  | **0.792** |  |  |  |  |
| ksads_23_948_t | Suicidal ideation (Active, method) (Present) | 0.624 |  | **0.751** |  |  |  |  |
| ksads_23_949_t | Suicidal ideation (Active, intent) (Present) | 0.586 |  | **0.765** |  |  |  |  |
| ksads_23_950_t | Suicidal ideation (Active, plan) (Present) | 0.572 |  | **0.745** |  |  |  |  |
| ksads_23_951_t | Preparatory actions toward imminent suicidal behavior (Present) | 0.504 |  | **0.791** |  |  |  |  |
| ksads_23_956_t | Self injurious behavior without suicidal intent (Past) | 0.507 |  | **0.447** |  |  |  |  |
| ksads_23_957_t | Suicidal ideation (Passive) (Past) | 0.578 |  | **0.573** |  |  |  |  |
| ksads_23_958_t | Suicidal ideation (Active, nonspecific) (Past) | 0.616 |  | **0.732** |  |  |  |  |
| ksads_23_959_t | Suicidal ideation (Active, method) (Past) | 0.570 |  | **0.762** |  |  |  |  |
| ksads_23_960_t | Suicidal ideation (Active, intent) (Past) | 0.572 |  | **0.732** |  |  |  |  |
| ksads_23_961_t | Suicidal ideation (Active, plan) (Past) | 0.477 |  | **0.802** |  |  |  |  |
| ksads_23_962_t | Preparatory actions toward imminent suicidal behavior (Past) | 0.488 |  | **0.698** |  |  |  |  |
| ksads_23_964_t | Aborted attempt (Past) | 0.561 |  | **0.523** |  |  |  |  |
| ksads_23_965_t | Suicide attempt (Past) | 0.635 |  | **0.489** |  |  |  |  |
| ksads_14_87_p | Difficulty remaining seated for more than one school year (Past) | 0.412 |  |  | **0.804** |  |  |  |
| ksads_14_85_p | Difficulty remaining seated since elementary school (Present) | 0.426 |  |  | **0.712** |  |  |  |
| ksads_14_88_p | Impulsivity (Present) | 0.444 |  |  | **0.679** |  |  |  |
| ksads_14_90_p | Impulsivity for more than one school year (Past) | 0.445 |  |  | **0.772** |  |  |  |
| ksads_15_95_p | Often disobeys rules/requests (Present) | 0.361 |  |  | **0.770** |  |  |  |
| ksads_15_96_p | Often disobeys rules/requests (Past) | 0.338 |  |  | **0.703** |  |  |  |
| ksads_15_94_p | Often argues with adults/authority (Past) | 0.329 |  |  | **0.696** |  |  |  |
| ksads_15_93_p | Often argues with adults/authority (Present) | 0.342 |  |  | **0.734** |  |  |  |
| ksads_16_98_p | Often lies (Present) | 0.361 |  |  | **0.565** |  |  |  |
| ksads_16_99_p | Often lies (Past) | 0.327 |  |  | **0.483** |  |  |  |
| ksads_16_104_p | Often bullies others (Present) | 0.249 |  |  | **0.470** |  |  |  |
| ksads_16_105_p | Often bullies others (Past) | 0.279 |  |  | **0.527** |  |  |  |
| ksads_16_107_p | Stealing (Past) | 0.263 |  |  | **0.530** |  |  |  |
| ksads_16_106_p | Stealing (Present) | 0.290 |  |  | **0.503** |  |  |  |
| ksads_14_79_p* | Difficulty sustaining attention for more than one school year (Past) | 0.428 |  |  | **0.744** |  |  |  |
| ksads_14_83_p* | Easily distracted for more than one school year (Past) |  |  |  |  |  |  |  |
| ksads_14_77_p** | Difficulty sustaining attention since elementary school (Present) | 0.410 |  |  | **0.655** |  |  |  |
| ksads_14_81_p** | Easily distracted since elementary school (Present) |  |  |  |  |  |  |  |
| gen_child_behav_1 | Has your child experienced periods of several days or more when, although he/she was feeling unusually happy and intensely energetic (clearly more than your child's usual self), he/she was also physically restless, unable to sit still, and had to keep moving or jumping from one activity to another? | 0.413 |  |  |  | **0.698** |  |  |
| gen_child_behav_2 | Have there been periods of several days or more when your child's friends or other family members told you that your child seemed unusually happy or high - clearly different from his/her usual self or from a typical good mood? | 0.351 |  |  |  | **0.723** |  |  |
| gen_child_behav_3 | Has your child's mood or energy shifted rapidly back and forth from happy to sad or high to low? | 0.356 |  |  |  | **0.728** |  |  |
| gen_child_behav_4 | Has your child had periods of extreme happiness and intense energy that last several days or more when he/she also felt more anxious or tense (jittery, nervous, uptight) than usual (other than relates to the menstrual cycle)? | 0.376 |  |  |  | **0.774** |  |  |
| gen_child_behav_5 | Have there been times of several days or more when, although your child was feeling unusually happy and intensely energetic (clearly more than his/her usual self), he/she also had to struggle very hard to control inner feelings of rage or an urge to smash or destroy things? | 0.447 |  |  |  | **0.743** |  |  |
| gen_child_behav_6 | Has your child had periods of extreme happiness and intense energy (clearly more than his/her normal self) when, for several days or more, it took him/her over an hour to get to sleep at night? | 0.355 |  |  |  | **0.710** |  |  |
| gen_child_behav_7 | Have you ever found that your child's feelings or energy are generally up or down, but rarely in the middle? | 0.388 |  |  |  | **0.746** |  |  |
| gen_child_behav_8 | Has your child had periods lasting several days or more when he/she felt depressed or irritable, and then other periods of several days or more when he/she felt extremely high, elated, and overflowing with energy? | 0.406 |  |  |  | **0.772** |  |  |
| gen_child_behav_9 | Have there been periods when, although your child was feeling unusually happy and intensely energetic, almost everything got on his/her nerves and made him/her irritable or angry (other than related to the menstrual cycle)? | 0.383 |  |  |  | **0.771** |  |  |
| gen_child_behav_10 | Has your child had times when his/her thoughts and ideas came so fast that he/she couldn't get them all out, or they came so quickly others complained that they couldn't keep up with your child's ideas? | 0.397 |  |  |  | **0.595** |  |  |
| bpm_1_y | "I act too young for my age" | 0.495 |  |  |  |  | **0.066** |  |
| bpm_2_y | "I argue a lot" | 0.619 |  |  |  |  | **0.023** |  |
| bpm_3_y | "I fail to finish things I start" | 0.560 |  |  |  |  | **0.027** |  |
| bpm_4_y | "I have trouble concentrating or paying attention" | 0.772 |  |  |  |  | **-0.201** |  |
| bpm_5_y | "I have trouble sitting still" | 0.643 |  |  |  |  | **-0.152** |  |
| bpm_6_y | "I destroy things belonging to others" | 0.633 |  |  |  |  | **-0.091** |  |
| bpm_7_y | "I disobey my parents" | 0.569 |  |  |  |  | **-0.031** |  |
| bpm_8_y | "I disobey at school" | 0.625 |  |  |  |  | **-0.135** |  |
| bpm_9_y | "I feel worthless or inferior" | 0.688 |  |  |  |  | **0.383** |  |
| bpm_10_y | "I act without stopping to think" | 0.644 |  |  |  |  | **-0.012** |  |
| bpm_11_y | "I am too fearful or anxious" | 0.548 |  |  |  |  | **0.494** |  |
| bpm_12_y | "I feel too guilty" | 0.583 |  |  |  |  | **0.426** |  |
| bpm_13_y | "I am self-conscious or easily embarrassed" | 0.487 |  |  |  |  | **0.439** |  |
| bpm_14_y | "I am inattentive or easily distracted" | 0.755 |  |  |  |  | **-0.152** |  |
| bpm_15_y | "I am stubborn" | 0.474 |  |  |  |  | **0.090** |  |
| bpm_16_y | "I have a hot temper" | 0.652 |  |  |  |  | **0.046** |  |
| bpm_17_y | "I threaten to hurt people" | 0.668 |  |  |  |  | **-0.038** |  |
| bpm_18_y | "I am unhappy, sad, or depressed" | 0.669 |  |  |  |  | **0.328** |  |
| bpm_19_y | "I worry a lot" | 0.563 |  |  |  |  | **0.505** |  |
| poa_nihtb_1_y | "I felt attentive" | -0.229 |  |  |  |  |  | **0.508** |
| poa_nihtb_2_y | "I felt delighted" | -0.128 |  |  |  |  |  | **0.707** |
| poa_nihtb_3_y | "I felt calm" | -0.293 |  |  |  |  |  | **0.652** |
| poa_nihtb_4_y | "I felt at ease" | -0.234 |  |  |  |  |  | **0.715** |
| poa_nihtb_5_y | "I felt enthusiastic" | -0.037 |  |  |  |  |  | **0.767** |
| poa_nihtb_6_y | "I felt interested" | -0.090 |  |  |  |  |  | **0.723** |
| poa_nihtb_7_y | "I felt confident" | -0.212 |  |  |  |  |  | **0.735** |
| poa_nihtb_8_y | "I felt energetic" | -0.021 |  |  |  |  |  | **0.702** |
| poa_nihtb_9_y | "I felt able to concentrate" | -0.453 |  |  |  |  |  | **0.574** |
| * Items combined for purposes of factor analysis | | | | | | | | |
| ** Items combined for purposes of factor analysis | | | | | | | | |

Results of the confirmatory bifactor analysis of psychopathology items in the ABCD Study. All items from exploratory factor analysis were retained. In addition to displaying within-factor loadings for each item, loadings to a general psychopathology (P-) factor derived from the bifactor modeling are displayed.

**Supplemental Table 17.** Final confirmatory bifactor analysis of optimized collection of ABCD exposome variables

| Item | Components | | General | F1 | F2 | F3 | F4 | F5 | F6 |
| --- | --- | --- | --- | --- | --- | --- | --- | --- | --- |
| Severe family poverty (e.g., inability to afford necessities) | demo_fam_exp1_v2_l | Inability to afford food or pay to go get it | 0.606 | 0.213 | 0.132 |  |  |  |  |
|  | demo_fam_exp2_v2_l | Inability to afford telephone service |  |  |  |  |  |  |  |
|  | demo_fam_exp3_v2_l | Inability to pay full amount of rent or mortgage |  |  |  |  |  |  |  |
|  | demo_fam_exp4_v2_l | Eviction due to inability to afford rent or mortgage |  |  |  |  |  |  |  |
|  | demo_fam_exp5_v2_l | Loss of utility services due to inability to pay gas, electric, or oil company |  |  |  |  |  |  |  |
| Family legal trouble (e.g., arrests, jailtime) | ple_jail_y | One of the parents/caregivers went to jail | 0.546 | 0.220 |  | 0.186 |  |  |  |
|  | ple_arrest_y | Someone in the family was arrested |  |  |  |  |  |  |  |
|  | ple_law_y | Parents/caregivers got into trouble with the law |  |  |  |  |  |  |  |
|  | ple_sud_y | Family member had drug and/or alcohol problem |  |  |  |  |  |  |  |
| Planned pregnancy | devhx_6_p | Was your pregnancy with this child a planned pregnancy? | -0.545 | -0.231 | -0.086 |  |  |  |  |
| Inability to afford necessary medical/dental visit | demo_fam_exp6_v2_l | Inability to afford a needed visit to a doctor or to the hospital | 0.539 | 0.208 |  |  |  |  |  |
|  | demo_fam_exp7_v2_l | Inability to afford a needed visit to the dentist |  |  |  |  |  |  |  |
| Prenatal exposure to tobacco or marijuana | devhx_8_tobacco | Tobacco use before knowing of pregnancy | 0.427 | **0.587** |  |  |  |  |  |
|  | devhx_9_tobacco | Tobacco use knowing of pregnancy |  |  |  |  |  |  |  |
|  | devhx_8_marijuana | Marijuana use before knowing of pregnancy |  |  |  |  |  |  |  |
|  | devhx_9_marijuana | Marijuana use knowing of pregnancy |  |  |  |  |  |  |  |
| Physical conflict among adults at the home | ksads_ptsd_raw_766_p | Witnessed the grownups in the home push, shove, or hit one another | 0.414 | **0.507** |  |  |  |  |  |
| Parental separation | ple_separ_y | Parents separated or divorced | 0.414 | 0.298 |  |  |  |  |  |
|  | ple_step_y | Child got new stepmother or stepfather |  |  |  |  |  |  |  |
| Parental lifestyle issues (e.g., trouble with holding job, police, alcohol use) | famhx_ss_fath_prob_dprs_p | Father depression problem | 0.400 | **0.613** |  |  |  |  |  |
|  | famhx_ss_moth_prob_dprs_p | Mother depression problem |  |  |  |  |  |  |  |
|  | famhx_ss_moth_prob_trb_p | Mother trouble with holding job/fighting/police |  |  |  |  |  |  |  |
|  | famhx_ss_fath_prob_trb_p | Father trouble with holding job/fighting/police |  |  |  |  |  |  |  |
|  | famhx_ss_moth_prob_alc_p | Mother alcohol problem |  |  |  |  |  |  |  |
|  | famhx_ss_fath_prob_alc_p | Father alcohol problem |  |  |  |  |  |  |  |
| Enforced family rules for smoking cigarettes | parent_rules_q5 | Are these the same rules [for smoking cigarettes] for all family members? | -0.351 | -0.320 |  |  |  |  |  |
| Caregiver psychopathology (e.g., mood, personality, attention disorders)* | asr_scr_depress_t | Depressive Problems ASR DSM-5-Oriented Scale (t-score) | 0.213 | 0.394 |  |  |  |  |  |
|  | asr_scr_anxdisord_t | Anxiety Problems ASR DSM-5-Oriented Scale (t-score) |  |  |  |  |  |  |  |
|  | asr_scr_somaticpr_t | Somatic Problems ASR DSM-5-Oriented Scale (t-score) |  |  |  |  |  |  |  |
|  | asr_scr_avoidant_t | Avoidant Personality Problems ASR DSM-5-Oriented Scale (t-score) |  |  |  |  |  |  |  |
|  | asr_scr_antisocial_t | Antisocial Personality Problems ASR DSM-5-Oriented Scale (t-score) |  |  |  |  |  |  |  |
|  | asr_scr_inattention_t | Inattention ASR DSM-5-Oriented Scale (t-score) |  |  |  |  |  |  |  |
|  | asr_scr_hyperactive_t | Hyperactivity-Impulsivity ASR DSM-5-Oriented Scale (t-score) |  |  |  |  |  |  |  |
| Prenatal exposure to hard drugs (e.g., cocaine, heroin) | devhx_8_coc_crack | Cocaine/Crack use before knowing of pregnancy | 0.201 | **0.596** |  |  |  |  |  |
|  | devhx_8_her_morph | Heroin/Morphine before knowing of pregnancy |  |  |  |  |  |  |  |
|  | devhx_8_oxycont | Oxycontin use before knowing of pregnancy |  |  |  |  |  |  |  |
|  | devhx_8_other_drugs | Any other drug use before knowing of pregnancy |  |  |  |  |  |  |  |
|  | devhx_9_coc_crack | Cocaine/Crack use knowing of pregnancy |  |  |  |  |  |  |  |
|  | devhx_9_her_morph | Heroin/Morphine use knowing of pregnancy |  |  |  |  |  |  |  |
|  | devhx_9_oxycont | Oxycontin use knowing of pregnancy |  |  |  |  |  |  |  |
|  | devhx_9_other_drugs | Any other drug use knowing of pregnancy |  |  |  |  |  |  |  |
| Parent-reported sexual abuse | ksads_ptsd_raw_767_p | A grown up in the home touched the child in his or her privates, had the child touch their privates, or did other sexual things to the child | 0.200 | 0.372 |  |  |  |  |  |
|  | ksads_ptsd_raw_768_p | An adult outside the family touched the child in his or her privates, had the child touch their privates or did other sexual things to the child |  |  |  |  |  |  |  |
|  | ksads_ptsd_raw_769_p | A peer forced the child to do something sexually |  |  |  |  |  |  |  |
| Sudden death of a loved one | ksads_ptsd_raw_770_p | Learned about the sudden unexpected death of a loved one | 0.191 | 0.258 |  |  |  |  |  |
| Prenatal exposure to alcohol | devhx_8_alcohol | Alcohol use before knowing of pregnancy | -0.165 | 0.457 | -0.041 |  |  |  |  |
|  | devhx_9_alcohol | Alcohol use knowing of pregnancy |  |  |  |  |  |  |  |
| Severe maternal mental health issues (e.g., breakdowns, delusions, hospitalizations) | famhx_ss_moth_prob_dg_p | Mother drug use problem | 0.132 | **0.514** |  |  |  |  |  |
|  | famhx_ss_moth_prob_hspd_p | Mother hospitalized due to emotional/mental problem |  |  |  |  |  |  |  |
|  | famhx_ss_moth_prob_ma_p | Mother mania problem |  |  |  |  |  |  |  |
|  | famhx_ss_moth_prob_nrv_p | Mother nerves/nervous breakdown problem |  |  |  |  |  |  |  |
|  | famhx_ss_moth_prob_prf_p | Mother been to a doctor or counselor due to emotional/mental problem |  |  |  |  |  |  |  |
|  | famhx_ss_moth_prob_scd_p | Mother attempted or committed suicide |  |  |  |  |  |  |  |
|  | famhx_ss_moth_prob_vs_p | Mother visions of others spying/plotting problem |  |  |  |  |  |  |  |
| Parent-reported childhood trauma (e.g., accident, disaster, extreme violence) | ksads_ptsd_raw_754_p | Car accident in which your child or another person in the car was hurt bad enough to require medical attention | 0.079 | 0.142 |  |  |  |  |  |
|  | ksads_ptsd_raw_755_p | A significant accident for which the child needed specialized and intensive medical treatment |  |  |  |  |  |  |  |
|  | ksads_ptsd_raw_756_p | Witnessed or caught in a fire that caused significant property damage or personal injury |  |  |  |  |  |  |  |
|  | ksads_ptsd_raw_757_p | Witnessed or caught in a natural disaster that caused significant property damage or personal injury |  |  |  |  |  |  |  |
|  | ksads_ptsd_raw_758_p | Witnessed or present during an act of terrorism (e.g., Boston marathon bombing) |  |  |  |  |  |  |  |
|  | ksads_ptsd_raw_759_p | Witnessed death or mass destruction in a war zone |  |  |  |  |  |  |  |
|  | ksads_ptsd_raw_760_p | Witnessed someone shot or stabbed in the community |  |  |  |  |  |  |  |
|  | ksads_ptsd_raw_761_p | Shot, stabbed, or beaten brutally by a non-family member |  |  |  |  |  |  |  |
|  | ksads_ptsd_raw_762_p | Shot, stabbed, or beaten brutally by a grown up in the home |  |  |  |  |  |  |  |
|  | ksads_ptsd_raw_763_p | Beaten to the point of having bruises by a grown up in the home |  |  |  |  |  |  |  |
|  | ksads_ptsd_raw_764_p | A non-family member threatened to kill the child |  |  |  |  |  |  |  |
|  | ksads_ptsd_raw_765_p | A family member threatened to kill the child |  |  |  |  |  |  |  |
| Severe paternal mental health issues (e.g., breakdowns, delusions, hospitalizations) | famhx_ss_fath_prob_dg_p | Father drug use problem | 0.058 | 0.402 |  |  |  |  |  |
|  | famhx_ss_fath_prob_hspd_p | Father hospitalized due to emotional/mental problem |  |  |  |  |  |  |  |
|  | famhx_ss_fath_prob_ma_p | Father mania problem |  |  |  |  |  |  |  |
|  | famhx_ss_fath_prob_nrv_p | Father nerves/nervous breakdown problem |  |  |  |  |  |  |  |
|  | famhx_ss_fath_prob_prf_p | Father been to a doctor or counselor due to emotional/mental problem |  |  |  |  |  |  |  |
|  | famhx_ss_fath_prob_scd_p | Father attempted or committed suicide |  |  |  |  |  |  |  |
|  | famhx_ss_fath_prob_vs_p | Father visions of others spying/plotting problem |  |  |  |  |  |  |  |
| Census-derived neighborhood poverty (e.g., unemployment rate, families/individuals below poverty level) | reshist_addr1_adi_in_dis | Income disparity (log of 100 x ratio of the number of households with <10000 annual income to the number of households with >50000 annual income) | 0.529 |  | **0.584** |  |  |  |  |
|  | reshist_addr1_adi_ncar | Percentage of occupied housing units without a motor vehicle |  |  |  |  |  |  |  |
|  | reshist_addr1_adi_home_o | Percentage of house ownership |  |  |  |  |  |  |  |
|  | reshist_addr1_leadrisk_poverty | Percentage of individuals below -125% of the poverty level |  |  |  |  |  |  |  |
|  | reshist_addr1_adi_pov | Percentage of families below the poverty level |  |  |  |  |  |  |  |
|  | reshist_addr1_adi_sp | Percentage of single owners/tenants |  |  |  |  |  |  |  |
|  | reshist_addr1_adi_unemp | Unemployment rate (percentage of civilian labor force population above 16 and unemployed) |  |  |  |  |  |  |  |
|  | reshist_addr1_adi_ntel | Percentage of occupied housing units without a telephone |  |  |  |  |  |  |  |
|  | reshist_addr1_adi_nplumb | Percentage of occupied housing units without complete plumbing |  |  |  |  |  |  |  |
| Parent-reported neighborhood safety | nsc_p_ss_mean_3_items | Neighborhood Safety Protocol (mean of parent report) | -0.358 |  | -0.433 |  |  |  |  |
| Census-derived neighborhood immigration and crowding | reshist_addr1_adi_crowd | Percentage of occupied housing units with more than 1 person per room (crowding) | 0.294 |  | **0.505** |  |  |  |  |
|  | reshist_addr1_adi_edu_l | Percentage of population over 25 with less than 9 years of education |  |  |  |  |  |  |  |
|  | reshist_addr1_adi_work_c | Percentage of employed persons above 16 in white collar occupations |  |  |  |  |  |  |  |
| Census-derived neighborhood lead exposure risk | reshist_addr1_leadrisk_housing | Estimated percentage of homes at risk for lead exposure given lead-based paint | 0.254 |  | **0.517** |  |  | -0.141 |  |
|  | reshist_addr1_leadrisk | Estimated lead risk (1-10 scale) |  |  |  |  |  |  |  |
| Census-derived neighborhood air pollution (NO2,PM25) | reshist_addr1_no2 | 3 years average of ground level NO2 at 10x10km2 | 0.158 |  | 0.393 |  |  |  |  |
|  | reshist_addr1_pm25 | Annual average of PM 2.5 at 10x10km2 |  |  |  |  |  |  |  |
|  | reshist_addr1_pm252016aa | Annual average of PM 2.5 at 1x1km2 |  |  |  |  |  |  |  |
| Parental ability to speak English | accult_q1_p | How well do you speak English? | -0.141 | 0.321 | **-0.564** |  |  |  |  |
| Census-derived neighborhood population density | reshist_addr1_d1a | Gross residential density | 0.074 |  | **0.779** |  |  |  |  |
|  | reshist_addr1_popdensity | UN adjusted population density |  |  |  |  |  |  |  |
| Census-derived neighborhood walkability index | reshist_addr1_walkindex | National walkability index | 0.023 |  | **0.606** |  |  |  |  |
| Youth-reported racial/ethnic discrimination (past year) | dim_yesno_q1 | In the past 12 months, have you felt discriminated against because of your race, ethnicity, or color? | 0.516 |  |  | 0.288 |  |  |  |
| Youth-reported lesbian, gay, bisexual discrimination (past year) | dim_yesno_q3 | In the past 12 months, have you felt discriminated against because someone thought you were gay, lesbian, or bisexual? | 0.429 |  |  | 0.289 |  |  |  |
| Youth-reported discrimination based on weight (past year) | dim_yesno_q4 | In the past 12 months, have you felt discriminated against because of your weight? | 0.401 |  |  | 0.298 |  |  |  |
| Youth-reported neighborhood safety | neighborhood_crime_y | "My neighborhood is safe from crime" | -0.369 |  | -0.230 | -0.274 |  |  |  |
| Youth-reported discrimination based on being foreign (past year) | dim_yesno_q2 | In the past 12 months, have you felt discriminated against because you are (or your family is) from another country? | 0.322 |  | 0.138 | 0.187 |  |  |  |
| Youth-reported unfair treatment on racial/ethnic grounds (lifetime) | dim_matrix_q1 | How often do teachers treat you unfairly or negatively because of your ethnic background? | 0.320 |  |  | 0.264 |  |  |  |
|  | dim_matrix_q2 | How often do other adults outside school treat you unfairly or negatively because of your ethnic background? |  |  |  |  |  |  |  |
|  | dim_matrix_q3 | How often do other students treat you unfairly or negatively because of your ethnic background? |  |  |  |  |  |  |  |
|  | dim_matrix_q4 | "I feel that others behave in an unfair or negative way toward my ethnic group" |  |  |  |  |  |  |  |
| Youth-reported family discordance (e.g., loss of job, mental health issues, conflict/violence) | ple_financial_y | Negative change in parent's financial situation | 0.280 |  |  | 0.253 |  |  |  |
|  | ple_argue_y | Parents argued more than previously |  |  |  |  |  |  |  |
|  | ple_job_y | Mother/father figure lost job |  |  |  |  |  |  |  |
|  | ple_away_y | One parent was away from home more often |  |  |  |  |  |  |  |
|  | ple_mh_y | Family member had mental/emotional problem |  |  |  |  |  |  |  |
|  | ple_victim_y | Child was a victim of crime/violence/assault |  |  |  |  |  |  |  |
|  | ple_sib_y | Brother or sister left home |  |  |  |  |  |  |  |
| Youth-reported exposure to serious injury, illness, death (self or other) | ple_friend_died_y | Child’s close friend died | 0.277 |  |  | 0.180 |  |  |  |
|  | ple_friend_y | Child lost a close friend |  |  |  |  |  |  |  |
|  | ple_friend_injur_y | Child’s close friend was seriously sick/injured |  |  |  |  |  |  |  |
|  | ple_ill_y | Child was seriously sick |  |  |  |  |  |  |  |
|  | ple_injured_y | Family member was seriously injured |  |  |  |  |  |  |  |
|  | ple_injur_y | Child was seriously injured |  |  |  |  |  |  |  |
|  | ple_crime_y | Child saw crime or accident |  |  |  |  |  |  |  |
|  | ple_died_y | Someone in family died |  |  |  |  |  |  |  |
| Youth-reported family conflict | fes_y_ss_fc | Conflict subscale from the Family Environment Scale (raw score) | 0.247 |  |  | 0.422 |  |  |  |
| Youth-reported parental monitoring and communication | parent_monitor_q1_y | How often do your parents/guardians know where you are? | -0.210 |  |  | **-0.526** |  |  |  |
|  | parent_monitor_q2_y | How often do your parents know who you are with when you are not at school and away from home? |  |  |  |  |  |  |  |
|  | parent_monitor_q3_y | If you are at home when your parents or guardians are not, how often do you know how to get in touch with them? |  |  |  |  |  |  |  |
|  | parent_monitor_q4_y | How often do you talk to your mom/dad or guardian about your plans for the coming day, such as your plans about what will happen at school or what you are going to do with friends? |  |  |  |  |  |  |  |
|  | parent_monitor_q5_y | In an average week, how many times do you and your parents/guardians, eat dinner together? |  |  |  |  |  |  |  |
| Youth-reported positive school involvement | school_2_y | “In my school, students have lots of chances to help decide things like class activities and rules” | -0.156 |  |  | **-0.592** |  |  |  |
|  | school_5_y | “There are lots of chances for students in my school to get involved in sports, clubs, or other school activities outside of class” |  |  |  |  |  |  |  |
|  | school_6_y | “I feel safe at my school” |  |  |  |  |  |  |  |
|  | school_10_y | “There are lots of chances to be part of class discussions or activities” |  |  |  |  |  |  |  |
| Youth-reported acceptance and love by primary caregiver | crpbi_y_ss_parent | CRPBI Acceptance subscale (mean of primary caregiver report by youth) | -0.153 |  |  | **-0.584** |  |  |  |
| Youth-reported school enjoyment | school_3_y | “I get along with my teachers” | -0.147 |  |  | **-0.559** |  |  |  |
|  | school_12_y | “In general, I like school a lot” |  |  |  |  |  |  |  |
|  | school_15_y | “Usually, school bores me” |  |  |  |  |  |  |  |
| Youth-reported acceptance and love by secondary caregiver | crpbi_y_ss_caregiver | CRPBI Acceptance subscale (mean of secondary caregiver report by youth) | -0.099 |  |  | **-0.515** |  |  |  |
| Youth-reported positive feedback at school | school_4_y | “My teacher(s) notices when I am doing a good job and lets me know about it” | 0.037 |  |  | **-0.546** |  |  |  |
|  | school_7_y | “The school lets my parents know when I have done something well” |  |  |  |  |  |  |  |
| Youth-reported school grades and achievement | school_8_y | “I like school because I do well in class” | -0.012 |  |  | **-0.588** |  |  |  |
|  | school_9_y | “I feel I'm just as smart as other kids my age” |  |  |  |  |  |  |  |
|  | school_17_y | “Getting good grades is not so important to me” |  |  |  |  |  |  |  |
| Census-derived neighborhood wealth (e.g., median mortgage, rent, income) | reshist_addr1_adi_home_v | Median home value | -0.594 | -0.007 |  |  | -0.314 |  |  |
|  | reshist_addr1_adi_mortg | Median monthly mortgage |  |  |  |  |  |  |  |
|  | reshist_addr1_adi_rent | Median gross rent |  |  |  |  |  |  |  |
|  | reshist_addr1_adi_income | Median family income |  |  |  |  |  |  |  |
| State-level marijuana laws | reshist_state_mj_laws | Marijuana state law during the same year as the assessment | 0.237 |  |  |  | **0.707** |  |  |
| State-level indicators of racism | reshist_state_racism_factor | State level indicators of racism from survey and implicit bias measures and state level structural variables | 0.228 |  |  |  | **0.659** |  |  |
| State-level indicators of bias against immigrants | reshist_state_immigrant_factor | State level indicators of immigrant bias from survey and implicit bias measures and state level structural variables | 0.202 |  | -0.347 |  | **0.618** |  |  |
| State-level indicators bias against sexual orientation | reshist_state_so_factor | State level indicators of bias against sexual orientation from structural variables | 0.179 |  | -0.297 |  | **0.875** |  |  |
| State-level indicators of sexism | reshist_state_sexism_factor | State level indicators of sexism from survey and implicit bias measures | 0.138 |  |  |  | **0.843** |  |  |
| State-level legality of medical marijuana | su_risk_p_6 | Is "medical marijuana" (marijuana prescribed by a doctor) legal in your state? | 0.092 |  |  |  | **0.701** |  |  |
| Parent-reported importance of coherence to the family unit | macv_p_ss_fr | MACVS Family as Referent subscale (mean) | 0.229 |  | 0.145 |  |  | 0.465 |  |
| Parent-reported importance of religion | macv_p_ss_r | MACVS Religion subscale (mean) | 0.189 |  | 0.121 |  | 0.267 | 0.427 |  |
| Parent-reported importance of obligation to family | macv_p_ss_fo | MACVS Family Obligation subscale (mean) | 0.177 |  | 0.149 |  |  | 0.436 |  |
| Family rules for drinking alcohol | parent_rules_q1 | What are the family rules about drinking alcohol for your son/daughter? | 0.129 |  |  |  |  | **0.908** |  |
| Family rules for using marijuana | parent_rules_q7 | What are the family rules about using marijuana for your son/daughter? | 0.085 |  |  |  |  | **0.941** |  |
| Parent-reported importance of family support | macv_p_ss_fs | MACVS Family Support subscale (mean) | 0.058 |  | 0.113 |  |  | 0.454 |  |
| Family rules for smoking cigarettes | parent_rules_q4 | What are the family rules about smoking cigarettes for your son/daughter? | 0.012 |  |  |  |  | **0.953** |  |
| Family religiosity (e.g., attendance to religious services) | demo_yrs_1_l | Frequency of child’s attendance to religious services | 0.008 |  |  |  | 0.258 | 0.342 |  |
|  | demo_yrs_2_l | Parental importance of child's religious and spiritual beliefs |  |  |  |  |  |  |  |
| Twin brother or sister | devhx_5_p | Does your child have a twin? | -0.217 |  |  |  |  |  | **0.772** |
| Amount of prenatal care | devhx_11_p | About how many times did you/biological mother see a doctor or other medical professional for prenatal care during this pregnancy? | -0.107 |  |  |  |  |  | 0.388 |
| Blood pressure complications during pregnancy (e.g., pregnancy-related high blood pressure, diabetes) | devhx_10j3_p | Pregnancy-related high blood pressure | 0.102 |  |  |  |  |  | 0.420 |
|  | devhx_10c3_p | Pre-eclampsia, eclampsia, or toxemia during pregnancy |  |  |  |  |  |  |  |
|  | devhx_10i3_p | Pregnancy-related diabetes |  |  |  |  |  |  |  |
| Circulation complications at birth (e.g., blue, slow heartbeat at birth) | devhx_14a3_p | Blue at birth | 0.076 |  |  |  |  |  | 0.357 |
|  | devhx_14b3_p | Slow heartbeat at birth |  |  |  |  |  |  |  |
|  | devhx_14c3_p | Did not breathe at first at birth |  |  |  |  |  |  |  |
| Premature birth | devhx_12a_p | Was the child born prematurely? | -0.050 |  |  |  |  |  | **0.856** |
| Time after birth in an incubator | devhx_15 | For how many days after birth was the child in an incubator? | 0.049 |  |  |  |  |  | **0.537** |
| Placental complications during pregnancy (e.g., previa, abruptio, persistent proteinuria) | devhx_10k3_p | Previa, abruptio, or other problems with the placenta during pregnancy | -0.043 |  |  |  |  |  | 0.454 |
|  | devhx_10f3_p | Rubella (German measles) during first 3 months of pregnancy |  |  |  |  |  |  |  |
|  | devhx_10b3_p | Heavy bleeding requiring bed rest or special treatment during pregnancy |  |  |  |  |  |  |  |
|  | devhx_10e3_p | Persistent proteinuria during pregnancy |  |  |  |  |  |  |  |
|  | devhx_10m3_p | Any other conditions requiring medical care during pregnancy |  |  |  |  |  |  |  |
|  | devhx_10l3_p | Accident or injury requiring medical care during pregnancy |  |  |  |  |  |  |  |
| Birth by caesarian section | devhx_13_3_p | Was the child born by Caesarian section? | -0.042 |  |  |  |  |  | 0.477 |
| Blood oxygen complications at birth (e.g., jaundice, supplemental oxygen) | devhx_14e3_p | Did the child have jaundice needing treatment at birth? | -0.014 |  |  |  |  |  | **0.649** |
|  | devhx_14f3_p | Did the child require oxygen at birth? |  |  |  |  |  |  |  |
| * Report of whichever caregiver accompanied child to ABCD assessment | | | | | | | | | |

Results of confirmatory bifactor analysis of the final set of exposome items in the ABCD Study, using iterated target rotation designed to detect complex structure (cross-loadings). Only items with significant within-factor loading were retained, narrowing the set of total items to 65. In addition to displaying significant within- and between-factor loadings for each item, loadings to a general exposome (E) factor derived from bifactor modeling are displayed. Columns B and C break down each item into its constituent ABCD variables. See **Supplemental Table 3** for more information about full ABCD measures.

**Supplemental Table 18.** Association of ABCD exposome factor scores with obesity

|  | **OR** | **95% CI** | ***p*-value** |
| --- | --- | --- | --- |
| **General exposome adversity** | 1.410 | 1.306-1.522 | <0.001 |
| **Household adversity** | 1.000 | 0.946-1.056 | 0.990 |
| **Neighborhood environment** | 1.050 | 0.984-1.120 | 0.139 |
| **Day-to-day experiences** | 1.004 | 0.951-1.061 | 0.874 |
| **State environment** | 0.946 | 0.892-1.003 | 0.061 |
| **Family values** | 0.995 | 0.940-1.053 | 0.864 |
| **Pregnancy/birth complications** | 1.025 | 0.969-1.084 | 0.384 |

ABCD exposome factor score associations to obesity, defined in accordance with the CDC as being above the 95^th^ percentile of BMI, are derived from a binary logistic regression model. Model covaries for age, sex, race (White, Black, with “other” as reference), and ethnicity (Hispanic).

Abbreviations: OR = odds ratio; CI = confidence interval; CDC = Centers for Disease Control; BMI = body mass index.

**Supplemental Table 19.** Association of ABCD exposome factor scores with late/post-pubertal status

|  | **OR** | **95% CI** | ***p*-value** |
| --- | --- | --- | --- |
| **General exposome adversity** | 1.304 | 1.156-1.472 | <0.001 |
| **Household adversity** | 1.009 | 0.922-1.104 | 0.841 |
| **Neighborhood environment** | 1.012 | 0.910-1.126 | 0.824 |
| **Day-to-day experiences** | 1.305 | 1.194-1.426 | <0.001 |
| **State environment** | 1.023 | 0.931-1.124 | 0.639 |
| **Family values** | 0.991 | 0.902-1.088 | 0.842 |
| **Pregnancy/birth complications** | 0.952 | 0.870-1.042 | 0.284 |

ABCD exposome factor score associations to youth-reported belonging to late or post-pubertal status are derived from a binary logistic regression model. Model covaries for age, sex, race (White, Black, with “other” as reference), Hispanic ethnicity and BMI.

Abbreviations: OR = odds ratio; CI = confidence interval; BMI = body mass index.

**Supplemental Table 28.** Final confirmatory bifactor analysis of PNC exposome variables

| Item | Item description | General | F1 | F2 | F3 | F4 |
| --- | --- | --- | --- | --- | --- | --- |
| Ran_psychosis_FH | Family history of psychosis | 0.25 | **0.55** |  |  |  |
| Parents_Sep_Divorce | Parental separation or divorce | 0.20 | **0.65** |  |  |  |
| Ran_Sui_attempt_or_death_FH | Family history of attempted or completed suicide | 0.19 | **0.70** |  |  |  |
| Ran_substance_FH | Family history of substance abuse | 0.19 | **0.58** |  |  |  |
| Ran_bipolar_or_lithium_FH | Family history of bipolar disorder/lithium prescription | 0.10 | **0.61** |  |  |  |
| Ran_depression_FH | Family history of depression | -0.05 | 0.33 |  |  |  |
| PercentMarried | Percentage of married residents in neighborhood | -0.90 |  | 0.08 |  |  |
| PercentInPoverty | Percentage of neighborhood residents in poverty | 0.86 |  | 0.20 |  |  |
| MedianFamilyIncome | Neighborhood median family income | -0.83 |  | -0.07 |  |  |
| PercentHighSchoolPlus | Percentage of neighborhood residents with a high school degree or higher education | -0.73 |  | -0.17 |  |  |
| PopDensity | Neighborhood population density | 0.71 |  | 0.10 |  |  |
| PerVac | Percentage of vacant lots in neighborhood | 0.59 |  | -0.11 |  |  |
| PercentEmployed | Percentage of employed neighborhood residents | -0.55 |  | -0.44 |  |  |
| MED_AGE | Median age of neighborhood residents | -0.53 |  | -0.35 |  |  |
| PercentFemale | Percentage of female residents in neighborhood | 0.28 |  | -0.09 |  |  |
| PercentNonfamilyHouseholds | Percentage of non-family households in neighborhood | 0.22 |  | -0.30 |  |  |
| PercentWithChildren | Percentage of neighborhood residents with children | -0.20 |  | **0.75** |  |  |
| PercentEnglishSpeakers | Percentage of English-speaking neighborhood residents | -0.19 |  | -0.40 |  |  |
| AvgHouseholdSize | Average neighborhood household size | -0.06 |  | 0.47 |  |  |
| ptd006 | Have you ever been threatened with a weapon? | 0.29 |  |  | **0.73** |  |
| ptd008 | Other than television or at the movies, have you ever seen or heard somebody get killed…or get hurt very badly...or die? | 0.29 |  |  | **0.65** |  |
| ptd003 | Have you ever been attacked by somebody...or badly beaten? | 0.23 |  |  | **0.69** |  |
| ptd009 | Have you ever been very upset by seeing a dead body...or by seeing pictures of the dead body of somebody you knew well? | 0.22 |  |  | **0.70** |  |
| ptd002 | Have you ever been in a situation where you thought you or someone close to you was going to be killed...or be hurt very badly (e.g., family violence)? | 0.20 |  |  | **0.71** |  |
| ptd004 | Have you ever been very upset by someone forcing you to do something sexual? | 0.13 |  |  | 0.49 |  |
| ptd007 | Have you ever been in a bad accident? | 0.08 |  |  | 0.45 |  |
| ptd001 | Have you ever been in a flood…or a tornado…or an earthquake…or a hurricane…or some other natural disaster where you thought you were going to die or be seriously hurt? | 0.01 |  |  | 0.37 |  |
| MED242 | Do/did you ever have lead poisoning? | 0.35 |  |  |  | 0.28 |
| MED070 | Was your birth (that is, while your mom was carrying you, or the time of your delivery) abnormal in any way? This includes premature or overdue births. | -0.10 |  |  |  | 0.28 |

Results of confirmatory bifactor analysis of the set of exposome items in the PNC. All items are included. *Factor 1* comprises variables that are broadly related to *household adversity* and includes first degree family history of mental health issues and parental separation/divorce. *Factor 2* comprises variables most related to *neighborhood environment*, informed by census-derived measures. *Factor 3* comprises variables related to *trauma exposure*. *Factor 4* comprises two variables most related to *early life*, including birth complications and lead exposure. In addition to displaying significant within-factor loadings for each item, loadings to a general exposome factor (*Exp-factor*) derived from bifactor modeling are displayed. Column B describes each item included from the PNC dataset.

**Supplemental Table 29.** Association of PNC exposome factor scores with obesity

|  | **OR** | **95% CI** | ***p*-value** |
| --- | --- | --- | --- |
| **General exposome adversity** | 1.434 | 1.274-1.613 | <0.001 |
| **Household adversity** | 1.097 | 1.011-1.191 | 0.026 |
| **Neighborhood environment** | 1.003 | 0.926-1.087 | 0.941 |
| **Trauma exposure** | 1.088 | 0.982-1.205 | 0.105 |
| **Early life** | 0.935 | 0.861-1.015 | 0.109 |

PNC exposome factor score associations with obesity (binary variable, BMI>=95^th^ percentile) are derived from a linear regression model. Model covaries for age, sex, race (White, Black, with “other” as reference), and Hispanic ethnicity.

Abbreviations: PNC= Philadelphia Neurodevelopmental Cohort; BMI = body mass index; OR = odds ratio; CI = confidence interval.

**Supplemental Table 30.** Association of PNC exposome factor scores with late/post-pubertal status^a^

|  | **OR** | **95% CI** | ***p*-value** |
| --- | --- | --- | --- |
| **General exposome adversity** | 1.264 | 1.003-1.592 | 0.047 |
| **Household adversity** | 1.063 | 0.906-1.247 | 0.454 |
| **Neighborhood environment** | 1.124 | 0.965-1.310 | 0.133 |
| **Trauma exposure** | 0.881 | 0.712-1.090 | 0.244 |
| **Early life** | 1.127 | 0.961-1.322 | 0.142 |

^a^For models testing associations with pubertal measures, the PNC sample was limited to age range 10-12 to minimize age effects on models. Sample included n=1496, of whom 271 were at late/post pubertal status.

PNC exposome factor score associations to youth-reported belonging to late or post-pubertal status are derived from a binary logistic regression model. Model covaries for age, sex, race (White, Black, with “other” as reference), Hispanic ethnicity and BMI.

Abbreviations: PNC= Philadelphia Neurodevelopmental Cohort; OR = odds ratio; CI = confidence interval; BMI = body mass index.

**Supplemental Figure 1.** Schematic illustration of dimensionality reduction pipeline of environmental exposures in ABCD Study


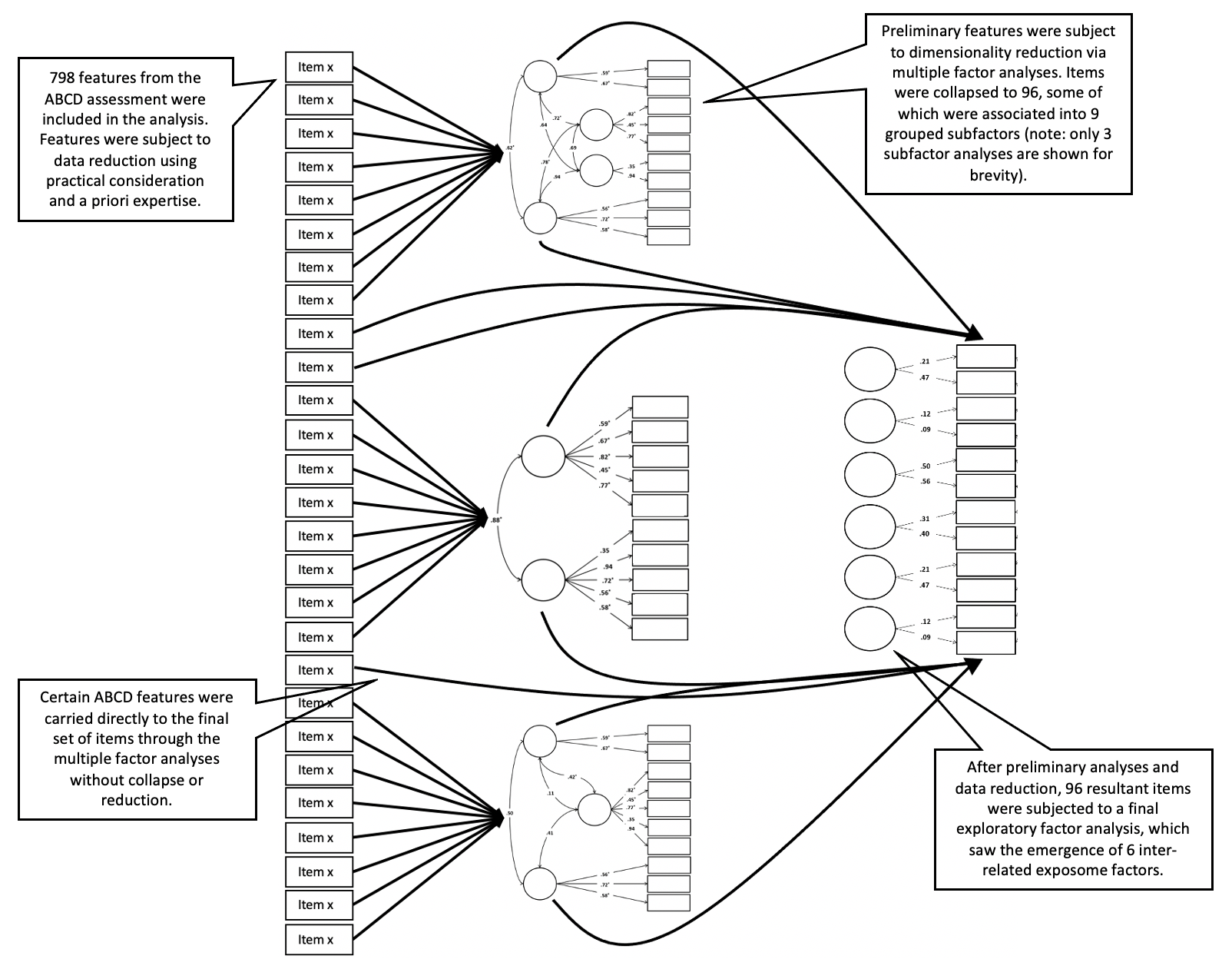


In total, 348 ABCD items were included in the analysis (see **Supplemental Table 3**). From these 348 variables, dimensionality reduction was performed using multiple factor analyses, resulting in 96 combined items. Some of these items emerged as associated subfactors (see **Supplemental Tables 5–13**). This set of 96 items was run through a final exploratory analysis, which separated them into six distinct factors: *household adversity*, *day-to-day experiences*, *neighborhood environment*, *state environment*, *family values*, and *pregnancy/birth complications* (see **Table 1** for full details). Subsequently, the results of this exploratory factor analysis were used for the subsequent bifactor model that was used for downstream analyses (see **Supplemental Table 17**).

**Supplemental Figure 2.** ABCD psychopathology bifactor model

Bifactor model of confirmatory factor analysis in the ABCD Study. Only the top 3 items loading within-factor and on the general *P-factor* are included. Arrow thickness relates to the strength of the loading (higher the loading, thicker the arrow). Arrow color relates to the sign of the loading – a red arrow corresponds to positive loading (associated with a higher *Exp-factor* score; risk factor) and a green arrow corresponds to negative loading (associated with a lower *Exp-factor* score; protective factor). Subfactors are presented from top to bottom in order from F1 to F6. See **Supplemental** **Table 16** for full list of items’ loadings for the ABCD psychopathology bifactor model.

**Supplemental Figure 3.** PNC exposome bifactor model

Bifactor model of confirmatory factor analysis in PNC. All items are included. Arrow thickness relates to the strength of the loading (higher the loading, thicker the arrow). Arrow color relates to the sign of the loading – a red arrow corresponds to positive loading (associated with a higher *Exp-factor* score; risk factor) and a green arrow corresponds to negative loading (associated with a lower *Exp-factor* score; protective factor). Subfactors are presented from top to bottom in order from F1 to F4. See **Supplemental** **Table 28** for full list of items’ loadings for the psychopathology bifactor model.

**Supplemental Figure 4.** Scree plot of eigenvalues of the initial exposome mixed correlation matrix of 96 environmental exposures in ABCD Study


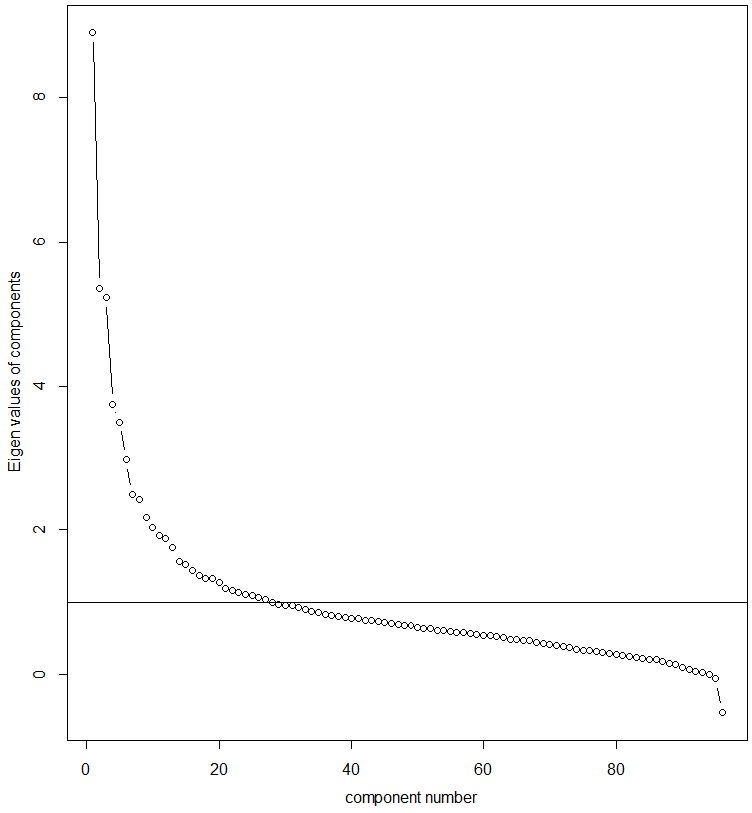


To determine the number of factors from the scree plot of eigenvalues, one can look for the “elbow” (point where the eigenvalues start to form a decreasing *linear* trend) or in cases where the location of the “elbow” is less clear (as here), one can look for “natural breaks” indicated by steep declines from one eigenvalue to the next. There are three such points here, suggesting 13, 8, and 6 factors (because the “elbows” occur at the 14^th^, 9^th^, and 7^th^ eigenvalues, respectively). See main text for explanation, but two reasons 6 factors were retained (rather than 13 or 8) in the present case were interpretability and practical scoring considerations (e.g., how many scores would be redundant in downstream analyses?).

**Supplemental Methods**

***Iterative exploratory factor analyses for exposome data reduction in ABCD Study***

We included a set of 348 environmental variables (both raw- and summary-level data) in the following analysis. Using multiple exploratory factor analyses (EFAs), we iteratively reduced the number of variables in our dataset. A sample first iteration is detailed below, which is representative of all following iterations.

1. Estimate a mixed correlation matrix where each bivariate relationship in the matrix is appropriate to the variable types. If two variables are continuous, use a Pearson correlation; if they are both dichotomous, use a tetrachoric correlation; if they are both ordinal (or one ordinal and one dichotomous), use polychoric; if one is continuous and the other dichotomous, use biserial; and if one is continuous and the other ordinal, use polyserial.

2. Determine the number of factors to extract based on subjective evaluation of the plot of descending eigenvalues (scree plot). That is, visually, subjectively determine where on the scree plot the decreasing function begins to form a linear trend (find the “elbow”). **Supplemental Figure 4** shows an example of a scree plot for determining the number of factors to extract.

3. Estimate an EFA model using least-squares extraction and oblimin rotation.

4. Examine the solution for interpretability, with particular attention to groups of variables so strongly related that they should be reduced. For example, if a factor comprised items from only one scale, with very high loadings on that factor and near-zero loadings elsewhere, that would suggest the scale could be reduced.

5. Use secondary factor analyses to reduce the groups of variables discovered in #4 above. For example, if all items from a checklist of negative life events loaded together in the solution in #4 above, submit that checklist to its own factor analysis. As in the main analysis, choose the number of factors based on subjective evaluation of the scree plot, calculate the appropriate correlation matrix (if a yes/no checklist, tetrachorics would be used), and use least-squares extraction with oblimin rotation.

6. Reduce the variables from #4 and #5 above by creating composite scores. In the present study, these composites were calculated using the following rules: a) if variables are dichotomous, take the mean to get a proportion endorsed; b) if variables are ordinal, z-transform them and take the mean; c) if variables are continuous, calculate factor scores (oblique Thurstone/regression method) from the model in #5 above.

7. Replace the variables discovered in #4 above with the variables created in #6 above. Using this updated data set, go back to #1 and repeat.

***Unique aspects of our approach to dimensionality reduction***

*Use of iterative target rotation*

Because we expected complex structure whereby some cross-loadings would be substantial and meaningful, we used iterated target rotation (ITR)^2,3^ rather than a simple structure rotation like oblimin or promax. Whereas simple structure rotations attempt to get p-1 elements in each row as close to zero as possible (where p = number of factors), ITR allows salient cross-loadings to be estimated freely. It starts with a simple structure rotation (here, oblimin), uses the resulting pattern matrix to determine not only which item loads where but also which cross-loadings might be non-negligible, and builds a partially-specified target matrix that incorporates cross-loading items^4^. Specifically, it uses a user-defined threshold (here, 0.20), sets all elements of the target matrix at 0 for items loading below that threshold, and sets all other (non-negligible) loadings to “unspecified” (indicating they should be estimated freely). The results of this target rotation are then used in the same way as the original simple structure rotation to specify a new target, and the process is repeated. When a new target matrix matches a previous target matrix in the iterative process, the ITR solution has converged.

*Use of exploratory and confirmatory factor analyses*

It is important to state why we used a confirmatory factor analysis (CFA) on the same sample as was used for the exploratory factor analyses (EFAs), whereas it’s typical to perform EFAs on a training sample to provide a configuration that CFA can then confirm in a separate sample. If we wished to make a claim about the “true” theoretical structure of the exposome, then a cross-validation framework would be optimal, as we have done in a previous work94. However, we conceptualize the exposome here as a bottom-up collection of phenomena that define it (the exposome) *ad hoc*. If additional variables were added to the analysis (e.g., prevalence of venomous snakes in the area or affordability of local fresh vegetables), the definition of the exposome itself would change. This is in contrast to, for example, depression, whose definition does not change when indicators are added; additional indicators simply increase the precision of measurement. In this sense, the goal of the present study was simply to calculate scores for use in downstream analysis (as shown in this study with the exposome factor scores’ association with psychopathology, obesity, and pubertal development), and confirmatory bifactor modeling allowed optimal estimation of those scores.

Furthermore, it is important to clarify why a confirmatory model was used to calculate factor scores as opposed to the original, exploratory model. CFA was used here because, as of this study, there is no good bifactor rotation available. The most common “bifactor” rotation, the Schmid-Leiman, is not a true bifactor. It estimates a higher-order solution and transforms that to a bifactor configuration, which necessitates proportionality constraints on the solution. Another option is the Jennrich-Bentler true bifactor rotation ^6^, which has been shown to perform poorly in multiple studies to date^7^. It is therefore preferred to use a confirmatory bifactor model to obtain scores.

*Use of a bifactor model*

It is important to explain the decision to use a bifactor model at all, given the weak inter-factor correlations found in the final EFA (see **Results**). Bifactor modeling accounts for inter-factor correlations by modeling the overall factor as its own phenomenon, unlike, for example, orthogonal EFA rotations (like varimax), which force orthogonality onto solutions without accounting for the true obliqueness of the phenomena. Usually, one of the indications that a bifactor model might be useful is moderate-to-strong inter-factors correlations, which suggest the existence of an overall, general factor underlying all item responses^8^. Here, inter-factor correlations were weak, suggesting that there may not be a hierarchical structure to environmental exposures (neither second-order nor bifactor). However, in addition to common sense suggesting that adverse environments at the distal level beget adverse environments at the proximal level, there is increasing evidence that bifactor general factors can contain critically important information even when inter-factor correlations are weak^9^. This is possible because, while the subfactors of a model might correlate only weakly, individual items within each subfactor may still load strongly on the general factor. The above-cited example demonstrates not only that such a phenomenon exists, but that the general factor scores generated from the seemingly ill-advised models have substantial validity.

**Supplemental References**

1. Rodriguez A, Reise SP, Haviland MG. Evaluating bifactor models: Calculating and interpreting statistical indices. *Psychological Methods*. 2016;21(2):137-150. doi:10.1037/met0000045

2. Moore TM. *Iteration of Target Matrices in Exploratory Factor Analysis (Doctoral Dissertation, UCLA)*.; 2013.

3. Moore T, Reise S, Depaoli S, Haviland M. Iteration of Partially Specified Target Matrices: Applications in Exploratory and Bayesian Confirmatory Factor Analysis. *Multivariate Behav Res*. 2015;50(2):149-161. doi:10.1080/00273171.2014.973990

4. Browne MW. OBLIQUE ROTATION TO A PARTIALLY SPECIFIED TARGET. *British Journal of Mathematical and Statistical Psychology*. 1972;25(2):207-212. doi:10.1111/J.2044-8317.1972.TB00492.X

5. Moore T, Kaczkurkin A, Durham E, et al. Criterion validity and relationships between alternative hierarchical dimensional models of general and specific psychopathology. *J Abnorm Psychol*. 2020;129(7):677-688. doi:10.1037/ABN0000601

6. Jennrich R, Bentler P. Exploratory bi-factor analysis. *Psychometrika*. 2011;76(4):537-549.

7. Mansolf M, Reise SP. Exploratory Bifactor Analysis: The Schmid-Leiman Orthogonalization and Jennrich-Bentler Analytic Rotations. *Multivariate Behavioral Research*. 2016;51(5):698-717. doi:10.1080/00273171.2016.1215898

8. Reise SP, Moore TM, Haviland MG. Bifactor Models and Rotations: Exploring the Extent to which Multidimensional Data Yield Univocal Scale Scores. *J Pers Assess*. 2010;92(6):544. doi:10.1080/00223891.2010.496477

9. Moore T. THREE FACTORS OF “PLANTNESS” –– TRAIT SUMMARY SCORES GENERATED FROM A BIFACTOR MODEL WITH COMPLEX STRUCTURE. *Phytoneuron*. 2020;66:1-25.
